# Supplementary material for: Protocol for a cluster-randomised non-inferiority trial of one versus two doses of ivermectin for the control of scabies using a mass drug administration strategy (the RISE study)
Source: BMJ Open. 2020 Aug 30;10(8):e037305. doi: 10.1136/bmjopen-2020-037305 (PMC7462236; doi:10.1136/bmjopen-2020-037305)
Supplement: Supplementary data [file bmjopen-2020-037305supp001.pdf]

Participant Information Statement and Consent Form

Initial Visit

HREC Project Number: 38099A

Research Project Title: RISE: Regimens of Ivermectin for Scabies Elimination

Local Principal Investigator: Mr Oliver Sokana

Version Number: 4

Version Date: 25/02/19

Thank you for taking the time to read this **Participant Information Statement and Consent Form**. We would like to invite you to participate in a research project that is explained below.

This document is 5 pages long. Please make sure you have all the pages.

What is an Information Statement and Consent Form?

An Information and Consent Form tells you about the research project. It clearly explains exactly what the research project will involve. This information is to help you decide whether or not you would like to take part in the research. Please read it carefully.

Before you decide if you want to take part or not, you can ask us any questions you have about the project. You may want to talk about the project with your family, friends or health care worker.

Taking part in the research is up to you

It is your choice whether or not you take part in the research. You do not have to agree if you do not want to. If you decide you do not want to take part, it will not affect the treatment and care you get.

Signing the form

If you want to take part in the research, please sign the consent form at the end of this document. By signing the form you are telling us that you:

- Understand what you have read
- Have had a chance to ask questions and received satisfactory answers
- Consent to taking part in the project. We will give you a copy of this form to keep.

Lake SJ, *et al. BMJ Open* 2020; 10:e037305. doi: 10.1136/bmjopen-2020-037305

1. What is the research project about?

The main aim of this project is to get rid of the skin infection known as scabies. People often don't know that they have this infection. To get the best results we try to treat everyone to make sure we don't miss anyone with infection. Usually you need to take the same medicine twice to get rid of this infection, but you may only need to take it once. We are trying to figure out if we can get rid of scabies if everyone in your community takes this medicine once.

The medicine that is used to treat scabies can also treat some kinds of intestinal worms. We are also trying to figure out whether treatment of intestinal worms is better with two doses of the medicine, compared to one dose.

2. Who is funding this research project?

The project is organised by the Solomon Islands Ministry of Health, the Murdoch Children's Research Institute, the London School of Hygiene & Tropical Medicine, The Kirby Institute and the Australian National University.

3. Why am I being asked to take part?

Twenty villages in the Western Province of the Solomon Islands have been selected at random to have their skin checked, give a sample of their stool to look for intestinal worms, and be provided with medication for scabies. Everyone living in the village will be asked to participate.

4. What do I need to do in this research project?

If you agree to take part, we will record information about your age and gender and will take your height and weight. We will examine your skin for signs of scabies and other skin problems.

Photographs may be taken of any skin lesions or rashes. These photos will not include your face or head and will not be recognisable as belonging to you. The research team will check with you before taking any photographs. If you do not wish to have a photograph taken that is fine.

We will ask you some questions about how scabies affects you and your family, and some questions about your risk of intestinal worm infections.

If you are willing to provide a stool sample, we will send your stool sample (without your name on it) to a laboratory at Murdoch Children's Research Institute in Australia to test for intestinal worm infections.

We will ask you if you are willing to take the treatment for scabies. If you agree, we will provide this treatment. For most people in the study this will be a tablet called ivermectin. For some people who can't have ivermectin, including children less than 90cm in height and pregnant women, treatment will be with a cream called permethrin. This cream only treats scabies, not intestinal worm infections.

If you live in a community that has been allocated two doses of treatment for scabies, we will visit you again to provide this second dose one or two weeks after the first dose.

We will visit you again to examine your skin for scabies and collect stool samples 12 months after our first visit, and then again 12 months after that. This is so we can check how effective the treatment is.

5. Can I withdraw from the project?

You can stop taking part in the project at any time. You just need to tell us so. You do not need to tell us the reason why. If you leave the project we will use any information already collected unless you tell us not to.

6. What are the possible benefits for me and other people in the future?

You and your community will receive treatment for scabies. If our study shows that this is an effective strategy, we may be able to provide this treatment to help other people in the Solomon Islands and many other countries.

7. What are the possible risks, side-effects, discomforts and/or inconveniences?

Treatment for scabies is very effective and side effects are uncommon and quickly go away.

Ivermectin occasionally causes dizziness or tummy upset. Children less than 90cm in height and pregnant women should not take ivermectin and will be offered a cream instead.

Permethrin cream occasionally causes itch and stinging.

Having your skin examined for scabies is not uncomfortable or painful. The whole process, including asking questions and examination should take less than 10 minutes.

You have previously been informed about stool collection procedures, at the time of receiving the stool collection kit.

8. What will be done to make sure my information is confidential?

Any information we collect for this project that can identify you will be treated confidentially, except as required by law. Nothing that could reveal your identity will be disclosed outside the project.

9. Will I be informed of the results when the research project is finished?

Results of the project will help us understand the best way to treat scabies in communities in the Solomon Islands and elsewhere. Results will be published in the medical literature, and a report summarizing the results will be sent to your community health worker who will pass on the

information to you. You and your family will not be personally identified in any report or publication.

10. Who should I contact for more information?

If you would like more information about the project, please contact:

Name: Mr Oliver Sokana

Contact telephone: 769 1615

Email: sokanao@moh.gov.sb

OR

Name: Prof. Andrew Steer

Contact telephone: +61 (3) 9345 5522

Email: Andrew.Steer@rch.org.au

If you:
- Have any concerns or complaints about the project
- Are worried about your rights as a research participant
- Would like to speak to someone independent of the project.

You can contact the Solomon Islands National Health Research Ethics Committee by telephone on (+677) 37295, or you can contact the Director of Research Ethics & Governance at The Royal Children's Hospital Melbourne by telephone on +61 (3) 9345 5044.

Lake SJ, *et al. BMJ Open* 2020; 10:e037305. doi: 10.1136/bmjopen-2020-037305

CONSENT FORM

HREC Project Number: 38099A

Research Project Title: RISE: Regimens of Ivermectin for Scabies Elimination

Local Principal Investigator: Mr Oliver Sokana

Version Number: 4

Version Date: 25/02/2019

- I have read this information statement and I understand its contents.
- I understand I have to do to be involved in this project.
- I understand the risks I could face because of my involvement in this project.
- I voluntarily consent to take part in this research project.
- I have had an opportunity to ask questions about the project and I am satisfied with the answers I have received.
- I understand that this project has been approved by The Solomon Islands National Health Research Ethics Committee and the Royal Children's Hospital Melbourne Human Research Ethics Committee. I understand that the project and any updates will be carried out in line with the National Statement on Ethical Conduct in Human Research (2007).
- I understand I will receive a copy of this Information Statement and Consent Form.

I consent/give consent for ________________________________ to take part in this study
Participant Name

☐ I consent/give consent for stool sample to be analysed for intestinal worm infections *(tick box if applicable)*

________________________________ ______________
Participant Signature or Fingerprint Date

________________________________ ________________________________ ______________
Name of Witness to Participant's Signature | Witness Signature | Date

Declaration by researcher: I have explained the project to the participant who has signed above. I believe that they understand the purpose, extent and possible risks of their involvement in this project.

________________________________ ________________________________ ______________
Research Team Member Name | Research Team Member Signature | Date

Note: All parties signing the Consent Form must date their own signature.

Lake SJ, *et al. BMJ Open* 2020; 10:e037305. doi: 10.1136/bmjopen-2020-037305
