# Supplementary material for: Protocol for a cluster-randomised non-inferiority trial of one versus two doses of ivermectin for the control of scabies using a mass drug administration strategy (the RISE study)
Source: BMJ Open. 2020 Aug 30;10(8):e037305. doi: 10.1136/bmjopen-2020-037305 (PMC7462236; doi:10.1136/bmjopen-2020-037305)
Supplement: Supplementary data [file bmjopen-2020-037305supp002.pdf]

SUPPLEMENTARY MATERIAL

Skin examination training for nurses

Training was delivered by two Australians doctors with experience in scabies and other tropical skin conditions. Training materials were developed based on material previously delivered in the Solomon Islands and Fiji (Table S1). The examination and history component of the training was focused on identifying the relevant features required for the diagnosis of scabies and impetigo. Other differential skin diagnoses that were relevant to the setting were also included.

Training consisted of two stages; classroom training and practical training at a primary school. The classroom training content included background information on the importance of scabies as a public health problem in the Solomon Islands, further context for the study, details on global and local prevalence of scabies, complications of the diseases and basic treatment concepts. Training on the diagnosis of scabies was based on the 2020 International Alliance for the Control of Scabies Consensus Criteria for the Diagnosis of Scabies.(1)

Clinical examination was limited to exposed areas of skin – particularly the arms from the mid-upper arm to the finger tips, and the legs from the mid-upper though to the toes. A brief history component containing questions about itch and contact history was incorporated. Terminology and definitions used in training were consistent with the World Health Organization 2018 training guide, "Recognizing neglected tropical diseases through changes on the skin*"*.(2)

Lake SJ, *et al. BMJ Open* 2020; 10:e037305. doi: 10.1136/bmjopen-2020-037305

Table S1 – Overview of training

**Part 1: Classroom training**
1.1 Scabies
What is scabies?
How do people get scabies?
How common is scabies?
What problems do scabies cause?
How can scabies be treated?
How can we get rid of scabies in the community?
1.2 Approach to diagnosis
About the skin
Dermatological terms
IACS criteria
History taking
Examination
Differential diagnoses
1.3 Facilitated practice with clinical images
**Part 2: Supervised field training**
2.1 Practice examination
**Part 3: Assessment**
3.1 Slide assessment
3.2 Field assessment

REFERENCES

1. Engelman D, Yoshizumi J, Hay RJ, Osti M, Micali G, Norton S, et al. The 2020 IACS Consensus Criteria for the Diagnosis of Scabies. British Journal of Dermatology. 2020.

2. World Health O. Recognizing neglected tropical diseases through changes on the skin: a training guide for front-line health workers. Geneva: World Health Organization; 2018 2018.
