# Supplementary material for: Protocol for a cluster-randomised non-inferiority trial of one versus two doses of ivermectin for the control of scabies using a mass drug administration strategy (the RISE study)
Source: BMJ Open. 2020 Aug 30;10(8):e037305. doi: 10.1136/bmjopen-2020-037305 (PMC7462236; doi:10.1136/bmjopen-2020-037305)
Supplement: Author's manuscript [file bmjopen-2020-037305.draft_revisions.pdf]

BMJ Open is committed to open peer review. As part of this commitment we make the peer review history of every article we publish publicly available.

When an article is published we post the peer reviewers' comments and the authors' responses online. We also post the versions of the paper that were used during peer review. These are the versions that the peer review comments apply to.

The versions of the paper that follow are the versions that were submitted during the peer review process. They are not the versions of record or the final published versions. They should not be cited or distributed as the published version of this manuscript.

BMJ Open is an open access journal and the full, final, typeset and author-corrected version of record of the manuscript is available on our site with no access controls, subscription charges or pay-per-view fees (http://bmjopen.bmj.com).

If you have any questions on BMJ Open's open peer review process please email info.bmjopen@bmj.com

BMJ Open

Protocol for a cluster randomised non-inferiority trial of one versus two doses of ivermectin for the control of scabies using a mass drug administration strategy (the RISE study)

Journal:	*BMJ Open*
Manuscript ID	bmjopen-2020-037305
Article Type:	Protocol
Date Submitted by the Author:	28-Jan-2020

Complete List of Authors:	Lake, Susanna; Murdoch Childrens Research Institute, Tropical Disease Research Group; The University of Melbourne, Department of Paediatrics<br>Phelan, Sophie; University of New South Wales, The Kirby Institute<br>Engelman, Daniel; Murdoch Childrens Research Institute, Tropical Disease Research Group; The University of Melbourne, Department of Paediatrics<br>Sokana, Oliver; Ministry of Health and Medical Services<br>Nasi, Titus; Ministry of Health and Medical Services<br>Boara, Dickson; Ministry of Health and Medical Services<br>Gorae, Christina; Ministry of Health and Medical Services<br>Schuster, Tibor; McGill University, Clinical Epidemiology and Biostatistics Unit<br>Grobler, Anneke; Murdoch Childrens Research Institute, Clinical Epidemiology and Biostatistics Unit<br>Osti, Millicent; Murdoch Childrens Research Institute, Tropical Disease Research Group<br>Andrews, Ross; Australian National University<br>Marks, Michael; London School of Hygiene and Tropical Medicine, Clinical Research Department; Hospital for Tropical Diseases,<br>Whitfeld, Margot; St Vincent's Hospital , Department of Dermatology<br>Romani, Lucia; University of New South Wales, The Kirby Institute<br>Kaldor, John; University of New South Wales, The Kirby Institute<br>Steer, Andrew; Murdoch Childrens Research Institute, Tropical Disease Research Group; The University of Melbourne, Department of Paediatrics

Keywords:	TROPICAL MEDICINE, INFECTIOUS DISEASES, DERMATOLOGY, International health services < HEALTH SERVICES ADMINISTRATION & MANAGEMENT

SCHOLARONE™
Manuscripts

I, the Submitting Author has the right to grant and does grant on behalf of all authors of the Work (as defined in the below author licence), an exclusive licence and/or a non-exclusive licence for contributions from authors who are: i) UK Crown employees; ii) where BMJ has agreed a CC-BY licence shall apply, and/or iii) in accordance with the terms applicable for US Federal Government officers or employees acting as part of their official duties; on a worldwide, perpetual, irrevocable, royalty-free basis to BMJ Publishing Group Ltd ("BMJ") its licensees and where the relevant Journal is co-owned by BMJ to the co-owners of the Journal, to publish the Work in this journal and any other BMJ products and to exploit all rights, as set out in our licence.

The Submitting Author accepts and understands that any supply made under these terms is made by BMJ to the Submitting Author unless you are acting as an employee on behalf of your employer or a postgraduate student of an affiliated institution which is paying any applicable article publishing charge ("APC") for Open Access articles. Where the Submitting Author wishes to make the Work available on an Open Access basis (and intends to pay the relevant APC), the terms of reuse of such Open Access shall be governed by a Creative Commons licence – details of these licences and which Creative Commons licence will apply to this Work are set out in our licence referred to above.

Other than as permitted in any relevant BMJ Author's Self Archiving Policies, I confirm this Work has not been accepted for publication elsewhere, is not being considered for publication elsewhere and does not duplicate material already published. I confirm all authors consent to publication of this Work and authorise the granting of this licence.

For peer review only

Protocol for a cluster randomised non-inferiority trial of one versus two doses of ivermectin for the control of scabies using a mass drug administration strategy (the RISE study)

Susanna J. Lake MIPH (1,2,3)

Sophie L. Phelan MIPH MAE (4)

Daniel Engelman PhD (1,2,3)

Oliver Sokana MPH(5)

Titus Nasi MCH(5)

Dickson Boara PgDipHSM (5)

Christina Gorae (5)

Tibor Schuster PhD (6)

Anneke C. Grobler PhD (7)

Millicent H. Osti MBBS (1,2)

Ross Andrews PhD (8)

Michael Marks PhD (9,10)

Margot J. Whitfeld FACD (11)

Lucia Romani PhD (4)

John M. Kaldor PhD (4)

Andrew C. Steer PhD (1,2,3)

1. Tropical Diseases Research Group, Murdoch Children's Research Institute, Melbourne, Australia
2. Department of Paediatrics, University of Melbourne, Melbourne, Australia
3. Melbourne Children's Global Health, Melbourne, Australia
4. Kirby Institute, University of New South Wales, Sydney, Australia

For peer review only

5. Ministry of Health and Medical Services, Solomon Islands

6. McGill University, Montreal Canada

7. Clinical Epidemiology and Biostatistics Unit, Murdoch Children's Research Institute, Melbourne, Australia

8. Australian National University, Canberra, Australia

9. London School of Hygiene and Tropical Medicine, London, United Kingdom

10. Hospital for Tropical Diseases, London, United Kingdom

11. Department of Dermatology, St Vincent's Hospital, Sydney, Australia

Correspondence to:

Professor Andrew C Steer; Andrew.Steer@rch.org.au

Keywords:

Scabies

Mass drug administration

Ivermectin

Solomon Islands

For peer review only

ABSTRACT

Introduction

Scabies is a significant contributor to global morbidity, affecting approximately 200 million people at any time. Scabies is endemic in many resource-limited tropical settings. Bacterial skin infection (impetigo) frequently complicates scabies infestation in these settings. Community-wide ivermectin-based mass drug administration (MDA) is an effective control strategy for scabies in island settings, with a single round of MDA reducing population prevalence by around 90%. However, current two-dose regimens present a number of barriers to programmatic MDA implementation. We designed the Regimens of Ivermectin for Scabies Elimination (RISE) trial to investigate whether one-dose MDA may be as effective as two-dose MDA in controlling scabies in high-prevalence settings.

Methods and analysis

RISE is a cluster randomised non-inferiority trial. The study will be conducted in 20 isolated villages in Western Province of Solomon Islands where population prevalence of scabies is approximately 20%. Villages will be randomly allocated to receive either one dose or two doses of ivermectin-based MDA in a 1:1 ratio. The primary objective of the study is to determine if ivermectin-based MDA with one dose is as effective as MDA with two doses in reducing the prevalence of scabies after 12 months. Secondary objectives include the effect of ivermectin-based MDA on impetigo prevalence after 12 months and 24 months, the prevalence of scabies at 24 months after the intervention, the impact on presentations to health facilities with scabies and impetigo, and the safety of one-dose and two-dose MDA.

For peer review only - http://bmjopen.bmj.com/site/about/guidelines.xhtml

Ethics and dissemination

This trial has been approved by the ethics review committees of the Solomon Islands and the Royal Children's Hospital, Australia. Results will be disseminated in peer-reviewed publications and in meetings with the Solomon Islands Ministry of Health and Medical Services and participating communities.

Trial Registration

Australian New Zealand Clinical Trials Registry: ACTRN126180011086257. Date registered: 28 June, 2018.

For peer review only

For peer review only - http://bmjopen.bmj.com/site/about/guidelines.xhtml

ARTICLE SUMMARY

Strengths and limitations of this study

- The results of this study will inform the way scabies is controlled in populations where it is endemic
- The cluster randomised study design follows the implementation of the intervention at a village level
- Follow up at both 12 and 24 months will demonstrate longer term effects of the intervention
- This study is being conducted in partnership with the Solomon Islands Ministry of Health and Medical Service and will build the capacity of local nursing staff as well as being conducted in a culturally sensitive manner
- Scabies prevalence is high in the isolated island villages where this study will be conducted (approximately 20%), therefore results may not be transferable to lower prevalence or urban settings.

For peer review only

BACKGROUND

Scabies is a neglected tropical disease (NTD) caused by infestation with the mite *Sarcoptes scabiei* var. *hominis*. Scabies is a significant contributor to global morbidity, estimated to cause 455 million annual incident cases.(1, 2) Transmission occurs as a result of skin-to-skin contact and is more common in overcrowded settings, including in many tropical environments where crowding and poverty are prevalent and access to treatment limited.(3) The burden of disease is substantial in many Pacific Island Countries where scabies affects 1 in 5 people and up to 1 in 2 children.(4)

Scabies infestation causes intense itch and discomfort. Furthermore, it is responsible for a considerable proportion of bacterial skin infection (impetigo) in many resource-limited settings.(5-7) Scabies causes a breach in the skin barrier from scabetic lesions and subsequent scratching, creating an entry point for bacteria including *Staphylococcus aureus* and *Streptococcus pyogenes.* Resulting impetigo can in turn cause severe infection and immune-mediated disease including sepsis, glomerulonephritis and possibly rheumatic fever.(8-11)

Treatment guidelines for scabies recommend treatment of the infected individual as well as household contacts.(12, 13) Most guidelines recommend treatment with topical acaracides such as permethrin or benzoyl benzoate.(12, 13) These medications are effective, if applied to all affected areas for the correct duration, but re-infestation frequently occurs in highly endemic settings where individuals may be exposed to infected household or community members, many of whom may be asymptomatic.(14) Therefore, attention has shifted to simultaneous treatment of whole communities, including those without symptoms of infestation, to reduce prevalence and the rate of transmission. (15) This strategy of mass drug

For peer review only - http://bmjopen.bmj.com/site/about/guidelines.xhtml

administration (MDA) has been used to successfully control a number of NTDs including onchocerciasis, lymphatic filariasis (LF), trachoma and soil-transmitted helminths and there is a growing body of evidence to support MDA for scabies control.(16-21)

Ivermectin is an antiparasitic drug in the avermectin class that is active against the scabies mite. Ivermectin-based MDA for scabies involves offering ivermectin treatment to the whole community, with the exception of young children, pregnant women and others with a contraindication to ivermectin. Permethrin cream is offered as an alternative to ivermectin for these groups. Several studies in Pacific Island Countries with high-prevalence have shown ivermectin-based MDA can reduce the population prevalence of scabies by around 90%.(6, 16, 22) The SHIFT study in Fiji was the first comparative study to demonstrate the effectiveness of ivermectin MDA for scabies control, finding a reduction in the population prevalence of scabies from 32% at baseline to less than 2% at 12 months.(20) These trials have all used an MDA strategy involving two doses of medication, given 7-14 days apart (either to the whole community, or those with clinical signs of scabies).(6, 22) This is consistent with clinical recommendations for treatment of individuals.(12) Ivermectin is known to lack ovicidal activity, therefore the second dose aims to kill newly hatched mites.(23)

In 2017, the World Health Organization (WHO) recognised scabies as a NTD, and identified the need for public health action to control scabies in endemic settings.(8) The Strategic and Technical Advisory Group on Neglected Tropical Diseases called for further research into control strategies for scabies and the development of guidelines for the public health use of avermectins.(24)

For peer review only - http://bmjopen.bmj.com/site/about/guidelines.xhtml

For peer review only

While ivermectin-based MDA shows great promise as a control strategy for scabies, the requirement for two doses of medication at each MDA round presents barriers to implementation. Drug and implementation costs are doubled compared to single-dose MDA. Distribution is more complex and integration with programs for other NTDs is difficult. These hurdles may be prohibitive to wide-spread implementation of scabies control, particularly in low-income settings. Therefore, the optimum dosing strategy for MDA remains an important knowledge gap.(8) For this reason we designed the Regimens of Ivermectin for Scabies Elimination (RISE) trial.

For peer review only

METHODS AND ANALYSIS

Study Design

The RISE trial is a prospective, open-label comparison of one dose versus two doses of ivermectin-based MDA for the population-level control of scabies. Using a cluster-randomised design, 20 villages will be randomised to one of two intervention groups in a 1:1 ratio. Randomisation will occur at the village level, rather than the individual level, as the objective is to determine the dosing regimen for controlling scabies within whole communities. Randomisation minimises the possibility of the anticipated difference in the outcome between each group being confounded. A two-dose regimen is an appropriate comparator (rather than no-treatment or placebo) as two-doses of ivermectin-based MDA is the currently accepted dosing regimen. We chose a non-inferiority design because it is unlikely that a one-dose regimen would be superior in effectiveness to a two-dose regimen. However, the logistic and pragmatic advantages of a one-dose regimen compared to a two-dose regimen make a non-inferior study appealing.

The prevalence of scabies and impetigo will be measured before the intervention (baseline), and repeated at 12 months and 24 months after the intervention. To measure a secondary outcome the standard Solomon Islands Ministry of Health and Medical Services (MHMS) health facility reporting processes will be used to capture the number of presentations to health facilities with scabies and impetigo in the study catchment area for the 12 month period before the intervention and for the 24 month period after the intervention.

For peer review only - http://bmjopen.bmj.com/site/about/guidelines.xhtml

Aims

The primary objective of this study is to determine if ivermectin-based MDA with one dose is non-inferior to two doses in reducing the prevalence of scabies 12 months after the intervention. The secondary objectives are to assess the impact of ivermectin-based MDA on: population prevalence of scabies after 24 months; population prevalence of impetigo after 12 months and 24 months; the number of presentations to health clinics with scabies and impetigo before and after the intervention; the number of adverse events measured by passive surveillance in the 12-months after MDA in each study group.

Rationale

This study uses ivermectin-based MDA because the SHIFT trial in Fiji demonstrated the greatest reduction in scabies prevalence after ivermectin-based MDA.(20) Ivermectin is currently the only oral therapy available for scabies and it allows greater compliance than with topical therapy. Oral therapy can be directly observed, ensuring adherence to treatment. Although ivermectin only kills the mature scabies mite and not the eggs, a single dose of treatment simultaneously administered to a whole village may reduce transmission sufficiently to reduce population prevalence. A recent Cochrane review did not find a difference in efficacy of one dose of oral ivermectin compared to two doses of oral ivermectin, but confidence in the effect estimates was low to moderate, with poor reporting a major limitation.(25) A retrospective study in Zanzibar of six rounds of annual single dose ivermectin MDA for LF showed a 68-98% decline in clinical presentations and treatments for scabies, suggesting a one-dose strategy may significantly reduce transmission.(26) By

For peer review only - http://bmjopen.bmj.com/site/about/guidelines.xhtml

contrast, annual ivermectin MDA for LF did not reduce the prevalence of scabies in Tanzania where the baseline prevalence was less than 5%.(27)

Study setting and participants

The study will be conducted in Western Province of Solomon Islands (Figures 1 and 2). Solomon Islands is a nation in the South Pacific with a population of over 650,000 spread across 900 islands, a geography that presents many challenges for health service delivery.(28) Solomon Islands is classified as a least developed country. It is ranked 152 out of 189 on the Human Development Index.(29) The majority of the population depend on subsistence agriculture in rural locations. We chose Western Province for this study for several reasons: first, there is a high burden of scabies - a 2014 survey estimated an all-age scabies prevalence of 19.2%;(5) second, we expect that there will be relatively little mixing between villages because of the island geography of isolated villages with no road transport; third, there are many villages of appropriate population size (between 180 and 300) for the cluster-randomised design.

Twenty villages will be selected after close consultation with the MHMS. Criteria for selecting the villages include a population of between 180 and 300 people, geographic isolation and willingness to participate in the study. All residents of the 20 selected villages will be eligible to participate. If a resident of a two-dose village does not take the first dose of medication they will still be eligible to take a dose when the team returns to the village for the second dose.

For peer review only

This is a community-based study, with analysis conducted at the village level, and therefore all residents are eligible to participate in the follow-up assessment at 12 months and 24 months, regardless of whether they received treatment at baseline. Written informed consent will be obtained from all participants. Participants under the age 18 years will require written consent to be provided by a parent or guardian. Consent will be obtained at each study timepoint (baseline, 12 months and 24 months).

Inclusion and exclusion criteria

Inclusion criteria. All participants who provide consent are eligible to participate. If exclusion criteria for treatment are met, then consented participants are still eligible to have their skin examined.

Exclusion criteria. Participants who meet any of the following criteria will not receive treatment, but will be eligible to enrol in the study and undergo skin examination: allergy to ivermectin or permethrin; treatment within the last 7 days with ivermectin or permethrin; declines treatment. Participants with severe acute or chronic illness will not be treated with ivermectin but may receive permethrin.

Patient and public involvement statement

The trial was designed in close consultation with stakeholders at the Solomon Islands MHMS to ensure it was culturally appropriate for the local setting. Staff from the Solomon Islands MHMS contributed to study design and identification of study sites. The study team will

For peer review only

comprise a majority of Solomon Islander staff from Western Province. Staff are able to communicate in the local regional languages.

A team of health promotion officers from the Solomon Islands MHMS will conduct community awareness in each village, approximately one month prior to MDA. An illustrated information leaflet outlining the study design as well as information about scabies and the treatments will be provided during community visits. A participant information statement that contains contact details for the principal investigator and local investigator will be made available to all village residents. Community awareness and the informed consent process will be conducted in Solomon Islands Pijin and study staff who speak the local regional language will be available to provide further information or clarification as required. Results of the study will be communicated to community leaders and members by the study team.

Intervention

Oral ivermectin will be offered to all participants, unless there is a contraindication to ivermectin. The contraindications for ivermectin are: pregnancy; breastfeeding an infant less than seven days old; age less than two years; height less than 90 cm; concurrent medication that may interact with ivermectin (for example, warfarin); or severe acute or chronic illness on the day of MDA. If ivermectin is contraindicated, then topical permethrin will be offered.

A dose of 200 μg/kg of ivermectin is recommended for the treatment of individuals with scabies.(12, 30) We will aim to dose ivermectin within a range of 150-250 μg/kg, as this dose has been effective in previous trials.(20) We will use 6 mg scored tablets. Doses will be rounded to the nearest 3 mg. The tablets will be accurately halved on the score line using a

For peer review only - http://bmjopen.bmj.com/site/about/guidelines.xhtml

pill cutter as required. As weight scales are generally unsuitable for implementation of MDA, we will use dosing strategies appropriate for larger scale programmatic roll-out.

We will use height-based dosing for children aged less than 15 years, with doses ranging from 3 mg to 12 mg, as is standard for MDA for onchocerciasis and lymphatic filariasis.(31, 32) Adults will receive a standard dose of 12 mg, with doses adjusted based on visual assessment of body shape (9 mg for adults assessed to be a malnourished, 15 mg for adults assessed to be obese). Drug distribution staff will make these assessments based on a series of body shape silhouettes.(33) Dosing of medications for MDA based on physical appearance has been shown to be accurate and safe.(32) Staff will undergo training and validation for these dosing techniques. Ivermectin will be administered by trained study staff who will directly observe swallowing of the tablets.

Topical permethrin 5% cream will be given to participants meeting exclusion criteria for ivermectin. Permethrin will be dosed according to clinical guidelines.(34) Participants or carers will be counselled to apply the cream to the whole body from the neck down (in infants cream should also be applied to the scalp) and to leave it for 8 to 14 hours, or 4 hours in infants less than 2 months of age.

Outcome measures

All participants will undergo assessment for symptoms and signs of scabies, impetigo and other skin disease.(35) Assessment will include history questions regarding the presence of itch, contact history and a simplified skin examination. Skin examination will be limited to areas that are usually exposed (arms from above elbow to fingers, legs from above knee to

For peer review only - http://bmjopen.bmj.com/site/about/guidelines.xhtml
For peer review only

toes, head and neck). Other areas (including breasts, groin or genitals) will not be examined. Data suggest that in this setting a limited examination detects more than 90% cases of scabies.(35) The skin of children less than 2 years age will be examined more generally, as scabies may be more widespread in this age group.(3)

Scabies will be diagnosed according to consensus criteria established by the International Alliance for the Control of Scabies (IACS).(36) Categorisation will be based on the identification of typical scabies lesions, typical body distribution of lesions and presence of itch and/or positive contact history. Diagnosis will therefore use levels B (Clinical Scabies) and C (Suspected Scabies) (See Table 1). Confirmation of diagnosis with microscopy or dermoscopy is not feasible in this remote setting. Impetigo will be recorded if papular, pustular or ulcerative lesions surrounded by erythema, or with crusts, pus or bullae are seen.(6) This approach is consistent to diagnostic processes in previous scabies community intervention trials. Examinations will be conducted by nurses from Western Province. Nurses will receive one week of theoretical and practical training in the clinical assessment for scabies and impetigo, including application of the IACS diagnostic criteria.(37) Nurses will receive additional training prior to the follow-up surveys at 12 and 24 months.

Other severe skin infections such as ulcers, abscesses or suspected cases of crusted scabies will also be recorded where noted. If these, or other significant medical conditions are noted during the survey, participants will be referred off-study to the local health clinic for assessment and management.

In addition to skin examination data we will also collect information on presentations to health facilities in the study villages. Government health facilities in Solomon Islands

For peer review only

routinely record the details of all attendances and admissions in paper-based registers. Cases of scabies, local and serious bacterial infections and other skin diseases are recorded. Facilities report aggregated data using a standardised form each month. Data is transferred electronically through the District Health Information System (DHIS2) to the MHMS Health Information Statistics Unit. We will use the information from DHIS2 to assess the number of presentations to health facilities with scabies and impetigo. Data collected in the 12 months prior to MDA will be compared to data collected in the 24 months following MDA.

Safety monitoring and reporting

Ivermectin is well tolerated and has a significant dose safety margin with no safety concerns at much higher doses than clinically required (up to 120 mg in adults, approximately 2000 µg/kg).(38, 39) Over one billion doses have been distributed for control of onchocerciasis and lymphatic filariasis with few effects reported beyond minor, reversible events.(40, 41) There have been cases of encephalopathy following ivermectin administration but these have been in the context of loiasis, a disease which has not been detected in Solomon Islands.(42) Ivermectin is on the WHO Model Essential Medicines List and the Solomon Islands Essential Medicines List for the treatment of scabies.(30, 43) Although topical benzyl benzoate is the standard treatment of scabies in the Solomon Islands, topical 5% permethrin will be used in the study due to its increased efficacy and lower rate of local side effects.(44) Permethrin is well tolerated with very few side effects, including in infants.(45, 46) Nonetheless, we will record all reported adverse events related to treatment using passive monitoring.

Participants will be advised to report any adverse events to clinic nurses or directly to the study team if the adverse event occurs immediately post MDA. The clinic nurses will relay

For peer review only

information to the study coordinator who will document the adverse event and send a report to the Principal Investigator who will in turn collate adverse events for reporting to the Data Safety Monitoring Board (DSMB). Any serious adverse events or suspected serious adverse reactions will be reported to the study coordinator by the study team, or clinical staff at the clinics and hospitals in the study area. Hospitals will be briefed on the study and provided with comprehensive reporting forms and the MDA schedule. Hospitals will report any admissions or deaths from study villages for one month following administration of the first dose of ivermectin. We will retrospectively review mortality records to ensure all deaths from study villages have been captured. We will review routinely collected summary data on all stillbirths from hospitals in the area for 12 months following MDA.

An independent DSMB will provide oversight to the safety and progress of the trial. The DSMB will meet via teleconference prior to the study, in the first three months after MDA and at the conclusion of the study. Any serious adverse events and suspected serious adverse reactions will be reported to the DSMB within seven days.

Sample size

Sample size calculations were based on scabies prevalence in Western Province of Solomon Islands and the effect size measured in previous studies of ivermectin-based MDA for scabies.(5, 16, 20) A standard Monte Carlo simulation method with 1000 repetitions was used to estimate the required sample size and number of villages to achieve statistical power of 80%.(47) We assumed that scabies prevalence across villages would range from 10% to 30% (mean 20%, standard deviation, SD, 5%) at baseline (5). The effect size measured in previous studies with two doses of ivermectin MDA was used to assume the prevalence of

For peer review only - http://bmjopen.bmj.com/site/about/guidelines.xhtml

scabies 12 months after MDA will be between 3% and 9% (mean 6%, SD 2%) in the one-dose group and between 1% and 5% (mean 3%, SD 1%) in the two-dose group (16, 20). We assumed an average village size of 250 people with a range of 200 to 300.(48) We considered a non-inferiority margin of 5% (prevalence of scabies at 12 months in the one-dose group minus prevalence at 12 months in the two-dose group) to be relevant from a public health perspective. Based on these assumptions, 20 villages, randomised equally, would be sufficient to achieve the required power.

Randomisation

An independent statistician will randomise villages to the one-dose or two-dose group in a 1:1 ratio once the 20 study villages have agreed to participate. There will be no stratification within the randomisation process. There will be 10 villages in each group (Figure 3).

Analysis plan

We will account for clustering when calculating all study outcomes by calculating outcomes at the cluster level and analysing data at the cluster level and not the individual level.(49) The range of cluster-level outcomes will be reported by group.

Primary outcome

The prevalence of scabies in each village will be calculated at baseline (0 months), and 12 months. The prevalence will be calculated by dividing the number of participants with scabies by the denominator (the total number of participants examined for scabies) in each

For peer review only

cluster. The denominator will vary at each timepoint as we will include all participants who consented for skin examination, regardless of their involvement at other timepoints.

The difference in scabies prevalence between baseline and 12 months will be calculated for each village. The means of these differences will be calculated in the two treatment groups and compared by calculating the difference between the means. If the upper limit of the two-sided 95% confidence interval of the mean difference between the two study groups is less than or equal to 5% (the clinically relevant non-inferiority margin) the one-dose regimen will be considered non-inferior.

Secondary outcomes

The analysis for the prevalence of scabies at 24 months and impetigo at 12 and 24 months will be done in the same way as for the primary endpoint.

The change in the number of presentations to health facilities for scabies and impetigo will be analysed in three ways. First, the total number of presentations in the 12 months before MDA will be compared to the number of presentations in months 1 to 12 and 13 to 24 after MDA. Second, we will calculate the proportion of clinic presentations for scabies and impetigo by dividing the number of presenations for scabies and impetigo by the total number of clinic presentations for any condition. We will calculate this proportion for the 12 months before MDA, 1 to 12, and 13 to 24 months after MDA. Calculating the proportion will account for any changes in population size or operational status of health facilities. Third, we will compare the number of clinic presentations for scabies and impetigo in the clinics that service the study villages and compare this with clinic presentation for scabies and impetigo in other clinics in the province, this will be adjusted for population size.

For peer review only - http://bmjopen.bmj.com/site/about/guidelines.xhtml

The number of adverse events in each study group will be calculated as a proportion of the total number of participants in each study group that received MDA at baseline. We will also report the number of deaths in the month following MDA in each study group as a proportion of the number of participants in each group.

Data collection and management

Data will be collected using a combination of paper-based and electronic forms. Paper forms will be stored in locked filing cabinets. Information will be deidentified and participant names will only be recorded on consent forms. Only authorised study staff will be able to access forms. Data will be destroyed after 15 years in compliance with local guidelines. Skin examination data will be collected and managed using REDCap electronic data capture tools hosted at Murdoch Children's Research Institute.(50, 51) REDCap is a secure, web-based software platform designed to support data capture for research studies.

Trial Status

Baseline data collection and MDA took place between May and July 2019. A total of 5,260 participants were enrolled. Follow-up village data collection is scheduled to take place between May and July 2020 and between May and July 2021.

Ethics and dissemination

For peer review only - http://bmjopen.bmj.com/site/about/guidelines.xhtml

The RISE trial is investigator-initiated and funded by the National Health and Medical Research Council of Australia (GNT1127297). The funder was not involved in protocol development or the study process including site selection, management, data collection or analysis of the results. The trial is a collaboration between the Murdoch Children's Research Institute, the Solomon Islands MHMS, the Kirby Institute at the University of New South Wales, the London School of Hygiene and Tropical Medicine and the Australian National University.

The trial was designed in accordance with CONSORT guidelines and our reporting of the protocol conforms to the Standard Protocol Items: Recommendations for Interventional Trials (SPIRIT) 2013 checklist.(52-54) This trial has been approved by the Solomon Islands Health Research and Ethics Review Board (HRE005/18) and Royal Children's Hospital Human Research Ethics Committee (38099A) in Melbourne, Australia and will be conducted in accordance with Good Clinical Practice.

De-identified data may be made available for further analysis with appropriate approvals. Results of the study will be presented locally and made available to health policy decision makers and clinical staff. Villages that participated in the trial will be presented the results in a culturally appropriate way that is easy to understand and interpret. Participants will have the opportunity for results to be explained to them in their own language.

For peer review only

DISCUSSION

Scabies is a common disease in many tropical and low-income settings and has been prioritised for control by WHO, but there are still gaps in knowledge to determine the optimum approach to control in settings where scabies is highly endemic.(8) The results of this trial will have an impact on national, regional and global strategies for scabies control.

Island communities in the Pacific have among the highest global prevalence of scabies and understanding how to implement MDA in these settings has the potential for translation into huge public health impact for these communities.(4) However, the results may not be generalisable to populations with a much lower prevalence of the disease, to settings with higher population density, or to urban settings. This trial is designed to assess a single round of MDA, there is scope for further research to assess the efficacy of repeated annual rounds of MDA. The cluster-randomised design will allow analysis of the impact of MDA at the community level. We will be able to assess the impact of the intervention on the whole community, even for those who will not receive MDA.

If the RISE trial finds that one-dose ivermectin MDA is inferior, then the need for two doses of ivermectin-based MDA would need to be taken into account in decision-making around control strategies for scabies. It would also provide impetus for further research to identify new treatments for scabies that may be able to be implemented with one dose. Approaches may include novel treatments that are ovicidal, or other medications with a longer half-life, such as moxidectin(55). If one-dose ivermectin-based MDA is found to be non-inferior to two-dose then this strategy will be highly attractive for implementation as a public health

For peer review only

program. The lower cost, simplified logistics and ability to integrate with other programs would make scabies control programs more feasible in low-income settings.

For peer review only

Funding

This research is funded by the National Health and Medical Research Council of Australia (NHMRC). DE, LR, JK and ACS are supported by fellowships from the NHMRC. MM is supported by the UK National Insitute for Health Research.

Acknowledgements

The authors are grateful to the Solomon Islands MHMS and the communities who will participate. We appreciate the support and contributions of Pauline McNeil, Nemia Bainivalu, Gregory Jilini, Michael Larui, Ivan Ghemu, Freda Pitakaka, William Horoto, Jeffrey Korini, Soraya Pina, Yvonne Tuni, Selina Maena, Frederick Neqo, Susana Vaz Nery and Naomi Clarke. The RISE study team includes: Sana Bisili, Sharmillah Jack, Arthur Keremama, Erica Lazu, Relinta Manaka, Davis Pesala, Deanne Seppy, Winter Sino, Patson Solomon, Stephen Tiazi, and Salote Wickham.

This study is dedicated to Dr Tenneth Dalipanda, former Permanent Secretary of the Solomon Islands MHMS. Dr Tenneth was committed to improving the health of Solomon Islanders. He was an advocate for public health research and without his support we would not have been able to conduct this research and many studies before it.

For peer review only

Author contributions

Study concept and design were conducted by the investigators: ACS, DE, JMK, LR, MW, MM, RA, TS, OS and TN. Critical revision of concept and design and intellectual input in the study protocol was done by all authors. Drafting of the protocol was done by SLP and ACS, with review by all authors. Drafting of the manuscript was done by SJL. Critical revision of the manuscript was performed by all authors. Study supervision is conducted by the investigators.

For peer review only

Competing interests

None declared

Word count:

4267

REFERENCES

1. Vos T, Abajobir AA, Abate KH, Abbafati C, Abbas KM, Abd-Allah F, et al. Global, regional, and national incidence, prevalence, and years lived with disability for 328 diseases and injuries for 195 countries, 1990–2016: a systematic analysis for the Global Burden of Disease Study 2016. The Lancet. 2017;390(10100):1211-59.
2. Karimkhani C, Colombara DV, Drucker AM, Norton SA, Hay R, Engelman D, et al. The global burden of scabies: a cross-sectional analysis from the Global Burden of Disease Study 2015. The Lancet Infectious Diseases. 2017;17(12):1247-54.
3. Hardy M, Engelman D, Steer A. Scabies: A clinical update. Australian Family Physician. 2017;46(5):264-8.
4. Romani L, Steer AC, Whitfeld MJ, Kaldor JM. Prevalence of scabies and impetigo worldwide: a systematic review. Lancet Infect Dis. 2015;15(8):960-7.
5. Mason DS, Marks M, Sokana O, Solomon AW, Mabey DC, Romani L, et al. The Prevalence of Scabies and Impetigo in the Solomon Islands: A Population-Based Survey. PLoS Neglected Tropical Diseases [electronic resource]. 2016;10(6):e0004803.
6. Romani L, Koroivueta J, Steer AC, Kama M, Kaldor JM, Wand H, et al. Scabies and impetigo prevalence and risk factors in Fiji: a national survey. PLoS Negl Trop Dis. 2015;9(3):e0003452.
7. Aung PTZ, Cuningham W, Hwang K, Andrews RM, Carapetis JR, Kearns T, et al. Scabies and risk of skin sores in remote Australian Aboriginal communities: A self-controlled case series study. PLoS Negl Trop Dis. 2018;12(7):e0006668.
8. Engelman D, Cantey PT, Marks M, Solomon AW, Chang AY, Chosidow O, et al. The public health control of scabies: priorities for research and action. Lancet. 2019;394(10192):81-92.
9. Parks T, Smeesters PR, Steer AC. Streptococcal skin infection and rheumatic heart disease. Curr Opin Infect Dis. 2012;25(2):145-53.
10. Thornley S, Marshall R, Jarrett P, Sundborn G, Reynolds E, Schofield G. Scabies is strongly associated with acute rheumatic fever in a cohort study of Auckland children. J Paediatr Child Health. 2018;54(6):625-32.
11. Tasani M, Tong SY, Andrews RM, Holt DC, Currie BJ, Carapetis JR, et al. The Importance of Scabies Coinfection in the Treatment Considerations for Impetigo. Pediatr Infect Dis J. 2016;35(4):374-8.
12. eTG complete. [Internet]. Melbourne, Victoria: Therapeutic Guidelines Ltd. Available from: https://tgldcdp.tg.org.au.acs.hcn.com.au/viewTopic?topicfile=insects-mites&guidelineName=Dermatology#toc_d1e110.
13. WHO Model Formulary for Children. Geneva, Switzerland: World Health Organisation; 2010.
14. David, Lucinda. A Review of Scabies: An Infestation More than Skin Deep. Dermatology. 2019;235(2):79-90.
15. Engelman D, Steer A. Control Strategies for Scabies. Tropical Medicine and Infectious Disease. 2018;3(3):98.
16. Romani L, Marks M, Sokana O, Nasi T, Kamoriki B, Cordell B, et al. Efficacy of mass drug administration with ivermectin for control of scabies and impetigo, with coadministration of azithromycin: a single-arm community intervention trial. The Lancet Infectious Diseases. 2019;19(5):510-8.
17. Marks M, Taotao-Wini B, Satorara L, Engelman D, Nasi T, Mabey DC, et al. Long term control of scabies fifteen years after an intensive treatment programme. PLoS Negl Trop Dis. 2015;9(12):e0004246.
18. Taplin D, Porcelain SL, Meinking TL, Athey RL, Chen JA, Castillero PM, et al. Community control of scabies: a model based on use of permethrin cream. Lancet. 1991;337(8748):1016-8.

For peer review only - http://bmjopen.bmj.com/site/about/guidelines.xhtml

19. Bockarie MJ, Alexander ND, Kazura JW, Bockarie F, Griffin L, Alpers MP. Treatment with ivermectin reduces the high prevalence of scabies in a village in Papua New Guinea. Acta Tropica. 2000;75(1):127-30.
20. Romani L, Whitfeld MJ, Koroivueta J, Kama M, Wand H, Tikoduadua L, et al. Mass Drug Administration for Scabies Control in a Population with Endemic Disease. New England Journal of Medicine. 2015;373(24):2305-13.
21. Hotez P. Mass Drug Administration and Integrated Control for the World's High-Prevalence Neglected Tropical Diseases. Clinical Pharmacology & Therapeutics. 2009;85(6):659-64.
22. Romani L, Marks M, Sokana O, Nasi T, Kamoriki B, Wand H, et al. Feasibility and safety of mass drug coadministration with azithromycin and ivermectin for the control of neglected tropical diseases: a single-arm intervention trial. The Lancet Global Health. 2018;6(10):e1132-e8.
23. Bernigaud C, Samarawickrama GR, Jones MK, Gasser RB, Fischer K. The Challenge of Developing a Single-Dose Treatment for Scabies. Trends in Parasitology. 2019;35(11):931-43.
24. Report of the Tenth Meeting of the WHO Strategic and Technical Advisory Group for Neglected Tropical Diseases. World Health Organisation; 2017.
25. Rosumeck S, Nast A, Dressler C. Ivermectin and permethrin for treating scabies. Cochrane Database of Systematic Reviews. 2018;4:CD012994.
26. Mohammed KA, Deb RM, Stanton MC, Molyneux DH. Soil transmitted helminths and scabies in Zanzibar, Tanzania following mass drug administration for lymphatic filariasis--a rapid assessment methodology to assess impact. Parasites & Vectors [Electronic Resource]. 2012;5:299.
27. Martin D, Wiegand R, Goodhew B, Lammie P, Mkocha H, Kasubi M. Impact of Ivermectin Mass Drug Administration for Lymphatic Filariasis on Scabies in Eight Villages in Kongwa District, Tanzania. The American Journal of Tropical Medicine and Hygiene. 2018;99(4):937-9.
28. Hodge N, Slatyer B, Skiller L. Solomon Islands Health System Review. Whittaker M, editor. Geneva, Switzerland: World Health Organisation; 2015.
29. Human Development Indices and Indicators: 2018 Statistical Update. Washington DC, USA: United Nations Development Programme; 2018.
30. World Health Organisation Model List of Essential Medicines, 21st List. Geneva: World Health Organisation; 2019.
31. Lawrence J, Sodahlon YK, Ogoussan KT, Hopkins AD. Growth, Challenges, and Solutions over 25 Years of Mectizan and the Impact on Onchocerciasis Control. PLOS Neglected Tropical Diseases. 2015;9(5):e0003507.
32. Alexander ND, Cousens SN, Yahaya H, Abiose A, Jones BR. Ivermectin dose assessment without weighing scales. Bulletin of the World Health Organization. 1993;71(3-4):361-6.
33. Stunkard AJ, Sørensen T, Schulsinger F. Use of the Danish Adoption Register for the study of obesity and thinness. Research publications - Association for Research in Nervous and Mental Disease. 1983;60:115-20.
34. Australian Medicines Handbook [Internet]. Available from: https://amhonline.amh.net.au.acs.hcn.com.au/chapters/dermatological-drugs/scabicides-pediculicides/permethrin.
35. Marks M, Engelman D, Romani L, Mason D, Sokana O, Kama M, et al. Exploration of a simplified clinical examination for scabies to support public health decision-making. PLoS Neglected Tropical Diseases [electronic resource]. 2018;12(12):e0006996.
36. Engelman D, Fuller LC, Steer AC, for the International Alliance for the Control of Scabies Delphi p. Consensus criteria for the diagnosis of scabies: A Delphi study of international experts. PLOS Neglected Tropical Diseases. 2018;12(5):e0006549.
37. Osti MH, Sokana O, Gorae C, Whitfeld MJ, Steer AC, Engelman D. The diagnosis of scabies by non-expert examiners: A study of diagnostic accuracy. PLOS Neglected Tropical Diseases. 2019;13(8):e0007635.
38. Australian Public Assessment Report for Ivermectin. Woden, ACT, Australia: Commonwealth of Australia; 2013.

For peer review only - http://bmjopen.bmj.com/site/about/guidelines.xhtml

39. Guzzo CA, Furtek CI, Porras AG, Chen C, Tipping R, Clineschmidt CM, et al. Safety, Tolerability, and Pharmacokinetics of Escalating High Doses of Ivermectin in Healthy Adult Subjects. The Journal of Clinical Pharmacology. 2002;42(10):1122-33.
40. Ottesen EA, Hooper PJ, Bradley M, Biswas G. The Global Programme to Eliminate Lymphatic Filariasis: Health Impact after 8 Years. PLoS Neglected Tropical Diseases. 2008;2(10):e317.
41. Tielsch JM, Beeche A. Impact of ivermectin on illness and disability associated with onchocerciasis. Tropical Medicine & International Health. 2004;9(4):A45-A56.
42. Gardon J, Gardon-Wendel N, Demanga N, Kamgno J, Chippaux J-P, Boussinesq M. Serious reactions after mass treatment of onchocerciasis with ivermectin in an area endemic for Loa loa infection. The Lancet. 1997;350(9070):18-22.
43. Solomon Islands Essential Medicines List. Honiara, Solomon Islands: Ministry of Health and Medical Services; 2017.
44. Strong M, Johnstone P. Interventions for treating scabies. The Cochrane database of systematic reviews. 2007;2007(3):CD000320-CD.
45. Currie BJ, McCarthy JS. Permethrin and ivermectin for scabies. New England Journal of Medicine. 2010;362(8):717-25.
46. Hoffmann JC, Mossner R, Schon MP, Lippert U. Topical scabies therapy with permethrin is effective and well tolerated in infants younger than two months. J Dtsch Dermatol Ges. 2019;17(6):597-600.
47. Landau S, Stahl D. Sample size and power calculations for medical studies by simulation when closed form expressions are not available. Statistical Methods in Medical Research. 2013;22(3):324-45.
48. Provincial Profile of the 2009 Population & Housing Census: Western, Solomon Islands. 2009.
49. Hayes RJ, Moulton LH. Cluster randomised trials, second edition: CRC Press; 2017. 424 p.
50. Harris PA, Taylor R, Minor BL, Elliott V, Fernandez M, O'Neal L, et al. The REDCap consortium: Building an international community of software platform partners. Journal of Biomedical Informatics. 2019;95:103208.
51. Harris PA, Taylor R, Thielke R, Payne J, Gonzalez N, Conde JG. Research electronic data capture (REDCap)—A metadata-driven methodology and workflow process for providing translational research informatics support. Journal of Biomedical Informatics. 2009;42(2):377-81.
52. CONSORT. CONSORT Transparent Reporting of Trials [updated 2020.]. Available from: http://www.consort-statement.org/.
53. SPIRIT. SPIRIT Statement [updated 2020. Available from: https://www.spirit-statement.org/.
54. Chan A-W, Tetzlaff JM, Altman DG, Laupacis A, Gøtzsche PC, Krleža-Jerić K, et al. SPIRIT 2013 statement: defining standard protocol items for clinical trials. Ann Intern Med. 2013;158(3):200-7.
55. Mounsey KE, Bernigaud C, Chosidow O, McCarthy JS. Prospects for Moxidectin as a New Oral Treatment for Human Scabies. PLoS Neglected Tropical Diseases [electronic resource]. 2016;10(3):e0004389.

For peer review only

Table 1: Case definitions for scabies using the 2018 IACS Criteria

Criteria Category		Used in Survey
Confirmed scabies		
A1	Mites, eggs or faeces on light microscopy of skin samples	No
A2	Mites, eggs or faeces visualised on individual using high-powered imaging device	No
A3	Mite visualised on individual dermoscopy	No
Clinical scabies		
B1	Scabies burrows	No
B2	Typical lesions affecting male genitalia	No
B3	Typical lesions in a typical distribution and two history features*	Yes
Suspected Scabies		
C1	Typical lesions in a typical distribution and one history feature*	Yes
C2	Atypical lesions or atypical distribution and two history features*	Yes

**History features include (i) itch, (ii) close contact with an individual who has itch or typical lesions in a typical distribution*

For peer review only

For peer review only

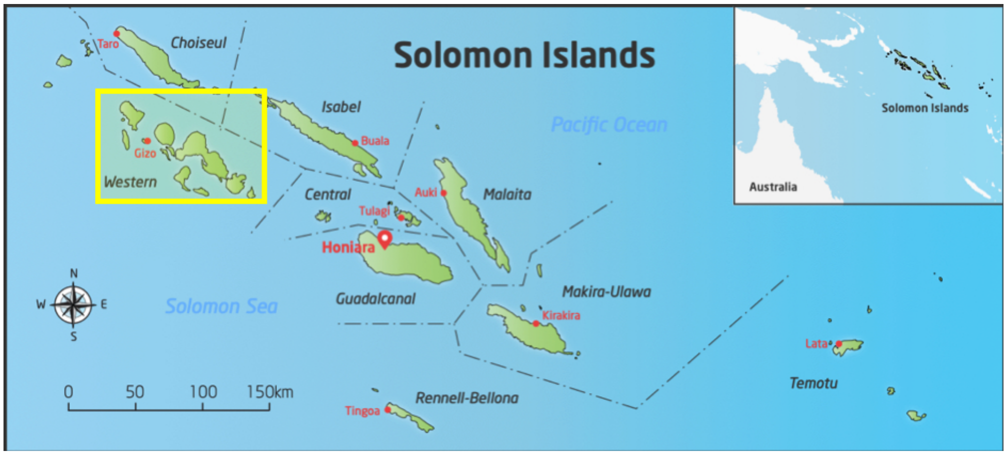

Figure 1: Study location in Solomon Islands

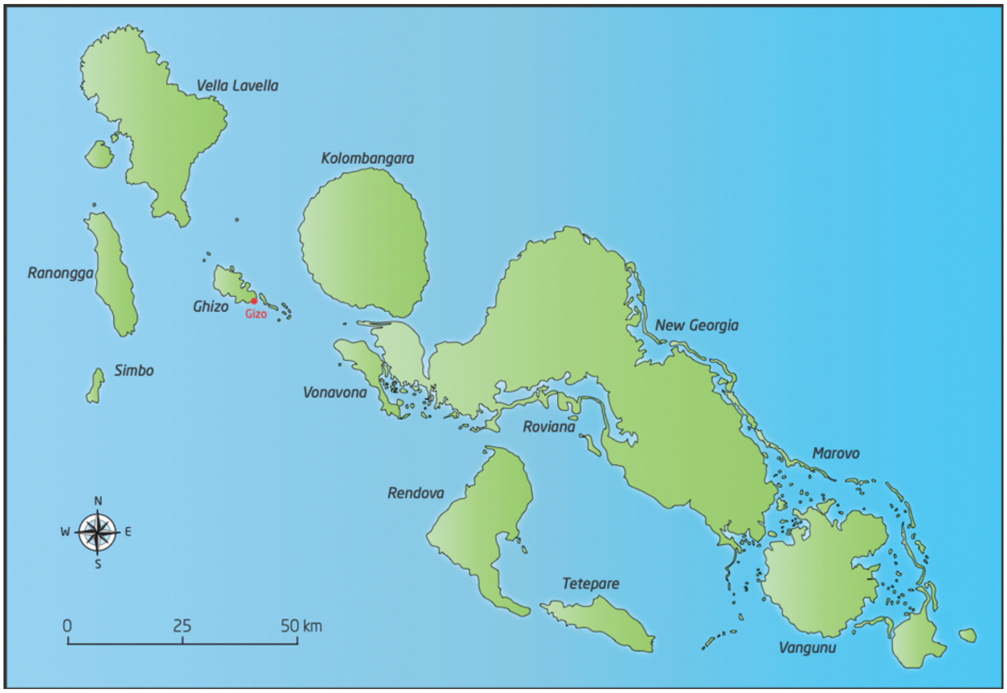

Figure 2: Study location in Western Province, Solomon Islands

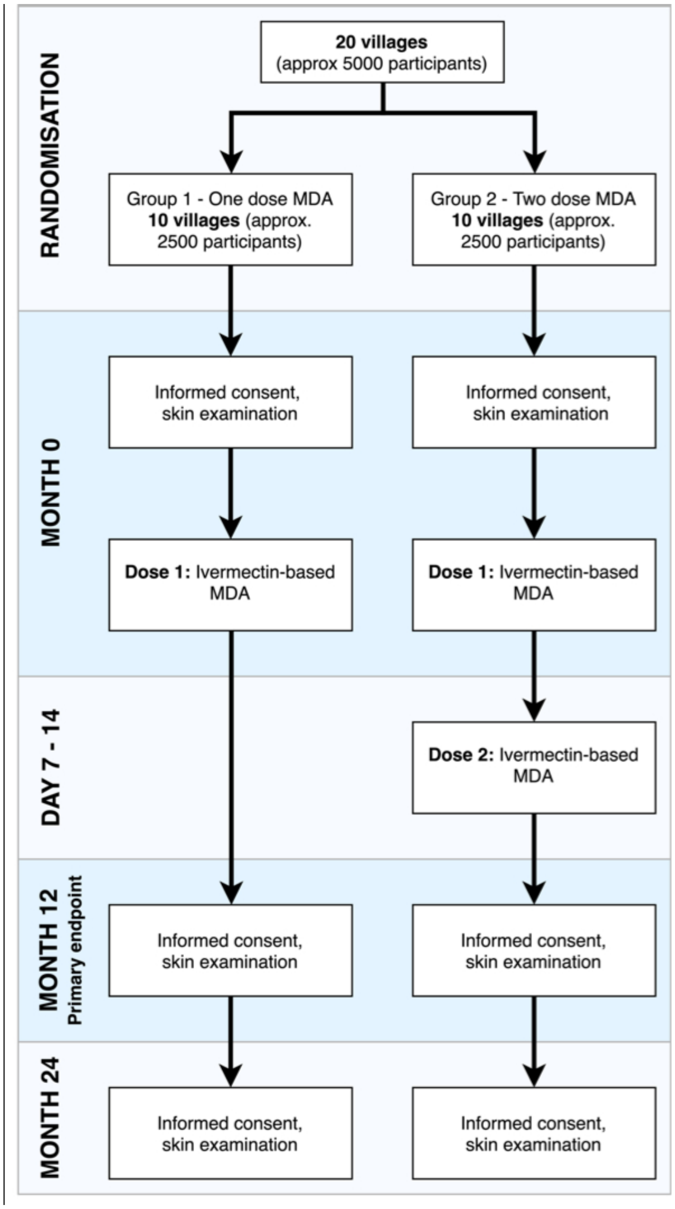

Figure 3: Study flow diagram

Reporting checklist for protocol of a clinical trial.

Based on the SPIRIT guidelines.

Instructions to authors

Complete this checklist by entering the page numbers from your manuscript where readers will find each of the items listed below.

Your article may not currently address all the items on the checklist. Please modify your text to include the missing information. If you are certain that an item does not apply, please write "n/a" and provide a short explanation.

Upload your completed checklist as an extra file when you submit to a journal.

In your methods section, say that you used the SPIRITreporting guidelines, and cite them as:

Chan A-W, Tetzlaff JM, Altman DG, Laupacis A, Gøtzsche PC, Krleža-Jerić K, Hróbjartsson A, Mann H, Dickersin K, Berlin J, Doré C, Parulekar W, Summerskill W, Groves T, Schulz K, Sox H, Rockhold FW, Rennie D, Moher D. SPIRIT 2013 Statement: Defining standard protocol items for clinical trials. Ann Intern Med. 2013;158(3):200-207

		Reporting Item	Page Number
**Administrative information**			
Title	#1	Descriptive title identifying the study design, population, interventions, and, if applicable, trial acronym	1
Trial registration	#2a	Trial identifier and registry name. If not yet registered, name of intended registry	4
Trial registration: data set	#2b	All items from the World Health Organization Trial Registration Data Set	1, 4, 24
Protocol version	#3	Date and version identifier	N/A V4 25/2/19
Funding	#4	Sources and types of financial, material, and other support	24

For peer review only

Roles and responsibilities: contributorship	#5a	Names, affiliations, and roles of protocol contributors	1
Roles and responsibilities: sponsor contact information	#5b	Name and contact information for the trial sponsor	1, 2
Roles and responsibilities: sponsor and funder	#5c	Role of study sponsor and funders, if any, in study design; collection, management, analysis, and interpretation of data; writing of the report; and the decision to submit the report for publication, including whether they will have ultimate authority over any of these activities	24
Roles and responsibilities: committees	#5d	Composition, roles, and responsibilities of the coordinating centre, steering committee, endpoint adjudication committee, data management team, and other individuals or groups overseeing the trial, if applicable (see Item 21a for data monitoring committee)	1, 24-25
**Introduction**			
Background and rationale	#6a	Description of research question and justification for undertaking the trial, including summary of relevant studies (published and unpublished) examining benefits and harms for each intervention	6-8
Background and rationale: choice of comparators	#6b	Explanation for choice of comparators	9
Objectives	#7	Specific objectives or hypotheses	10
Trial design	#8	Description of trial design including type of trial (eg, parallel group, crossover, factorial, single group), allocation ratio, and framework (eg, superiority, equivalence, non-inferiority, exploratory)	9
**Methods: Participants,**			

For peer review only

**interventions, and outcomes**			
Study setting	#9	Description of study settings (eg, community clinic, academic hospital) and list of countries where data will be collected. Reference to where list of study sites can be obtained	11
Eligibility criteria	#10	Inclusion and exclusion criteria for participants. If applicable, eligibility criteria for study centres and individuals who will perform the interventions (eg, surgeons, psychotherapists)	11-12
Interventions: description	#11a	Interventions for each group with sufficient detail to allow replication, including how and when they will be administered	13-14
Interventions: modifications	#11b	Criteria for discontinuing or modifying allocated interventions for a given trial participant (eg, drug dose change in response to harms, participant request, or improving / worsening disease)	16
Interventions: adherance	#11c	Strategies to improve adherence to intervention protocols, and any procedures for monitoring adherence (eg, drug tablet return; laboratory tests)	13-14
Interventions: concomitant care	#11d	Relevant concomitant care and interventions that are permitted or prohibited during the trial	12
Outcomes	#12	Primary, secondary, and other outcomes, including the specific measurement variable (eg, systolic blood pressure), analysis metric (eg, change from baseline, final value, time to event), method of aggregation (eg, median, proportion), and time point for each outcome. Explanation of the clinical relevance of chosen efficacy and harm outcomes is strongly recommended	14-16
Participant timeline	#13	Time schedule of enrolment, interventions (including any run-ins and washouts), assessments, and visits for participants. A schematic diagram is highly recommended (see Figure)	13-15, Fig. 3
Sample size	#14	Estimated number of participants needed to achieve study objectives and how it was determined, including	17-18

For peer review only

		clinical and statistical assumptions supporting any sample size calculations	
Recruitment	#15	Strategies for achieving adequate participant enrolment to reach target sample size	12-13
**Methods: Assignment of interventions (for controlled trials)**			
Allocation: sequence generation	#16a	Method of generating the allocation sequence (eg, computer-generated random numbers), and list of any factors for stratification. To reduce predictability of a random sequence, details of any planned restriction (eg, blocking) should be provided in a separate document that is unavailable to those who enrol participants or assign interventions	18
Allocation concealment mechanism	#16b	Mechanism of implementing the allocation sequence (eg, central telephone; sequentially numbered, opaque, sealed envelopes), describing any steps to conceal the sequence until interventions are assigned	N/A unblinded study
Allocation: implementation	#16c	Who will generate the allocation sequence, who will enrol participants, and who will assign participants to interventions	18
Blinding (masking)	#17a	Who will be blinded after assignment to interventions (eg, trial participants, care providers, outcome assessors, data analysts), and how	9
Blinding (masking): emergency unblinding	#17b	If blinded, circumstances under which unblinding is permissible, and procedure for revealing a participant's allocated intervention during the trial	N/A unblinded study
**Methods: Data collection, management, and analysis**			
Data collection plan	#18a	Plans for assessment and collection of outcome, baseline, and other trial data, including any related processes to promote data quality (eg, duplicate	14-16

For peer review only

		measurements, training of assessors) and a description of study instruments (eg, questionnaires, laboratory tests) along with their reliability and validity, if known. Reference to where data collection forms can be found, if not in the protocol	
Data collection plan: retention	#18b	Plans to promote participant retention and complete follow-up, including list of any outcome data to be collected for participants who discontinue or deviate from intervention protocols	12-13
Data management	#19	Plans for data entry, coding, security, and storage, including any related processes to promote data quality (eg, double data entry; range checks for data values). Reference to where details of data management procedures can be found, if not in the protocol	20
Statistics: outcomes	#20a	Statistical methods for analysing primary and secondary outcomes. Reference to where other details of the statistical analysis plan can be found, if not in the protocol	18-19
Statistics: additional analyses	#20b	Methods for any additional analyses (eg, subgroup and adjusted analyses)	18-19
Statistics: analysis population and missing data	#20c	Definition of analysis population relating to protocol non-adherence (eg, as randomised analysis), and any statistical methods to handle missing data (eg, multiple imputation)	18-19
**Methods: Monitoring**			
Data monitoring: formal committee	#21a	Composition of data monitoring committee (DMC); summary of its role and reporting structure; statement of whether it is independent from the sponsor and competing interests; and reference to where further details about its charter can be found, if not in the protocol. Alternatively, an explanation of why a DMC is not needed	16-17
Data monitoring: interim analysis	#21b	Description of any interim analyses and stopping guidelines, including who will have access to these	N/A Single intervention. DSMB

For peer review only

		interim results and make the final decision to terminate the trial	and Principal Investigator can stop the study.
Harms	#22	Plans for collecting, assessing, reporting, and managing solicited and spontaneously reported adverse events and other unintended effects of trial interventions or trial conduct	16-17
Auditing	#23	Frequency and procedures for auditing trial conduct, if any, and whether the process will be independent from investigators and the sponsor	17
**Ethics and dissemination**			
Research ethics approval	#24	Plans for seeking research ethics committee / institutional review board (REC / IRB) approval	20-21
Protocol amendments	#25	Plans for communicating important protocol modifications (eg, changes to eligibility criteria, outcomes, analyses) to relevant parties (eg, investigators, REC / IRBs, trial participants, trial registries, journals, regulators)	N/A
Consent or assent	#26a	Who will obtain informed consent or assent from potential trial participants or authorised surrogates, and how (see Item 32)	11-13
Consent or assent: ancillary studies	#26b	Additional consent provisions for collection and use of participant data and biological specimens in ancillary studies, if applicable	N/A No biological specimens
Confidentiality	#27	How personal information about potential and enrolled participants will be collected, shared, and maintained in order to protect confidentiality before, during, and after the trial	20
Declaration of interests	#28	Financial and other competing interests for principal investigators for the overall trial and each study site	24
Data access	#29	Statement of who will have access to the final trial dataset, and disclosure of contractual agreements that limit such access for investigators	21

For peer review only

Ancillary and post trial care	#30	Provisions, if any, for ancillary and post-trial care, and for compensation to those who suffer harm from trial participation	N/A If required post-trial care to be delivered through local health system
Dissemination policy: trial results	#31a	Plans for investigators and sponsor to communicate trial results to participants, healthcare professionals, the public, and other relevant groups (eg, via publication, reporting in results databases, or other data sharing arrangements), including any publication restrictions	18
Dissemination policy: authorship	#31b	Authorship eligibility guidelines and any intended use of professional writers	25
Dissemination policy: reproducible research	#31c	Plans, if any, for granting public access to the full protocol, participant-level dataset, and statistical code	21
**Appendices**			
Informed consent materials	#32	Model consent form and other related documentation given to participants and authorised surrogates	N/A
Biological specimens	#33	Plans for collection, laboratory evaluation, and storage of biological specimens for genetic or molecular analysis in the current trial and for future use in ancillary studies, if applicable	N/A No biological specimens to be collected

Notes:

- 3: N/A V4 25/2/19
- 13: 13-15, Fig. 3
- 16b: N/A unblinded study
- 17b: N/A unblinded study
- 21b: N/A Single intervention. DSMB and Principal Investigator can stop the study.
- 26b: N/A No biological specimens
- 30: N/A If required post-trial care to be delivered through local health system
- 33: N/A No biological specimens to be collected The SPIRIT checklist is distributed under the terms of the Creative Commons Attribution License CC-BY-ND 3.0. This checklist was completed on 23. January

For peer review only

2020 using https://www.goodreports.org/, a tool made by the EQUATOR Network in collaboration with Penelope.ai

For peer review only

BMJ Open

Protocol for a cluster randomised non-inferiority trial of one versus two doses of ivermectin for the control of scabies using a mass drug administration strategy (the RISE study)

Journal:	*BMJ Open*
Manuscript ID	bmjopen-2020-037305.R1
Article Type:	Protocol
Date Submitted by the Author:	15-May-2020

Complete List of Authors:	Lake, Susanna; Murdoch Childrens Research Institute, Tropical Disease Research Group; The University of Melbourne, Department of Paediatrics<br>Phelan, Sophie; University of New South Wales, The Kirby Institute<br>Engelman, Daniel; Murdoch Childrens Research Institute, Tropical Disease Research Group; The University of Melbourne, Department of Paediatrics<br>Sokana, Oliver; Ministry of Health and Medical Services<br>Nasi, Titus; Ministry of Health and Medical Services<br>Boara, Dickson; Ministry of Health and Medical Services<br>Gorae, Christina; Ministry of Health and Medical Services<br>Schuster, Tibor; McGill University, Clinical Epidemiology and Biostatistics Unit<br>Grobler, Anneke; Murdoch Childrens Research Institute, Clinical Epidemiology and Biostatistics Unit<br>Osti, Millicent; Murdoch Childrens Research Institute, Tropical Disease Research Group<br>Andrews, Ross; Australian National University<br>Marks, Michael; London School of Hygiene and Tropical Medicine, Clinical Research Department; Hospital for Tropical Diseases,<br>Whitfeld, Margot; St Vincent's Hospital , Department of Dermatology<br>Romani, Lucia; University of New South Wales, The Kirby Institute<br>Kaldor, John; University of New South Wales, The Kirby Institute<br>Steer, Andrew; Murdoch Childrens Research Institute, Tropical Disease Research Group; The University of Melbourne, Department of Paediatrics

**Primary Subject Heading**:	Global health
Secondary Subject Heading:	Dermatology
Keywords:	TROPICAL MEDICINE, INFECTIOUS DISEASES, DERMATOLOGY, International health services < HEALTH SERVICES ADMINISTRATION & MANAGEMENT

I, the Submitting Author has the right to grant and does grant on behalf of all authors of the Work (as defined in the below author licence), an exclusive licence and/or a non-exclusive licence for contributions from authors who are: i) UK Crown employees; ii) where BMJ has agreed a CC-BY licence shall apply, and/or iii) in accordance with the terms applicable for US Federal Government officers or employees acting as part of their official duties; on a worldwide, perpetual, irrevocable, royalty-free basis to BMJ Publishing Group Ltd ("BMJ") its licensees and where the relevant Journal is co-owned by BMJ to the co-owners of the Journal, to publish the Work in this journal and any other BMJ products and to exploit all rights, as set out in our licence.

The Submitting Author accepts and understands that any supply made under these terms is made by BMJ to the Submitting Author unless you are acting as an employee on behalf of your employer or a postgraduate student of an affiliated institution which is paying any applicable article publishing charge ("APC") for Open Access articles. Where the Submitting Author wishes to make the Work available on an Open Access basis (and intends to pay the relevant APC), the terms of reuse of such Open Access shall be governed by a Creative Commons licence – details of these licences and which Creative Commons licence will apply to this Work are set out in our licence referred to above.

Other than as permitted in any relevant BMJ Author's Self Archiving Policies, I confirm this Work has not been accepted for publication elsewhere, is not being considered for publication elsewhere and does not duplicate material already published. I confirm all authors consent to publication of this Work and authorise the granting of this licence.

For peer review only

Protocol for a cluster randomised non-inferiority trial of one versus two doses of ivermectin for the control of scabies using a mass drug administration strategy (the RISE study)

Susanna J. Lake MIPH (1,2,3)

Sophie L. Phelan MIPH MAE (4)

Daniel Engelman PhD (1,2,3)

Oliver Sokana MPH(5)

Titus Nasi MCH(5)

Dickson Boara PgDipHSM (5)

Christina Gorae (5)

Tibor Schuster PhD (6)

Anneke C. Grobler PhD (7)

Millicent H. Osti MBBS (1,2)

Ross Andrews PhD (8)

Michael Marks PhD (9,10)

Margot J. Whitfeld FACD (11)

Lucia Romani PhD (4)

John M. Kaldor PhD (4)

Andrew C. Steer PhD (1,2,3)

1. Tropical Diseases Research Group, Murdoch Children's Research Institute, Melbourne, Australia
2. Department of Paediatrics, University of Melbourne, Melbourne, Australia
3. Melbourne Children's Global Health, Melbourne, Australia
4. Kirby Institute, University of New South Wales, Sydney, Australia

For peer review only

5. Ministry of Health and Medical Services, Solomon Islands
6. McGill University, Montreal Canada
7. Clinical Epidemiology and Biostatistics Unit, Murdoch Children's Research Institute, Melbourne, Australia
8. Australian National University, Canberra, Australia
9. London School of Hygiene and Tropical Medicine, London, United Kingdom
10. Hospital for Tropical Diseases, London, United Kingdom
11. Department of Dermatology, St Vincent's Hospital, Sydney, Australia

Correspondence to:

Professor Andrew C Steer; Andrew.Steer@rch.org.au

Keywords:

Scabies

Mass drug administration

Ivermectin

Solomon Islands

For peer review only

ABSTRACT

Introduction

Scabies is a significant contributor to global morbidity, affecting approximately 200 million people at any time. Scabies is endemic in many resource-limited tropical settings. Bacterial skin infection (impetigo) frequently complicates scabies infestation in these settings. Community-wide ivermectin-based mass drug administration (MDA) is an effective control strategy for scabies in island settings, with a single round of MDA reducing population prevalence by around 90%. However, current two-dose regimens present a number of barriers to programmatic MDA implementation. We designed the Regimens of Ivermectin for Scabies Elimination (RISE) trial to investigate whether one-dose MDA may be as effective as two-dose MDA in controlling scabies in high-prevalence settings.

Methods and analysis

RISE is a cluster randomised non-inferiority trial. The study will be conducted in 20 isolated villages in Western Province of Solomon Islands where population prevalence of scabies is approximately 20%. Villages will be randomly allocated to receive either one dose or two doses of ivermectin-based MDA in a 1:1 ratio. The primary objective of the study is to determine if ivermectin-based MDA with one dose is as effective as MDA with two doses in reducing the prevalence of scabies after 12 months. Secondary objectives include the effect of ivermectin-based MDA on impetigo prevalence after 12 months and 24 months, the prevalence of scabies at 24 months after the intervention, the impact on presentations to health facilities with scabies and impetigo, and the safety of one-dose and two-dose MDA.

For peer review only

Ethics and dissemination

This trial has been approved by the ethics review committees of the Solomon Islands and the Royal Children's Hospital, Australia. Results will be disseminated in peer-reviewed publications and in meetings with the Solomon Islands Ministry of Health and Medical Services and participating communities.

Trial Registration

Australian New Zealand Clinical Trials Registry: ACTRN12618001086257. Date registered: 28 June, 2018.

For peer review only

ARTICLE SUMMARY

Strengths and limitations of this study

- The cluster randomised study design follows the implementation of the intervention at a village level
- Follow up at both 12 and 24 months will demonstrate longer term effects of the intervention
- The sample size (5000 people across 20 villages) achieves a statistical power of 80%
- This study is being conducted in partnership with the Solomon Islands Ministry of Health and Medical Service and will build the capacity of local nursing staff as well as being conducted in a culturally sensitive manner
- Scabies prevalence is high in the isolated island villages where this study will be conducted (approximately 20%), therefore results may not be transferable to lower prevalence or urban settings.

For peer review only

BACKGROUND

Scabies is a neglected tropical disease (NTD) caused by infestation with the mite *Sarcoptes scabiei* var. *hominis*. Scabies is a significant contributor to global morbidity, estimated to cause 455 million annual incident cases.(1, 2) Transmission occurs primarily as a result of skin-to-skin contact (and rarely due to fomites) and is more common in overcrowded settings, including in many tropical environments where crowding and poverty are prevalent and access to treatment limited.(3, 4) The burden of disease is substantial in many Pacific Island Countries where scabies affects 1 in 5 people and up to 1 in 2 children.(5)

Scabies infestation causes intense itch and discomfort. Furthermore, it is responsible for a considerable proportion of bacterial skin infection (impetigo) in many resource-limited settings.(6-8) Scabies causes a breach in the skin barrier from scabetic lesions and subsequent scratching, creating an entry point for bacteria including *Staphylococcus aureus* and *Streptococcus pyogenes.* Resulting impetigo can in turn cause severe infection and immune-mediated disease including sepsis, glomerulonephritis and possibly rheumatic fever.(9-12)

Treatment guidelines for scabies recommend treatment of the infected individual as well as household contacts.(13, 14) Most guidelines recommend treatment with topical acaracides such as permethrin or benzoyl benzoate.(13, 14) These medications are effective, if applied to all affected areas for the correct duration, but re-infestation frequently occurs in highly endemic settings where individuals may be exposed to infected household or community members, many of whom may be asymptomatic.(15) Therefore, attention has shifted to simultaneous treatment of whole communities, including those without symptoms of infestation, to reduce prevalence and the rate of transmission. (16) This strategy of mass drug

For peer review only

administration (MDA) has been used to successfully control a number of NTDs including onchocerciasis, lymphatic filariasis (LF), trachoma and soil-transmitted helminths and there is a growing body of evidence to support MDA for scabies control.(17-22)

Ivermectin is an antiparasitic drug in the avermectin class that is active against the scabies mite. Ivermectin-based MDA for scabies involves offering ivermectin treatment to the whole community, with the exception of young children, pregnant women and others with a contraindication to ivermectin. Permethrin cream is offered as an alternative to ivermectin for these groups. Several studies in Pacific Island Countries with high-prevalence have shown ivermectin-based MDA can reduce the population prevalence of scabies by around 90%.(7, 17, 23) The SHIFT study in Fiji was the first comparative study to demonstrate the effectiveness of ivermectin MDA for scabies control, finding a reduction in the population prevalence of scabies from 32% at baseline to less than 2% at 12 months.(21) These trials have all used an MDA strategy involving two doses of medication, given 7-14 days apart (either to the whole community, or those with clinical signs of scabies).(7, 23) This is consistent with clinical recommendations for treatment of individuals.(13) Ivermectin is known to lack ovicidal activity, therefore the second dose aims to kill newly hatched mites.(24)

In 2017, the World Health Organization (WHO) recognised scabies as a NTD, and identified the need for public health action to control scabies in endemic settings.(9) The Strategic and Technical Advisory Group on Neglected Tropical Diseases called for further research into control strategies for scabies and the development of guidelines for the public health use of avermectins.(25)

For peer review only - http://bmjopen.bmj.com/site/about/guidelines.xhtml

While ivermectin-based MDA shows great promise as a control strategy for scabies, the requirement for two doses of medication at each MDA round presents barriers to implementation. Drug and implementation costs are doubled compared to single-dose MDA. Distribution is more complex and integration with programs for other NTDs is difficult. These challenges are amplified in remote island settings where the population is dispersed across difficult to reach villages and funding for programs is limited. These hurdles may be prohibitive to wide-spread implementation of scabies control, particularly in low-income settings. Therefore, the optimum dosing strategy for MDA remains an important knowledge gap.(9) For this reason we designed the Regimens of Ivermectin for Scabies Elimination (RISE) trial.

For peer review only

METHODS AND ANALYSIS

Study Design

The RISE trial is a prospective, open-label comparison of one dose versus two doses of ivermectin-based MDA for the population-level control of scabies. Using a cluster-randomised design, 20 villages will be randomised to one of two intervention groups in a 1:1 ratio. Randomisation will occur at the village level, rather than the individual level, as the objective is to determine the dosing regimen for controlling scabies within whole communities. Randomisation minimises the possibility of the anticipated difference in the outcome between each group being confounded. A two-dose regimen is an appropriate comparator (rather than no-treatment or placebo) as two-doses of ivermectin-based MDA is the currently accepted dosing regimen. We chose a non-inferiority design because it is unlikely that a one-dose regimen would be superior in effectiveness to a two-dose regimen. However, the logistic and pragmatic advantages of a one-dose regimen compared to a two-dose regimen make a non-inferior study appealing.

The prevalence of scabies and impetigo will be measured before the intervention (baseline), and repeated at 12 months and 24 months after the intervention. To measure a secondary outcome the standard Solomon Islands Ministry of Health and Medical Services (MHMS) health facility reporting processes will be used to capture the number of presentations to health facilities with scabies and impetigo in the study catchment area for the 12 month period before the intervention and for the 24 month period after the intervention.

For peer review only

Aims

The primary objective of this study is to determine if ivermectin-based MDA with one dose is non-inferior to two doses in reducing the prevalence of scabies 12 months after the intervention. The secondary objectives are to assess the impact of ivermectin-based MDA on: population prevalence of scabies after 24 months; population prevalence of impetigo after 12 months and 24 months; the number of presentations to health clinics with scabies and impetigo before and after the intervention; the number of adverse events measured by passive surveillance in the 12-months after MDA in each study group. The trial will also be measuring outcomes related to the impact of ivermectin MDA on the prevalence and intensity of soil transmitted helminths however this paper focuses on scabies and impetigo outcomes.

Rationale

This study uses ivermectin-based MDA because the SHIFT trial in Fiji demonstrated the greatest reduction in scabies prevalence after ivermectin-based MDA.(21) Ivermectin is currently the only oral therapy available for scabies and it allows greater compliance than with topical therapy. Oral therapy can be directly observed, ensuring adherence to treatment. Although ivermectin only kills the mature scabies mite and not the eggs, a single dose of treatment simultaneously administered to a whole village may reduce transmission sufficiently to reduce population prevalence. A recent Cochrane review did not find a difference in efficacy of one dose of oral ivermectin compared to two doses of oral ivermectin, but confidence in the effect estimates was low to moderate, with poor reporting a major limitation.(26) A retrospective study in Zanzibar of six rounds of annual single dose

For peer review only

ivermectin MDA for LF showed a 68-98% decline in clinical presentations and treatments for scabies, suggesting a one-dose strategy may significantly reduce transmission.(27) By contrast, annual ivermectin MDA for LF did not reduce the prevalence of scabies in Tanzania where the baseline prevalence was less than 5%.(28)

Study setting and participants

The study will be conducted in Western Province of Solomon Islands (Figures 1 and 2). Solomon Islands is a nation in the South Pacific with a population of over 650,000 spread across 900 islands, a geography that presents many challenges for health service delivery.(29) Solomon Islands is classified as a least developed country. It is ranked 152 out of 189 on the Human Development Index.(30) The majority of the population depend on subsistence agriculture in rural locations. We chose Western Province for this study for several reasons: first, there is a high burden of scabies - a 2014 survey estimated an all-age scabies prevalence of 19.2%;(6) second, we expect that there will be relatively little mixing between villages because of the island geography of isolated villages with no road transport; third, there are many villages of appropriate population size (between 180 and 300) for the cluster-randomised design.

Twenty villages will be selected after close consultation with the MHMS. Criteria for selecting the villages include a population of between 180 and 300 people, geographic isolation and willingness to participate in the study. All residents of the 20 selected villages will be eligible to participate. If a resident of a two-dose village does not take the first dose of medication they will still be eligible to take a dose when the team returns to the village for the second dose.

For peer review only

This is a community-based study, with analysis conducted at the village level, and therefore all residents are eligible to participate in the follow-up assessment at 12 months and 24 months, regardless of whether they received treatment at baseline. Written informed consent will be obtained from all participants. Participants under the age 18 years will require written consent to be provided by a parent or guardian. Consent will be obtained at each study timepoint (baseline, 12 months and 24 months).

Inclusion and exclusion criteria

Inclusion criteria. All participants who provide consent are eligible to participate. If exclusion criteria for treatment are met, then consented participants are still eligible to have their skin examined.

Exclusion criteria. Participants who meet any of the following criteria will not receive treatment, but will be eligible to enrol in the study and undergo skin examination: allergy to ivermectin or permethrin; treatment within the last 7 days with ivermectin or permethrin; declines treatment. Participants with severe acute or chronic illness will not be treated with ivermectin but may receive permethrin.

Patient and public involvement statement

The trial was designed in close consultation with stakeholders at the Solomon Islands MHMS to ensure it was culturally appropriate for the local setting. Staff from the Solomon Islands MHMS contributed to study design and identification of study sites. The study team will

For peer review only - http://bmjopen.bmj.com/site/about/guidelines.xhtml

comprise a majority of Solomon Islander staff from Western Province. Staff are able to communicate in the local regional languages.

A team of health promotion officers from the Solomon Islands MHMS will conduct community awareness in each village, approximately one month prior to MDA. An illustrated information leaflet outlining the study design as well as information about scabies and the treatments will be provided during community visits. A participant information statement that contains contact details for the principal investigator and local investigator will be made available to all village residents. Community awareness and the informed consent process will be conducted in Solomon Islands Pijin and study staff who speak the local regional language will be available to provide further information or clarification as required. Results of the study will be communicated to community leaders and members by the study team.

Intervention

Oral ivermectin will be offered to all participants, unless there is a contraindication to ivermectin. The contraindications for ivermectin are: pregnancy; breastfeeding an infant less than seven days old; age less than two years; height less than 90 cm; concurrent medication that may interact with ivermectin (for example, warfarin); or severe acute or chronic illness on the day of MDA. If ivermectin is contraindicated, then topical permethrin will be offered.

A dose of 200 µg/kg of ivermectin is recommended for the treatment of individuals with scabies.(13, 31) We will aim to dose ivermectin within a range of 150-250 µg/kg, as this dose has been effective in previous trials.(21) We will use 6 mg scored tablets. Doses will be rounded to the nearest 3 mg. The tablets will be accurately halved on the score line using a

For peer review only - http://bmjopen.bmj.com/site/about/guidelines.xhtml

pill cutter as required. As weight scales are generally unsuitable for implementation of MDA, we will use dosing strategies appropriate for larger scale programmatic roll-out.

We will use height-based dosing for children aged less than 15 years, with doses ranging from 3 mg to 12 mg, as is standard for MDA for onchocerciasis and lymphatic filariasis.(32, 33) Adults will receive a standard dose of 12 mg, with doses adjusted based on visual assessment of body shape (9 mg for adults assessed to be a malnourished, 15 mg for adults assessed to be obese). Drug distribution staff will make these assessments based on a series of body shape silhouettes.(34) Dosing of medications for MDA based on physical appearance has been shown to be accurate and safe.(33) Staff will undergo training and validation for these dosing techniques. Ivermectin will be administered by trained study staff who will directly observe swallowing of the tablets.

Topical permethrin 5% cream will be given to participants meeting exclusion criteria for ivermectin. Permethrin will be dosed according to clinical guidelines.(35) Participants or carers will be counselled to apply the cream to the whole body from the neck down (in infants cream should also be applied to the scalp) and to leave it for 8 to 14 hours, or 4 hours in infants less than 2 months of age.

Outcome measures

All participants will undergo assessment for symptoms and signs of scabies, impetigo and other skin disease.(36) Assessment will include history questions regarding the presence of itch, contact history and a simplified skin examination. Skin examination will be limited to areas that are usually exposed (arms from above elbow to fingers, legs from above knee to

For peer review only - http://bmjopen.bmj.com/site/about/guidelines.xhtml

toes, head and neck). Other areas (including breasts, groin or genitals) will not be examined. Data suggest that in this setting a limited examination detects more than 90% cases of scabies.(36) The skin of children less than 2 years age will be examined more generally, as scabies may be more widespread in this age group.(3)

Scabies will be diagnosed according to consensus criteria established by the International Alliance for the Control of Scabies (2020 IACS criteria).(37, 38) Categorisation will be based on the identification of typical scabies lesions, typical body distribution of lesions and presence of itch and/or positive contact history. Diagnosis will therefore use levels B (Clinical Scabies) and C (Suspected Scabies) (See Table 1). Microscopy was not used as it is not feasible or practical for programmatic roll-out in these remote settings. Confirmation of diagnosis with dermatoscopy was not considered feasible as the specialist skills required exceeded the training of the local health workers.

Impetigo will be recorded if papular, pustular or ulcerative lesions surrounded by erythema, or with crusts, pus or bullae are seen.(7) This approach is consistent to diagnostic processes in previous scabies community intervention trials. Examinations will be conducted by nurses from Western Province. Nurses will receive one week of theoretical and practical training in the clinical assessment for scabies and impetigo, including application of the 2020 IACS criteria.(Supplementary file) (39) Nurses will receive additional training prior to the follow-up surveys at 12 and 24 months.

Other severe skin infections such as ulcers, abscesses or suspected cases of crusted scabies will also be recorded where noted. If these, or other significant medical conditions are noted

For peer review only

during the survey, participants will be referred off-study to the local health clinic for assessment and management.

In addition to skin examination data we will also collect information on presentations to health facilities in the study villages. Government health facilities in Solomon Islands routinely record the details of all attendances and admissions in paper-based registers. Cases of scabies, local and serious bacterial infections and other skin diseases are recorded. Facilities report aggregated data using a standardised form each month. Data is transferred electronically through the District Health Information System (DHIS2) to the MHMS Health Information Statistics Unit. We will use the information from DHIS2 to assess the number of presentations to health facilities with scabies and impetigo. Data collected in the 12 months prior to MDA will be compared to data collected in the 24 months following MDA.

Safety monitoring and reporting

Ivermectin is well tolerated and has a significant dose safety margin with no safety concerns at much higher doses than clinically required (up to 120 mg in adults, approximately 2000 μg/kg).(40, 41) Over one billion doses have been distributed for control of onchocerciasis and lymphatic filariasis with few effects reported beyond minor, reversible events.(42, 43) There have been cases of encephalopathy following ivermectin administration but these have been in the context of loiasis, a disease which has not been detected in Solomon Islands.(44) Ivermectin is on the WHO Model Essential Medicines List and the Solomon Islands Essential Medicines List for the treatment of scabies.(31, 45) Although topical benzyl benzoate is the standard treatment of scabies in the Solomon Islands, topical 5% permethrin will be used in the study due to its increased efficacy and lower rate of local side effects.(46) Permethrin is

For peer review only - http://bmjopen.bmj.com/site/about/guidelines.xhtml

well tolerated with very few side effects, including in infants.(47, 48) Nonetheless, we will record all reported adverse events related to treatment using passive monitoring.

Participants will be advised to report any adverse events to clinic nurses or directly to the study team if the adverse event occurs immediately post MDA. The clinic nurses will relay information to the study coordinator who will document the adverse event and send a report to the Principal Investigator who will in turn collate adverse events for reporting to the Data Safety Monitoring Board (DSMB). Any serious adverse events or suspected serious adverse reactions will be reported to the study coordinator by the study team, or clinical staff at the clinics and hospitals in the study area. Hospitals will be briefed on the study and provided with comprehensive reporting forms and the MDA schedule. Hospitals will report any admissions or deaths from study villages for one month following administration of the first dose of ivermectin. We will retrospectively review mortality records to ensure all deaths from study villages have been captured. We will review routinely collected summary data on all stillbirths from hospitals in the area for 12 months following MDA.

An independent DSMB will provide oversight to the safety and progress of the trial. The DSMB will meet via teleconference prior to the study, in the first three months after MDA and at the conclusion of the study. Any serious adverse events and suspected serious adverse reactions will be reported to the DSMB within seven days.

Sample size

Sample size calculations were based on scabies prevalence in Western Province of Solomon Islands and the effect size measured in previous studies of ivermectin-based MDA for

For peer review only

scabies.(6, 17, 21) A standard Monte Carlo simulation method with 1000 repetitions was used to estimate the required sample size and number of villages to achieve statistical power of 80%.(49) We assumed that scabies prevalence across villages would range from 10% to 30% (mean 20%, standard deviation, SD, 5%) at baseline (6). The effect size measured in previous studies with two doses of ivermectin MDA was used to assume the prevalence of scabies 12 months after MDA will be between 3% and 9% (mean 6%, SD 2%) in the one-dose group and between 1% and 5% (mean 3%, SD 1%) in the two-dose group (17, 21). We assumed an average village size of 250 people with a range of 200 to 300.(50) We considered a non-inferiority margin of 5% (prevalence of scabies at 12 months in the one-dose group minus prevalence at 12 months in the two-dose group) to be relevant from a public health perspective. Based on these assumptions, 20 villages, randomised equally, would be sufficient to achieve the required power.

Randomisation

An independent statistician will randomise villages to the one-dose or two-dose group in a 1:1 ratio once the 20 study villages have agreed to participate. There will be no stratification within the randomisation process. There will be 10 villages in each group (Figure 3).

For peer review only - http://bmjopen.bmj.com/site/about/guidelines.xhtml

Table 1: Case definitions for scabies using the 2020 IACS Criteria (38)

Criteria Category		Used in Survey
**Confirmed scabies**		
	At least one of:	
A1	Mites, eggs or faeces on light microscopy of skin samples	No
A2	Mites, eggs or faeces visualised on an individual using a high-powered imaging device	No
A3	Mite visualised on an individual using dermoscopy	No
**Clinical scabies**		
	At least one of:	
B1	Scabies burrows	No
B2	Typical lesions affecting male genitalia	No
B3	Typical lesions in a typical distribution and two history features*	Yes
Suspected Scabies		
	One of:	
C1	Typical lesions in a typical distribution and one history feature*	Yes
C2	Atypical lesions or atypical distribution and two history features*	Yes

**History features include (i) Itch, (ii) Positive contact history*

Diagnosis can be made at one of the three levels (A, B or C). A diagnosis of clinical or suspected scabies should only be made if other differential diagnoses are considered less likely than scabies.

For peer review only

Analysis plan

We will account for clustering when calculating all study outcomes by calculating outcomes at the cluster level and analysing data at the cluster level and not the individual level.(51) The range of cluster-level outcomes will be reported by group.

Primary outcome

The prevalence of scabies in each village will be calculated at baseline (0 months), and 12 months. The prevalence will be calculated by dividing the number of participants with

scabies by the denominator (the total number of participants examined for scabies) in each cluster. The denominator will vary at each timepoint as we will include all participants who consented for skin examination, regardless of their involvement at other timepoints.

The difference in scabies prevalence between baseline and 12 months will be calculated for each village. The means of these differences will be calculated in the two treatment groups and compared by calculating the difference between the means. If the upper limit of the two-sided 95% confidence interval of the mean difference between the two study groups is less than or equal to 5% (the clinically relevant non-inferiority margin) the one-dose regimen will be considered non-inferior.

Secondary outcomes

The analysis for the prevalence of scabies at 24 months and impetigo at 12 and 24 months will be done in the same way as for the primary endpoint.

The change in the number of presentations to health facilities for scabies and impetigo will be analysed in three ways. First, the total number of presentations in the 12 months before MDA will be compared to the number of presentations in months 1 to 12 and 13 to 24 after MDA. Second, we will calculate the proportion of clinic presentations for scabies and impetigo by dividing the number of presenations for scabies and impetigo by the total number of clinic presentations for any condition. We will calculate this proportion for the 12 months before MDA, 1 to 12, and 13 to 24 months after MDA. Calculating the proportion will account for any changes in population size or operational status of health facilities. Third, we will compare the number of clinic presentations for scabies and impetigo in the clinics that service

For peer review only - http://bmjopen.bmj.com/site/about/guidelines.xhtml

the study villages and compare this with clinic presentation for scabies and impetigo in other clinics in the province, this will be adjusted for population size.

The number of adverse events in each study group will be calculated as a proportion of the total number of participants in each study group that received MDA at baseline. We will also report the number of deaths in the month following MDA in each study group as a proportion of the number of participants in each group.

Data collection and management

Data will be collected using a combination of paper-based and electronic forms. Paper forms will be stored in locked filing cabinets. Information will be deidentified and participant names will only be recorded on consent forms. Only authorised study staff will be able to access forms. Data will be destroyed after 15 years in compliance with local guidelines. Skin examination data will be collected and managed using REDCap electronic data capture tools hosted at Murdoch Children's Research Institute.(52, 53) REDCap is a secure, web-based software platform designed to support data capture for research studies.

Trial Status

Baseline data collection and MDA took place between May and July 2019. A total of 5,260 participants were enrolled. Follow-up village data collection is scheduled to take place between May and July 2020 and between May and July 2021.

For peer review only

Ethics and dissemination

The RISE trial is investigator-initiated and funded by the National Health and Medical Research Council of Australia (GNT1127297). The funder was not involved in protocol development or the study process including site selection, management, data collection or analysis of the results. The trial is a collaboration between the Murdoch Children's Research Institute, the Solomon Islands MHMS, the Kirby Institute at the University of New South Wales, the London School of Hygiene and Tropical Medicine and the Australian National University.

The trial was designed in accordance with CONSORT guidelines and our reporting of the protocol conforms to the Standard Protocol Items: Recommendations for Interventional Trials (SPIRIT) 2013 checklist.(54-56) This trial has been approved by the Solomon Islands Health Research and Ethics Review Board (HRE005/18) and Royal Children's Hospital Human Research Ethics Committee (38099A) in Melbourne, Australia and will be conducted in accordance with Good Clinical Practice.

De-identified data may be made available for further analysis with appropriate approvals. Results of the study will be presented locally and made available to health policy decision makers and clinical staff. Villages that participated in the trial will be presented the results in a culturally appropriate way that is easy to understand and interpret. Participants will have the opportunity for results to be explained to them in their own language through a series of village meetings as well as printed information leaflets.

For peer review only - http://bmjopen.bmj.com/site/about/guidelines.xhtml

DISCUSSION

Scabies is a common disease in many tropical and low-income settings and has been prioritised for control by WHO, but there are still gaps in knowledge to determine the optimum approach to control in settings where scabies is highly endemic.(9) The results of this trial will have an impact on national, regional and global strategies for scabies control.

Island communities in the Pacific have among the highest global prevalence of scabies and understanding how to implement MDA in these settings has the potential for translation into huge public health impact for these communities.(5) However, the results may not be generalisable to populations with a much lower prevalence of the disease, to settings with higher population density, or to urban settings. The non-inferiority margin of 5% was determined using available evidence but may not represent the appropriate level of public health significance in all circumstances. A greater or lesser margin may be considered non-inferior in other settings, depending on factors including baseline disease prevalence, number of rounds planned, costs of implementing each regimen and available resources. This trial is designed to assess a single round of MDA, there is scope for further research to assess the efficacy of repeated annual rounds of MDA. The cluster-randomised design will allow analysis of the impact of MDA at the community level. We will be able to assess the impact of the intervention on the whole community, even for those who will not receive MDA.

If the RISE trial finds that one-dose ivermectin MDA is inferior, then the need for two doses of ivermectin-based MDA would need to be taken into account in decision-making around control strategies for scabies. It would also provide impetus for further research to identify new treatments for scabies that may be able to be implemented with one dose. Approaches

For peer review only

may include novel treatments that are ovicidal, or other medications with a longer half-life, such as moxidectin(57). If one-dose ivermectin-based MDA is found to be non-inferior to two-dose then this strategy will be highly attractive for implementation as a public health program. The lower cost, simplified logistics and ability to integrate with other programs would make scabies control programs more feasible in low-income settings.

For peer review only

Funding

This research is funded by the National Health and Medical Research Council of Australia (NHMRC). DE, LR, JK and ACS are supported by fellowships from the NHMRC. MM is supported by the UK National Insitute for Health Research.

Acknowledgements

The authors are grateful to the Solomon Islands MHMS and the communities who will participate. We appreciate the support and contributions of Pauline McNeil, Nemia Bainivalu, Gregory Jilini, Michael Larui, Ivan Ghemu, Freda Pitakaka, William Horoto, Jeffrey Korini, Soraya Pina, Yvonne Tuni, Selina Maena, Frederick Neqo, Susana Vaz Nery and Naomi Clarke. The RISE study team includes: Sana Bisili, Aisling Byrne, Sharmillah Jack, Arthur Keremama, Alam Khatak, Erica Lazu, Relinta Manaka, Davis Pesala, Deanne Seppy, Winter Sino, Patson Solomon, Stephen Tiazi, and Salote Wickham.

This study is dedicated to Dr Tenneth Dalipanda, former Permanent Secretary of the Solomon Islands MHMS. Dr Tenneth was committed to improving the health of Solomon Islanders. He was an advocate for public health research and without his support we would not have been able to conduct this research and many studies before it.

For peer review only

Author contributions

Study concept and design were conducted by the investigators: ACS, DE, JMK, LR, MJW, MM, RA, TS, OS and TN. Critical revision of concept and design and intellectual input in the study protocol was done by all authors: SJL, SLP, DE, OS, TN, DB, CG, TS, ACG, MHO, RA, MM, MJW, LR, JK and ACS. Drafting of the protocol was done by SLP and ACS, with review by all authors. Drafting of the manuscript was done by SJL. Critical revision of the manuscript was performed by all authors. Study supervision is conducted by the investigators.

For peer review only

Competing interests

None declared

Word count:

4429

REFERENCES

1. Vos T, Abajobir AA, Abate KH, Abbafati C, Abbas KM, Abd-Allah F, et al. Global, regional, and national incidence, prevalence, and years lived with disability for 328 diseases and injuries for 195 countries, 1990–2016: a systematic analysis for the Global Burden of Disease Study 2016. The Lancet. 2017;390(10100):1211-59.
2. Karimkhani C, Colombara DV, Drucker AM, Norton SA, Hay R, Engelman D, et al. The global burden of scabies: a cross-sectional analysis from the Global Burden of Disease Study 2015. The Lancet Infectious Diseases. 2017;17(12):1247-54.
3. Hardy M, Engelman D, Steer A. Scabies: A clinical update. Australian Family Physician. 2017;46(5):264-8.
4. Mellanby K. Transmission of Scabies. BMJ. 1941;2(4211):405-6.
5. Romani L, Steer AC, Whitfeld MJ, Kaldor JM. Prevalence of scabies and impetigo worldwide: a systematic review. Lancet Infect Dis. 2015;15(8):960-7.
6. Mason DS, Marks M, Sokana O, Solomon AW, Mabey DC, Romani L, et al. The Prevalence of Scabies and Impetigo in the Solomon Islands: A Population-Based Survey. PLoS Neglected Tropical Diseases [electronic resource]. 2016;10(6):e0004803.
7. Romani L, Koroivueta J, Steer AC, Kama M, Kaldor JM, Wand H, et al. Scabies and impetigo prevalence and risk factors in Fiji: a national survey. PLoS Negl Trop Dis. 2015;9(3):e0003452.
8. Aung PTZ, Cuningham W, Hwang K, Andrews RM, Carapetis JR, Kearns T, et al. Scabies and risk of skin sores in remote Australian Aboriginal communities: A self-controlled case series study. PLoS Negl Trop Dis. 2018;12(7):e0006668.
9. Engelman D, Cantey PT, Marks M, Solomon AW, Chang AY, Chosidow O, et al. The public health control of scabies: priorities for research and action. Lancet. 2019;394(10192):81-92.
10. Parks T, Smeesters PR, Steer AC. Streptococcal skin infection and rheumatic heart disease. Curr Opin Infect Dis. 2012;25(2):145-53.
11. Thornley S, Marshall R, Jarrett P, Sundborn G, Reynolds E, Schofield G. Scabies is strongly associated with acute rheumatic fever in a cohort study of Auckland children. J Paediatr Child Health. 2018;54(6):625-32.

For peer review only

12. Tasani M, Tong SY, Andrews RM, Holt DC, Currie BJ, Carapetis JR, et al. The Importance of Scabies Coinfection in the Treatment Considerations for Impetigo. Pediatr Infect Dis J. 2016;35(4):374-8.

13. eTG complete. [Internet]. Melbourne, Victoria: Therapeutic Guidelines Ltd. Available from: https://tgldcdp.tg.org.au.acs.hcn.com.au/viewTopic?topicfile=insects-mites&guidelineName=Dermatology#toc_d1e110.

14. WHO Model Formulary for Children. Geneva, Switzerland: World Health Organisation; 2010.

15. David, Lucinda. A Review of Scabies: An Infestation More than Skin Deep. Dermatology. 2019;235(2):79-90.

16. Engelman D, Steer A. Control Strategies for Scabies. Tropical Medicine and Infectious Disease. 2018;3(3):98.

17. Romani L, Marks M, Sokana O, Nasi T, Kamoriki B, Cordell B, et al. Efficacy of mass drug administration with ivermectin for control of scabies and impetigo, with coadministration of azithromycin: a single-arm community intervention trial. The Lancet Infectious Diseases. 2019;19(5):510-8.

18. Marks M, Taotao-Wini B, Satorara L, Engelman D, Nasi T, Mabey DC, et al. Long term control of scabies fifteen years after an intensive treatment programme. PLoS Negl Trop Dis. 2015;9(12):e0004246.

19. Taplin D, Porcelain SL, Meinking TL, Athey RL, Chen JA, Castillero PM, et al. Community control of scabies: a model based on use of permethrin cream. Lancet. 1991;337(8748):1016-8.

20. Bockarie MJ, Alexander ND, Kazura JW, Bockarie F, Griffin L, Alpers MP. Treatment with ivermectin reduces the high prevalence of scabies in a village in Papua New Guinea. Acta Tropica. 2000;75(1):127-30.

21. Romani L, Whitfeld MJ, Koroivueta J, Kama M, Wand H, Tikoduadua L, et al. Mass Drug Administration for Scabies Control in a Population with Endemic Disease. New England Journal of Medicine. 2015;373(24):2305-13.

22. Hotez P. Mass Drug Administration and Integrated Control for the World's High-Prevalence Neglected Tropical Diseases. Clinical Pharmacology & Therapeutics. 2009;85(6):659-64.

23. Romani L, Marks M, Sokana O, Nasi T, Kamoriki B, Wand H, et al. Feasibility and safety of mass drug coadministration with azithromycin and ivermectin for the control of

For peer review only

neglected tropical diseases: a single-arm intervention trial. The Lancet Global Health. 2018;6(10):e1132-e8.

24. Bernigaud C, Samarawickrama GR, Jones MK, Gasser RB, Fischer K. The Challenge of Developing a Single-Dose Treatment for Scabies. Trends in Parasitology. 2019;35(11):931-43.

25. Report of the Tenth Meeting of the WHO Strategic and Technical Advisory Group for Neglected Tropical Diseases. World Health Organisation; 2017.

26. Rosumeck S, Nast A, Dressler C. Ivermectin and permethrin for treating scabies. Cochrane Database of Systematic Reviews. 2018;4:CD012994.

27. Mohammed KA, Deb RM, Stanton MC, Molyneux DH. Soil transmitted helminths and scabies in Zanzibar, Tanzania following mass drug administration for lymphatic filariasis--a rapid assessment methodology to assess impact. Parasites & Vectors [Electronic Resource]. 2012;5:299.

28. Martin D, Wiegand R, Goodhew B, Lammie P, Mkocha H, Kasubi M. Impact of Ivermectin Mass Drug Administration for Lymphatic Filariasis on Scabies in Eight Villages in Kongwa District, Tanzania. The American Journal of Tropical Medicine and Hygiene. 2018;99(4):937-9.

29. Hodge N, Slatyer B, Skiller L. Solomon Islands Health System Review. Whittaker M, editor. Geneva, Switzerland: World Health Organisation; 2015.

30. Human Development Indices and Indicators: 2018 Statistical Update. Washington DC, USA: United Nations Development Programme; 2018.

31. World Health Organisation Model List of Essential Medicines, 21st List. Geneva: World Health Organisation; 2019.

32. Lawrence J, Sodahlon YK, Ogoussan KT, Hopkins AD. Growth, Challenges, and Solutions over 25 Years of Mectizan and the Impact on Onchocerciasis Control. PLOS Neglected Tropical Diseases. 2015;9(5):e0003507.

33. Alexander ND, Cousens SN, Yahaya H, Abiose A, Jones BR. Ivermectin dose assessment without weighing scales. Bulletin of the World Health Organization. 1993;71(3-4):361-6.

34. Stunkard AJ, Sørensen T, Schulsinger F. Use of the Danish Adoption Register for the study of obesity and thinness. Research publications - Association for Research in Nervous and Mental Disease. 1983;60:115-20.

For peer review only

For peer review only - http://bmjopen.bmj.com/site/about/guidelines.xhtml

35. Australian Medicines Handbook [Internet]. Available from: https://amhonline.amh.net.au.acs.hcn.com.au/chapters/dermatological-drugs/scabicides-pediculicides/permethrin.

36. Marks M, Engelman D, Romani L, Mason D, Sokana O, Kama M, et al. Exploration of a simplified clinical examination for scabies to support public health decision-making. PLoS Neglected Tropical Diseases [electronic resource]. 2018;12(12):e0006996.

37. Engelman D, Fuller LC, Steer AC, for the International Alliance for the Control of Scabies Delphi p. Consensus criteria for the diagnosis of scabies: A Delphi study of international experts. PLOS Neglected Tropical Diseases. 2018;12(5):e0006549.

38. Engelman D, Yoshizumi J, Hay RJ, Osti M, Micali G, Norton S, et al. The 2020 IACS Consensus Criteria for the Diagnosis of Scabies. British Journal of Dermatology. 2020.

39. Osti MH, Sokana O, Gorae C, Whitfeld MJ, Steer AC, Engelman D. The diagnosis of scabies by non-expert examiners: A study of diagnostic accuracy. PLOS Neglected Tropical Diseases. 2019;13(8):e0007635.

40. Australian Public Assessment Report for Ivermectin. Woden, ACT, Australia: Commonwealth of Australia; 2013.

41. Guzzo CA, Furtek CI, Porras AG, Chen C, Tipping R, Clineschmidt CM, et al. Safety, Tolerability, and Pharmacokinetics of Escalating High Doses of Ivermectin in Healthy Adult Subjects. The Journal of Clinical Pharmacology. 2002;42(10):1122-33.

42. Ottesen EA, Hooper PJ, Bradley M, Biswas G. The Global Programme to Eliminate Lymphatic Filariasis: Health Impact after 8 Years. PLoS Neglected Tropical Diseases. 2008;2(10):e317.

43. Tielsch JM, Beeche A. Impact of ivermectin on illness and disability associated with onchocerciasis. Tropical Medicine & International Health. 2004;9(4):A45-A56.

44. Gardon J, Gardon-Wendel N, Demanga N, Kamgno J, Chippaux J-P, Boussinesq M. Serious reactions after mass treatment of onchocerciasis with ivermectin in an area endemic for Loa loa infection. The Lancet. 1997;350(9070):18-22.

45. Solomon Islands Essential Medicines List. Honiara, Solomon Islands: Ministry of Health and Medical Services; 2017.

46. Strong M, Johnstone P. Interventions for treating scabies. The Cochrane database of systematic reviews. 2007;2007(3):CD000320-CD.

47. Currie BJ, McCarthy JS. Permethrin and ivermectin for scabies. New England Journal of Medicine. 2010;362(8):717-25.

For peer review only

48. Hoffmann JC, Mossner R, Schon MP, Lippert U. Topical scabies therapy with permethrin is effective and well tolerated in infants younger than two months. J Dtsch Dermatol Ges. 2019;17(6):597-600.

49. Landau S, Stahl D. Sample size and power calculations for medical studies by simulation when closed form expressions are not available. Statistical Methods in Medical Research. 2013;22(3):324-45.

50. Provincial Profile of the 2009 Population & Housing Census: Western, Solomon Islands. 2009.

51. Hayes RJ, Moulton LH. Cluster randomised trials, second edition: CRC Press; 2017. 424 p.

52. Harris PA, Taylor R, Minor BL, Elliott V, Fernandez M, O'Neal L, et al. The REDCap consortium: Building an international community of software platform partners. Journal of Biomedical Informatics. 2019;95:103208.

53. Harris PA, Taylor R, Thielke R, Payne J, Gonzalez N, Conde JG. Research electronic data capture (REDCap)—A metadata-driven methodology and workflow process for providing translational research informatics support. Journal of Biomedical Informatics. 2009;42(2):377-81.

54. CONSORT. CONSORT Transparent Reporting of Trials [updated 2020.]. Available from: http://www.consort-statement.org/.

55. SPIRIT. SPIRIT Statement [updated 2020. Available from: https://www.spirit-statement.org/.

56. Chan A-W, Tetzlaff JM, Altman DG, Laupacis A, Gøtzsche PC, Krleža-Jerić K, et al. SPIRIT 2013 statement: defining standard protocol items for clinical trials. Ann Intern Med. 2013;158(3):200-7.

57. Mounsey KE, Bernigaud C, Chosidow O, McCarthy JS. Prospects for Moxidectin as a New Oral Treatment for Human Scabies. PLoS Neglected Tropical Diseases [electronic resource]. 2016;10(3):e0004389.

For peer review only - http://bmjopen.bmj.com/site/about/guidelines.xhtml

Figure legends/captions:

Figure 1. Study location in Solomon Islands

Figure 2. Study location in Western Province, Solomon Islands

Figure 3. Study flow diagram

For peer review only

Supplementary material

Skin examination training for nurses

For peer review only - http://bmjopen.bmj.com/site/about/guidelines.xhtml

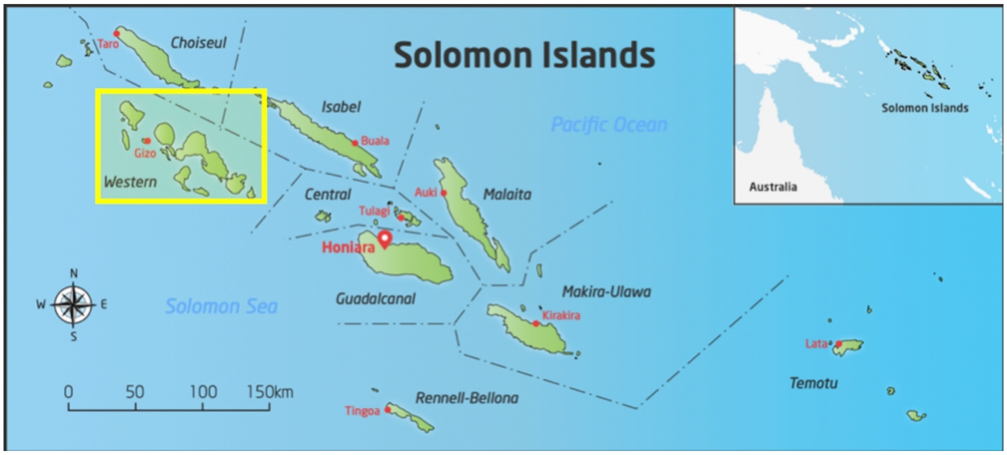

Figure 1. Study location in Solomon Islands

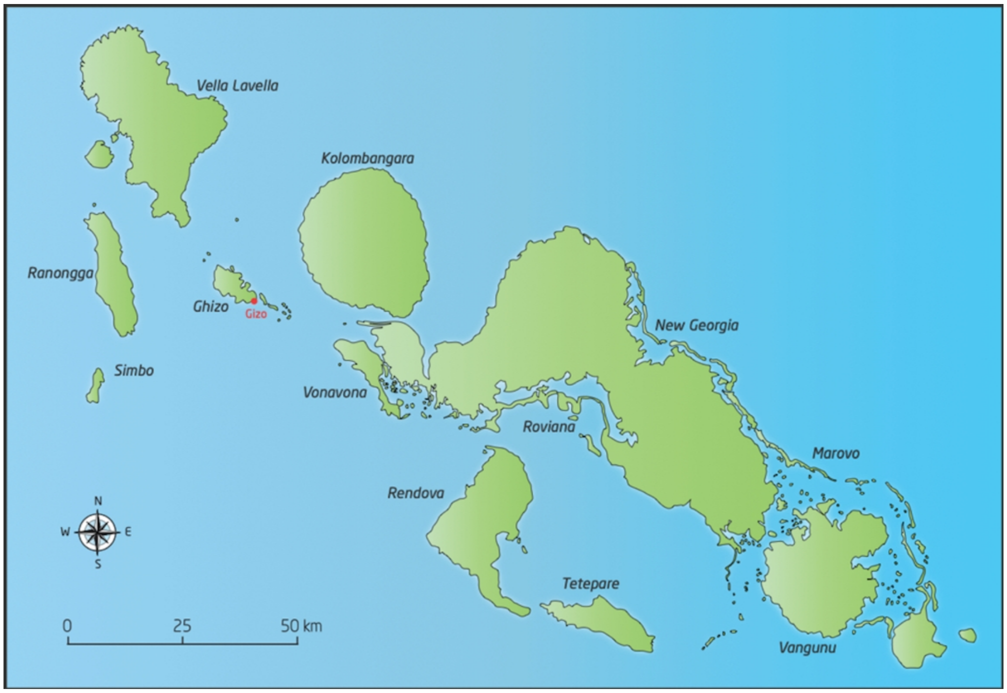

Figure 2. Study location in Western Province, Solomon Islands

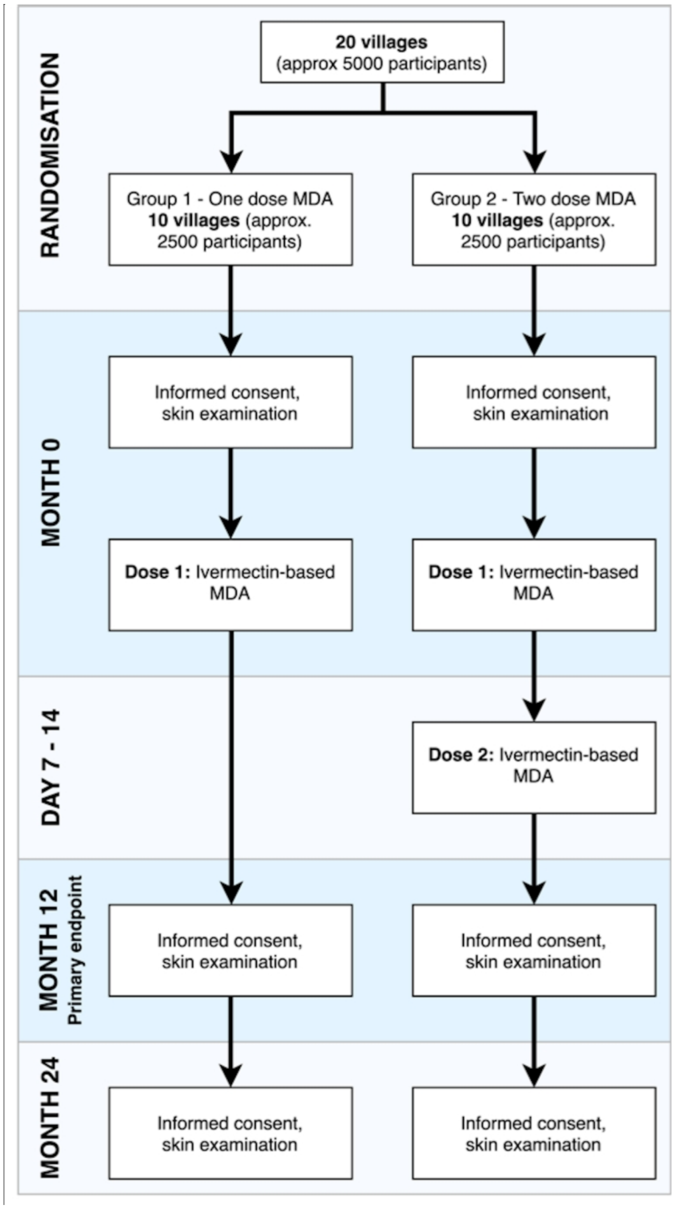

Figure 3. Study flow diagram

Participant Information Statement and Consent Form

Initial Visit

HREC Project Number: 38099A

Research Project Title: RISE: Regimens of Ivermectin for Scabies Elimination

Local Principal Investigator: Mr Oliver Sokana

Version Number: 4

Version Date: 25/02/19

Thank you for taking the time to read this **Participant Information Statement and Consent Form**. We would like to invite you to participate in a research project that is explained below.

This document is 5 pages long. Please make sure you have all the pages.

What is an Information Statement and Consent Form?

An Information and Consent Form tells you about the research project. It clearly explains exactly what the research project will involve. This information is to help you decide whether or not you would like to take part in the research. Please read it carefully.

Before you decide if you want to take part or not, you can ask us any questions you have about the project. You may want to talk about the project with your family, friends or health care worker.

Taking part in the research is up to you

It is your choice whether or not you take part in the research. You do not have to agree if you do not want to. If you decide you do not want to take part, it will not affect the treatment and care you get.

Signing the form

If you want to take part in the research, please sign the consent form at the end of this document. By signing the form you are telling us that you:

- Understand what you have read
- Have had a chance to ask questions and received satisfactory answers
- Consent to taking part in the project. We will give you a copy of this form to keep.

For peer review only - http://bmjopen.bmj.com/site/about/guidelines.xhtml

1. What is the research project about?

The main aim of this project is to get rid of the skin infection known as scabies. People often don't know that they have this infection. To get the best results we try to treat everyone to make sure we don't miss anyone with infection. Usually you need to take the same medicine twice to get rid of this infection, but you may only need to take it once. We are trying to figure out if we can get rid of scabies if everyone in your community takes this medicine once.

The medicine that is used to treat scabies can also treat some kinds of intestinal worms. We are also trying to figure out whether treatment of intestinal worms is better with two doses of the medicine, compared to one dose.

2. Who is funding this research project?

The project is organised by the Solomon Islands Ministry of Health, the Murdoch Children's Research Institute, the London School of Hygiene & Tropical Medicine, The Kirby Institute and the Australian National University.

3. Why am I being asked to take part?

Twenty villages in the Western Province of the Solomon Islands have been selected at random to have their skin checked, give a sample of their stool to look for intestinal worms, and be provided with medication for scabies. Everyone living in the village will be asked to participate.

4. What do I need to do in this research project?

If you agree to take part, we will record information about your age and gender and will take your height and weight. We will examine your skin for signs of scabies and other skin problems.

Photographs may be taken of any skin lesions or rashes. These photos will not include your face or head and will not be recognisable as belonging to you. The research team will check with you before taking any photographs. If you do not wish to have a photograph taken that is fine.

We will ask you some questions about how scabies affects you and your family, and some questions about your risk of intestinal worm infections.

If you are willing to provide a stool sample, we will send your stool sample (without your name on it) to a laboratory at Murdoch Children's Research Institute in Australia to test for intestinal worm infections.

We will ask you if you are willing to take the treatment for scabies. If you agree, we will provide this treatment. For most people in the study this will be a tablet called ivermectin. For some people who can't have ivermectin, including children less than 90cm in height and pregnant women, treatment will be with a cream called permethrin. This cream only treats scabies, not intestinal worm infections.

For peer review only - http://bmjopen.bmj.com/site/about/guidelines.xhtml
For peer review only

If you live in a community that has been allocated two doses of treatment for scabies, we will visit you again to provide this second dose one or two weeks after the first dose.

We will visit you again to examine your skin for scabies and collect stool samples 12 months after our first visit, and then again 12 months after that. This is so we can check how effective the treatment is.

5. Can I withdraw from the project?

You can stop taking part in the project at any time. You just need to tell us so. You do not need to tell us the reason why. If you leave the project we will use any information already collected unless you tell us not to.

6. What are the possible benefits for me and other people in the future?

You and your community will receive treatment for scabies. If our study shows that this is an effective strategy, we may be able to provide this treatment to help other people in the Solomon Islands and many other countries.

7. What are the possible risks, side-effects, discomforts and/or inconveniences?

Treatment for scabies is very effective and side effects are uncommon and quickly go away.

Ivermectin occasionally causes dizziness or tummy upset. Children less than 90cm in height and pregnant women should not take ivermectin and will be offered a cream instead.

Permethrin cream occasionally causes itch and stinging.

Having your skin examined for scabies is not uncomfortable or painful. The whole process, including asking questions and examination should take less than 10 minutes.

You have previously been informed about stool collection procedures, at the time of receiving the stool collection kit.

8. What will be done to make sure my information is confidential?

Any information we collect for this project that can identify you will be treated confidentially, except as required by law. Nothing that could reveal your identity will be disclosed outside the project.

9. Will I be informed of the results when the research project is finished?

Results of the project will help us understand the best way to treat scabies in communities in the Solomon Islands and elsewhere. Results will be published in the medical literature, and a report summarizing the results will be sent to your community health worker who will pass on the

For peer review only - http://bmjopen.bmj.com/site/about/guidelines.xhtml

information to you. You and your family will not be personally identified in any report or publication.

10. Who should I contact for more information?

If you would like more information about the project, please contact:

Name: Mr Oliver Sokana

Contact telephone: 769 1615

Email: sokanao@moh.gov.sb

OR

Name: Prof. Andrew Steer

Contact telephone: +61 (3) 9345 5522

Email: Andrew.Steer@rch.org.au

If you:
- Have any concerns or complaints about the project
- Are worried about your rights as a research participant
- Would like to speak to someone independent of the project.

You can contact the Solomon Islands National Health Research Ethics Committee by telephone on (+677) 37295, or you can contact the Director of Research Ethics & Governance at The Royal Children's Hospital Melbourne by telephone on +61 (3) 9345 5044.

For peer review only - http://bmjopen.bmj.com/site/about/guidelines.xhtml
For peer review only

CONSENT FORM

HREC Project Number: 38099A

Research Project Title: RISE: Regimens of Ivermectin for Scabies Elimination

Local Principal Investigator: Mr Oliver Sokana

Version Number: 4

Version Date: 25/02/2019

- I have read this information statement and I understand its contents.
- I understand I have to do to be involved in this project.
- I understand the risks I could face because of my involvement in this project.
- I voluntarily consent to take part in this research project.
- I have had an opportunity to ask questions about the project and I am satisfied with the answers I have received.
- I understand that this project has been approved by The Solomon Islands National Health Research Ethics Committee and the Royal Children's Hospital Melbourne Human Research Ethics Committee. I understand that the project and any updates will be carried out in line with the National Statement on Ethical Conduct in Human Research (2007).
- I understand I will receive a copy of this Information Statement and Consent Form.

I consent/give consent for ______________________________ to take part in this study

Participant Name

☐ I consent/give consent for stool sample to be analysed for intestinal worm infections *(tick box if applicable)*

Participant Signature or Fingerprint	Date	
Name of Witness to Participant's Signature	Witness Signature	Date

Declaration by researcher: I have explained the project to the participant who has signed above. I believe that they understand the purpose, extent and possible risks of their involvement in this project.

Research Team Member Name	Research Team Member Signature	Date

Note: All parties signing the Consent Form must date their own signature.

For peer review only - http://bmjopen.bmj.com/site/about/guidelines.xhtml

SUPPLEMENTARY MATERIAL

Skin examination training for nurses

Training was delivered by two Australians doctors with experience in scabies and other tropical skin conditions. Training materials were developed based on material previously delivered in the Solomon Islands and Fiji (Table S1). The examination and history component of the training was focused on identifying the relevant features required for the diagnosis of scabies and impetigo. Other differential skin diagnoses that were relevant to the setting were also included.

Training consisted of two stages; classroom training and practical training at a primary school. The classroom training content included background information on the importance of scabies as a public health problem in the Solomon Islands, further context for the study, details on global and local prevalence of scabies, complications of the diseases and basic treatment concepts. Training on the diagnosis of scabies was based on the 2020 International Alliance for the Control of Scabies Consensus Criteria for the Diagnosis of Scabies.(1)

Clinical examination was limited to exposed areas of skin – particularly the arms from the mid-upper arm to the finger tips, and the legs from the mid-upper though to the toes. A brief history component containing questions about itch and contact history was incorporated. Terminology and definitions used in training were consistent with the World Health Organization 2018 training guide, "Recognizing neglected tropical diseases through changes on the skin".(2)

For peer review only

For peer review only - http://bmjopen.bmj.com/site/about/guidelines.xhtml

<u>Table S1 – Overview of training</u>

**Part 1: Classroom training**
1.1 Scabies
What is scabies?
How do people get scabies?
How common is scabies?
What problems do scabies cause?
How can scabies be treated?
How can we get rid of scabies in the community?
1.2 Approach to diagnosis
About the skin
Dermatological terms
IACS criteria
History taking
Examination
Differential diagnoses
1.3 Facilitated practice with clinical images
**Part 2: Supervised field training**
2.1 Practice examination
**Part 3: Assessment**
3.1 Slide assessment
3.2 Field assessment

For peer review only

REFERENCES

1. Engelman D, Yoshizumi J, Hay RJ, Osti M, Micali G, Norton S, et al. The 2020 IACS Consensus Criteria for the Diagnosis of Scabies. British Journal of Dermatology. 2020.

2. World Health O. Recognizing neglected tropical diseases through changes on the skin: a training guide for front-line health workers. Geneva: World Health Organization; 2018 2018.

For peer review only

Reporting checklist for protocol of a clinical trial.

Based on the SPIRIT guidelines.

Instructions to authors

Complete this checklist by entering the page numbers from your manuscript where readers will find each of the items listed below.

Your article may not currently address all the items on the checklist. Please modify your text to include the missing information. If you are certain that an item does not apply, please write "n/a" and provide a short explanation.

Upload your completed checklist as an extra file when you submit to a journal.

In your methods section, say that you used the SPIRITreporting guidelines, and cite them as:

Chan A-W, Tetzlaff JM, Altman DG, Laupacis A, Gøtzsche PC, Krleža-Jerić K, Hróbjartsson A, Mann H, Dickersin K, Berlin J, Doré C, Parulekar W, Summerskill W, Groves T, Schulz K, Sox H, Rockhold FW, Rennie D, Moher D. SPIRIT 2013 Statement: Defining standard protocol items for clinical trials. Ann Intern Med. 2013;158(3):200-207

		Reporting Item	Page Number
**Administrative information**			
Title	#1	Descriptive title identifying the study design, population, interventions, and, if applicable, trial acronym	1
Trial registration	#2a	Trial identifier and registry name. If not yet registered, name of intended registry	4
Trial registration: data set	#2b	All items from the World Health Organization Trial Registration Data Set	1, 4, 24
Protocol version	#3	Date and version identifier	N/A V4 25/2/19
Funding	#4	Sources and types of financial, material, and other support	24

For peer review only

Roles and responsibilities: contributorship	#5a	Names, affiliations, and roles of protocol contributors	1
Roles and responsibilities: sponsor contact information	#5b	Name and contact information for the trial sponsor	1, 2
Roles and responsibilities: sponsor and funder	#5c	Role of study sponsor and funders, if any, in study design; collection, management, analysis, and interpretation of data; writing of the report; and the decision to submit the report for publication, including whether they will have ultimate authority over any of these activities	24
Roles and responsibilities: committees	#5d	Composition, roles, and responsibilities of the coordinating centre, steering committee, endpoint adjudication committee, data management team, and other individuals or groups overseeing the trial, if applicable (see Item 21a for data monitoring committee)	1, 24-25
**Introduction**			
Background and rationale	#6a	Description of research question and justification for undertaking the trial, including summary of relevant studies (published and unpublished) examining benefits and harms for each intervention	6-8
Background and rationale: choice of comparators	#6b	Explanation for choice of comparators	9
Objectives	#7	Specific objectives or hypotheses	10
Trial design	#8	Description of trial design including type of trial (eg, parallel group, crossover, factorial, single group), allocation ratio, and framework (eg, superiority, equivalence, non-inferiority, exploratory)	9
**Methods: Participants,**			

For peer review only

**interventions, and outcomes**			
Study setting	#9	Description of study settings (eg, community clinic, academic hospital) and list of countries where data will be collected. Reference to where list of study sites can be obtained	11
Eligibility criteria	#10	Inclusion and exclusion criteria for participants. If applicable, eligibility criteria for study centres and individuals who will perform the interventions (eg, surgeons, psychotherapists)	11-12
Interventions: description	#11a	Interventions for each group with sufficient detail to allow replication, including how and when they will be administered	13-14
Interventions: modifications	#11b	Criteria for discontinuing or modifying allocated interventions for a given trial participant (eg, drug dose change in response to harms, participant request, or improving / worsening disease)	16
Interventions: adherance	#11c	Strategies to improve adherence to intervention protocols, and any procedures for monitoring adherence (eg, drug tablet return; laboratory tests)	13-14
Interventions: concomitant care	#11d	Relevant concomitant care and interventions that are permitted or prohibited during the trial	12
Outcomes	#12	Primary, secondary, and other outcomes, including the specific measurement variable (eg, systolic blood pressure), analysis metric (eg, change from baseline, final value, time to event), method of aggregation (eg, median, proportion), and time point for each outcome. Explanation of the clinical relevance of chosen efficacy and harm outcomes is strongly recommended	14-16
Participant timeline	#13	Time schedule of enrolment, interventions (including any run-ins and washouts), assessments, and visits for participants. A schematic diagram is highly recommended (see Figure)	13-15, Fig. 3
Sample size	#14	Estimated number of participants needed to achieve study objectives and how it was determined, including	17-18

For peer review only

		clinical and statistical assumptions supporting any sample size calculations	
Recruitment	#15	Strategies for achieving adequate participant enrolment to reach target sample size	12-13
**Methods: Assignment of interventions (for controlled trials)**			
Allocation: sequence generation	#16a	Method of generating the allocation sequence (eg, computer-generated random numbers), and list of any factors for stratification. To reduce predictability of a random sequence, details of any planned restriction (eg, blocking) should be provided in a separate document that is unavailable to those who enrol participants or assign interventions	18
Allocation concealment mechanism	#16b	Mechanism of implementing the allocation sequence (eg, central telephone; sequentially numbered, opaque, sealed envelopes), describing any steps to conceal the sequence until interventions are assigned	N/A unblinded study
Allocation: implementation	#16c	Who will generate the allocation sequence, who will enrol participants, and who will assign participants to interventions	18
Blinding (masking)	#17a	Who will be blinded after assignment to interventions (eg, trial participants, care providers, outcome assessors, data analysts), and how	9
Blinding (masking): emergency unblinding	#17b	If blinded, circumstances under which unblinding is permissible, and procedure for revealing a participant's allocated intervention during the trial	N/A unblinded study
**Methods: Data collection, management, and analysis**			
Data collection plan	#18a	Plans for assessment and collection of outcome, baseline, and other trial data, including any related processes to promote data quality (eg, duplicate	14-16

For peer review only

		measurements, training of assessors) and a description of study instruments (eg, questionnaires, laboratory tests) along with their reliability and validity, if known. Reference to where data collection forms can be found, if not in the protocol	
Data collection plan: retention	#18b	Plans to promote participant retention and complete follow-up, including list of any outcome data to be collected for participants who discontinue or deviate from intervention protocols	12-13
Data management	#19	Plans for data entry, coding, security, and storage, including any related processes to promote data quality (eg, double data entry; range checks for data values). Reference to where details of data management procedures can be found, if not in the protocol	20
Statistics: outcomes	#20a	Statistical methods for analysing primary and secondary outcomes. Reference to where other details of the statistical analysis plan can be found, if not in the protocol	18-19
Statistics: additional analyses	#20b	Methods for any additional analyses (eg, subgroup and adjusted analyses)	18-19
Statistics: analysis population and missing data	#20c	Definition of analysis population relating to protocol non-adherence (eg, as randomised analysis), and any statistical methods to handle missing data (eg, multiple imputation)	18-19
**Methods: Monitoring**			
Data monitoring: formal committee	#21a	Composition of data monitoring committee (DMC); summary of its role and reporting structure; statement of whether it is independent from the sponsor and competing interests; and reference to where further details about its charter can be found, if not in the protocol. Alternatively, an explanation of why a DMC is not needed	16-17
Data monitoring: interim analysis	#21b	Description of any interim analyses and stopping guidelines, including who will have access to these	N/A Single intervention. DSMB

For peer review only

		interim results and make the final decision to terminate the trial	and Principal Investigator can stop the study.
Harms	#22	Plans for collecting, assessing, reporting, and managing solicited and spontaneously reported adverse events and other unintended effects of trial interventions or trial conduct	16-17
Auditing	#23	Frequency and procedures for auditing trial conduct, if any, and whether the process will be independent from investigators and the sponsor	17
**Ethics and dissemination**			
Research ethics approval	#24	Plans for seeking research ethics committee / institutional review board (REC / IRB) approval	20-21
Protocol amendments	#25	Plans for communicating important protocol modifications (eg, changes to eligibility criteria, outcomes, analyses) to relevant parties (eg, investigators, REC / IRBs, trial participants, trial registries, journals, regulators)	N/A
Consent or assent	#26a	Who will obtain informed consent or assent from potential trial participants or authorised surrogates, and how (see Item 32)	11-13
Consent or assent: ancillary studies	#26b	Additional consent provisions for collection and use of participant data and biological specimens in ancillary studies, if applicable	N/A No biological specimens
Confidentiality	#27	How personal information about potential and enrolled participants will be collected, shared, and maintained in order to protect confidentiality before, during, and after the trial	20
Declaration of interests	#28	Financial and other competing interests for principal investigators for the overall trial and each study site	24
Data access	#29	Statement of who will have access to the final trial dataset, and disclosure of contractual agreements that limit such access for investigators	21

For peer review only

Ancillary and post trial care	#30	Provisions, if any, for ancillary and post-trial care, and for compensation to those who suffer harm from trial participation	N/A If required post-trial care to be delivered through local health system
Dissemination policy: trial results	#31a	Plans for investigators and sponsor to communicate trial results to participants, healthcare professionals, the public, and other relevant groups (eg, via publication, reporting in results databases, or other data sharing arrangements), including any publication restrictions	18
Dissemination policy: authorship	#31b	Authorship eligibility guidelines and any intended use of professional writers	25
Dissemination policy: reproducible research	#31c	Plans, if any, for granting public access to the full protocol, participant-level dataset, and statistical code	21
**Appendices**			
Informed consent materials	#32	Model consent form and other related documentation given to participants and authorised surrogates	N/A
Biological specimens	#33	Plans for collection, laboratory evaluation, and storage of biological specimens for genetic or molecular analysis in the current trial and for future use in ancillary studies, if applicable	N/A No biological specimens to be collected

Notes:

- 3: N/A V4 25/2/19
- 13: 13-15, Fig. 3
- 16b: N/A unblinded study
- 17b: N/A unblinded study
- 21b: N/A Single intervention. DSMB and Principal Investigator can stop the study.
- 26b: N/A No biological specimens
- 30: N/A If required post-trial care to be delivered through local health system
- 33: N/A No biological specimens to be collected The SPIRIT checklist is distributed under the terms of the Creative Commons Attribution License CC-BY-ND 3.0. This checklist was completed on 23. January

For peer review only

2020 using https://www.goodreports.org/, a tool made by the EQUATOR Network in collaboration with Penelope.ai

For peer review only

BMJ Open

Protocol for a cluster randomised non-inferiority trial of one versus two doses of ivermectin for the control of scabies using a mass drug administration strategy (the RISE study)

Journal:	*BMJ Open*
Manuscript ID	bmjopen-2020-037305.R2
Article Type:	Protocol
Date Submitted by the Author:	20-Jul-2020
Complete List of Authors:	Lake, Susanna; Murdoch Childrens Research Institute, Tropical Disease Research Group; The University of Melbourne, Department of Paediatrics<br>Phelan, Sophie; University of New South Wales, The Kirby Institute<br>Engelman, Daniel; Murdoch Childrens Research Institute, Tropical Disease Research Group; The University of Melbourne, Department of Paediatrics<br>Sokana, Oliver; Ministry of Health and Medical Services<br>Nasi, Titus; Ministry of Health and Medical Services<br>Boara, Dickson; Ministry of Health and Medical Services<br>Gorae, Christina; Ministry of Health and Medical Services<br>Schuster, Tibor; McGill University, Clinical Epidemiology and Biostatistics Unit<br>Grobler, Anneke; Murdoch Childrens Research Institute, Clinical Epidemiology and Biostatistics Unit<br>Osti, Millicent; Murdoch Childrens Research Institute, Tropical Disease Research Group<br>Andrews, Ross; Australian National University<br>Marks, Michael; London School of Hygiene and Tropical Medicine, Clinical Research Department; Hospital for Tropical Diseases,<br>Whitfeld, Margot; St Vincent's Hospital , Department of Dermatology<br>Romani, Lucia; University of New South Wales, The Kirby Institute<br>Kaldor, John; University of New South Wales, The Kirby Institute<br>Steer, Andrew; Murdoch Childrens Research Institute, Tropical Disease Research Group; The University of Melbourne, Department of Paediatrics
**Primary Subject Heading**:	Global health
Secondary Subject Heading:	Dermatology
Keywords:	TROPICAL MEDICINE, INFECTIOUS DISEASES, DERMATOLOGY, International health services < HEALTH SERVICES ADMINISTRATION & MANAGEMENT

SCHOLARONE™ Manuscripts

I, the Submitting Author has the right to grant and does grant on behalf of all authors of the Work (as defined in the below author licence), an exclusive licence and/or a non-exclusive licence for contributions from authors who are: i) UK Crown employees; ii) where BMJ has agreed a CC-BY licence shall apply, and/or iii) in accordance with the terms applicable for US Federal Government officers or employees acting as part of their official duties; on a worldwide, perpetual, irrevocable, royalty-free basis to BMJ Publishing Group Ltd ("BMJ") its licensees and where the relevant Journal is co-owned by BMJ to the co-owners of the Journal, to publish the Work in this journal and any other BMJ products and to exploit all rights, as set out in our licence.

The Submitting Author accepts and understands that any supply made under these terms is made by BMJ to the Submitting Author unless you are acting as an employee on behalf of your employer or a postgraduate student of an affiliated institution which is paying any applicable article publishing charge ("APC") for Open Access articles. Where the Submitting Author wishes to make the Work available on an Open Access basis (and intends to pay the relevant APC), the terms of reuse of such Open Access shall be governed by a Creative Commons licence – details of these licences and which Creative Commons licence will apply to this Work are set out in our licence referred to above.

Other than as permitted in any relevant BMJ Author's Self Archiving Policies, I confirm this Work has not been accepted for publication elsewhere, is not being considered for publication elsewhere and does not duplicate material already published. I confirm all authors consent to publication of this Work and authorise the granting of this licence.

For peer review only

Protocol for a cluster randomised non-inferiority trial of one versus two doses of ivermectin for the control of scabies using a mass drug administration strategy (the RISE study)

Susanna J. Lake MIPH (1,2,3)

Sophie L. Phelan MIPH MAE (4)

Daniel Engelman PhD (1,2,3)

Oliver Sokana MPH(5)

Titus Nasi MCH(5)

Dickson Boara PgDipHSM (5)

Christina Gorae (5)

Tibor Schuster PhD (6)

Anneke C. Grobler PhD (7)

Millicent H. Osti MBBS (1,2)

Ross Andrews PhD (8)

Michael Marks PhD (9,10)

Margot J. Whitfeld FACD (11)

Lucia Romani PhD (4)

John M. Kaldor PhD (4)

Andrew C. Steer PhD (1,2,3)

1. Tropical Diseases Research Group, Murdoch Children's Research Institute, Melbourne, Australia
2. Department of Paediatrics, University of Melbourne, Melbourne, Australia
3. Melbourne Children's Global Health, Melbourne, Australia
4. Kirby Institute, University of New South Wales, Sydney, Australia

For peer review only

5. Ministry of Health and Medical Services, Solomon Islands
6. McGill University, Montreal Canada
7. Clinical Epidemiology and Biostatistics Unit, Murdoch Children's Research Institute, Melbourne, Australia
8. Australian National University, Canberra, Australia
9. London School of Hygiene and Tropical Medicine, London, United Kingdom
10. Hospital for Tropical Diseases, London, United Kingdom
11. Department of Dermatology, St Vincent's Hospital, Sydney, Australia

Correspondence to:

Professor Andrew C Steer; Andrew.Steer@rch.org.au

Keywords:

Scabies

Mass drug administration

Ivermectin

Solomon Islands

For peer review only

ABSTRACT

Introduction

Scabies is a significant contributor to global morbidity, affecting approximately 200 million people at any time. Scabies is endemic in many resource-limited tropical settings. Bacterial skin infection (impetigo) frequently complicates scabies infestation in these settings. Community-wide ivermectin-based mass drug administration (MDA) is an effective control strategy for scabies in island settings, with a single round of MDA reducing population prevalence by around 90%. However, current two-dose regimens present a number of barriers to programmatic MDA implementation. We designed the Regimens of Ivermectin for Scabies Elimination (RISE) trial to investigate whether one-dose MDA may be as effective as two-dose MDA in controlling scabies in high-prevalence settings.

Methods and analysis

RISE is a cluster randomised non-inferiority trial. The study will be conducted in 20 isolated villages in Western Province of Solomon Islands where population prevalence of scabies is approximately 20%. Villages will be randomly allocated to receive either one dose or two doses of ivermectin-based MDA in a 1:1 ratio. The primary objective of the study is to determine if ivermectin-based MDA with one dose is as effective as MDA with two doses in reducing the prevalence of scabies after 12 months. Secondary objectives include the effect of ivermectin-based MDA on impetigo prevalence after 12 months and 24 months, the prevalence of scabies at 24 months after the intervention, the impact on presentations to health facilities with scabies and impetigo, and the safety of one-dose and two-dose MDA.

For peer review only

Ethics and dissemination

This trial has been approved by the ethics review committees of the Solomon Islands and the Royal Children's Hospital, Australia. Results will be disseminated in peer-reviewed publications and in meetings with the Solomon Islands Ministry of Health and Medical Services and participating communities.

Trial Registration

Australian New Zealand Clinical Trials Registry: ACTRN12618001086257. Date registered: 28 June, 2018.

For peer review only

ARTICLE SUMMARY

Strengths and limitations of this study

- The cluster randomised study design follows the implementation of the intervention at a village level
- Follow up at both 12 and 24 months will demonstrate longer term effects of the intervention
- The sample size (5000 people across 20 villages) achieves a statistical power of 80%
- This study is being conducted in partnership with the Solomon Islands Ministry of Health and Medical Service and will build the capacity of local nursing staff as well as being conducted in a culturally sensitive manner
- Scabies prevalence is high in the isolated island villages where this study will be conducted (approximately 20%), therefore results may not be transferable to lower prevalence or urban settings.

For peer review only

BACKGROUND

Scabies is a neglected tropical disease (NTD) caused by infestation with the mite *Sarcoptes scabiei* var. *hominis*. Scabies is a significant contributor to global morbidity, estimated to cause 455 million annual incident cases.(1, 2) Transmission occurs primarily as a result of skin-to-skin contact (and rarely due to fomites) and is more common in overcrowded settings, including in many tropical environments where crowding and poverty are prevalent and access to treatment limited.(3, 4) The burden of disease is substantial in many Pacific Island Countries where scabies affects 1 in 5 people and up to 1 in 2 children.(5)

Scabies infestation causes intense itch and discomfort. Furthermore, it is responsible for a considerable proportion of bacterial skin infection (impetigo) in many resource-limited settings.(6-8) Scabies causes a breach in the skin barrier from scabetic lesions and subsequent scratching, creating an entry point for bacteria including *Staphylococcus aureus* and *Streptococcus pyogenes.* Resulting impetigo can in turn cause severe infection and immune-mediated disease including sepsis, glomerulonephritis and possibly rheumatic fever.(9-12)

Treatment guidelines for scabies recommend treatment of the infected individual as well as household contacts.(13, 14) Most guidelines recommend treatment with topical acaracides such as permethrin or benzoyl benzoate.(13, 14) These medications are effective, if applied to all affected areas for the correct duration, but re-infestation frequently occurs in highly endemic settings where individuals may be exposed to infected household or community members, many of whom may be asymptomatic.(15) Therefore, attention has shifted to simultaneous treatment of whole communities, including those without symptoms of infestation, to reduce prevalence and the rate of transmission. (16) This strategy of mass drug

For peer review only

For peer review only - http://bmjopen.bmj.com/site/about/guidelines.xhtml

administration (MDA) has been used to successfully control a number of NTDs including onchocerciasis, lymphatic filariasis (LF), trachoma and soil-transmitted helminths and there is a growing body of evidence to support MDA for scabies control.(17-22)

Ivermectin is an antiparasitic drug in the avermectin class that is active against the scabies mite. Ivermectin-based MDA for scabies involves offering ivermectin treatment to the whole community, with the exception of young children, pregnant women and others with a contraindication to ivermectin. Permethrin cream is offered as an alternative to ivermectin for these groups. Several studies in Pacific Island Countries with high-prevalence have shown ivermectin-based MDA can reduce the population prevalence of scabies by around 90%.(7, 17, 23) The SHIFT study in Fiji was the first comparative study to demonstrate the effectiveness of ivermectin MDA for scabies control, finding a reduction in the population prevalence of scabies from 32% at baseline to less than 2% at 12 months.(21) These trials have all used an MDA strategy involving two doses of medication, given 7-14 days apart (either to the whole community, or those with clinical signs of scabies).(7, 23) This is consistent with clinical recommendations for treatment of individuals.(13) Ivermectin is known to lack ovicidal activity, therefore the second dose aims to kill newly hatched mites.(24)

In 2017, the World Health Organization (WHO) recognised scabies as a NTD, and identified the need for public health action to control scabies in endemic settings.(9) The Strategic and Technical Advisory Group on Neglected Tropical Diseases called for further research into control strategies for scabies and the development of guidelines for the public health use of avermectins.(25)

For peer review only - http://bmjopen.bmj.com/site/about/guidelines.xhtml

While ivermectin-based MDA shows great promise as a control strategy for scabies, the requirement for two doses of medication at each MDA round presents barriers to implementation. Drug and implementation costs are doubled compared to single-dose MDA. Distribution is more complex and integration with programs for other NTDs is difficult. These challenges are amplified in remote island settings where the population is dispersed across difficult to reach villages and funding for programs is limited. These hurdles may be prohibitive to wide-spread implementation of scabies control, particularly in low-income settings. Therefore, the optimum dosing strategy for MDA remains an important knowledge gap.(9) For this reason we designed the Regimens of Ivermectin for Scabies Elimination (RISE) trial.

For peer review only

METHODS AND ANALYSIS

Study Design

The RISE trial is a prospective, open-label comparison of one dose versus two doses of ivermectin-based MDA for the population-level control of scabies (Table 1). Using a cluster-randomised design, 20 villages will be randomised to one of two intervention groups in a 1:1 ratio. Randomisation will occur at the village level, rather than the individual level, as the objective is to determine the dosing regimen for controlling scabies within whole communities. Randomisation minimises the possibility of the anticipated difference in the outcome between each group being confounded. A two-dose regimen is an appropriate comparator (rather than no-treatment or placebo) as two-doses of ivermectin-based MDA is the currently accepted dosing regimen. We chose a non-inferiority design because it is unlikely that a one-dose regimen would be superior in effectiveness to a two-dose regimen. However, the logistic and pragmatic advantages of a one-dose regimen compared to a two-dose regimen make a non-inferior study appealing.

The prevalence of scabies and impetigo will be measured before the intervention (baseline), and repeated at 12 months and 24 months after the intervention. To measure a secondary outcome the standard Solomon Islands Ministry of Health and Medical Services (MHMS) health facility reporting processes will be used to capture the number of presentations to health facilities with scabies and impetigo in the study catchment area for the 12 month period before the intervention and for the 24 month period after the intervention.

For peer review only - http://bmjopen.bmj.com/site/about/guidelines.xhtml

Table 1. Key features of the RISE trial

**Primary objective**	To determine if ivermectin-based mass drug administration (MDA) with one dose is non-inferior to two-doses in reducing prevalence of scabies at 12 months
**Secondary objectives**	Impact of one versus two dose ivermectin-based MDA on:<br>- Population prevalence of scabies at 24 months<br>- Population prevalence of impetigo at 12 and 24 months<br>- Number of presentations to health clinics with scabies and impetigo<br>- Number of adverse events measured by passive surveillance in the 12 month period following MDA
**Design**	Prospective, open-label comparison using a cluster randomised design
**Sample size**	20 villages (approximately 5000 participants), randomised in a 1:1 ratio to each intervention group
**Intervention**	Group 1: one dose of ivermectin-based MDA<br>Group 2: two doses of ivermectin-based MDA given 7-14 days apart
**Study setting**	Western Province, Solomon Islands (scabies prevalence approximately 20%)
**Inclusion criteria**	All residents in the study villages
**Exclusion criteria**	Participants who meet exclusion criteria will not receive treatment but will still be eligible to enrol in study and undergo skin examination<br>Exclusion criteria are: allergy to ivermectin or permethrin; treatment within the last 7 days with ivermectin or permethrin; participant declines treatment<br>If ivermectin is contraindicated, topical permethrin will be offered. Contraindications for ivermectin include: pregnancy; breastfeeding an infant less than seven days old; age less than two years; height less than 90 cm; concurrent medication that may interact with ivermectin (for example, warfarin); or severe acute or chronic illness on the day of MDA
**Outcome measures**	Presence of scabies and impetigo measured by clinical examination<br>Conducted by trained nurses and assessed using the 2020 International Alliance for the Control of Scabies criteria<br>Assessments will be conducted at baseline, 12 months and 24 months

For peer review only

Aims

The primary objective of this study is to determine if ivermectin-based MDA with one dose is non-inferior to two doses in reducing the prevalence of scabies 12 months after the intervention. The secondary objectives are to assess the impact of ivermectin-based MDA on: population prevalence of scabies after 24 months; population prevalence of impetigo after 12 months and 24 months; the number of presentations to health clinics with scabies and impetigo before and after the intervention; the number of adverse events measured by passive surveillance in the 12-months after MDA in each study group. The trial will also be measuring outcomes related to the impact of ivermectin MDA on the prevalence and intensity of soil transmitted helminths however this paper focuses on scabies and impetigo outcomes.

Rationale

This study uses ivermectin-based MDA because the SHIFT trial in Fiji demonstrated the greatest reduction in scabies prevalence after ivermectin-based MDA.(21) Ivermectin is currently the only oral therapy available for scabies and it allows greater compliance than with topical therapy. Oral therapy can be directly observed, ensuring adherence to treatment. Although ivermectin only kills the mature scabies mite and not the eggs, a single dose of treatment simultaneously administered to a whole village may reduce transmission sufficiently to reduce population prevalence. A recent Cochrane review did not find a difference in efficacy of one dose of oral ivermectin compared to two doses of oral ivermectin, but confidence in the effect estimates was low to moderate, with poor reporting a major limitation.(26) A retrospective study in Zanzibar of six rounds of annual single dose ivermectin MDA for LF showed a 68-98% decline in clinical presentations and treatments for

For peer review only

scabies, suggesting a one-dose strategy may significantly reduce transmission.(27) By contrast, annual ivermectin MDA for LF did not reduce the prevalence of scabies in Tanzania where the baseline prevalence was less than 5%.(28)

Study setting and participants

The study will be conducted in Western Province of Solomon Islands (Figures 1 and 2). Solomon Islands is a nation in the South Pacific with a population of over 650,000 spread across 900 islands, a geography that presents many challenges for health service delivery.(29) Solomon Islands is classified as a least developed country. It is ranked 152 out of 189 on the Human Development Index.(30) The majority of the population depend on subsistence agriculture in rural locations. We chose Western Province for this study for several reasons: first, there is a high burden of scabies - a 2014 survey estimated an all-age scabies prevalence of 19.2%;(6) second, we expect that there will be relatively little mixing between villages because of the island geography of isolated villages with no road transport; third, there are many villages of appropriate population size (between 180 and 300) for the cluster-randomised design.

Twenty villages will be selected after close consultation with the MHMS. Criteria for selecting the villages include a population of between 180 and 300 people, geographic isolation and willingness to participate in the study. All residents of the 20 selected villages will be eligible to participate. If a resident of a two-dose village does not take the first dose of medication they will still be eligible to take a dose when the team returns to the village for the second dose.

For peer review only

This is a community-based study, with analysis conducted at the village level, and therefore all residents are eligible to participate in the follow-up assessment at 12 months and 24 months, regardless of whether they received treatment at baseline. Written informed consent will be obtained from all participants. Participants under the age 18 years will require written consent to be provided by a parent or guardian. Consent will be obtained at each study timepoint (baseline, 12 months and 24 months) (see supplementary file 1).

Inclusion and exclusion criteria

Inclusion criteria. All participants who provide consent are eligible to participate. If exclusion criteria for treatment are met, then consented participants are still eligible to have their skin examined.

Exclusion criteria. Participants who meet any of the following criteria will not receive treatment, but will be eligible to enrol in the study and undergo skin examination: allergy to ivermectin or permethrin; treatment within the last 7 days with ivermectin or permethrin; declines treatment.

If ivermectin is contraindicated, then topical permethrin will be offered. The contraindications for ivermectin are: pregnancy; breastfeeding an infant less than seven days old; age less than two years; height less than 90 cm; concurrent medication that may interact with ivermectin (for example, warfarin); or severe acute or chronic illness on the day of MDA.

For peer review only

Patient and public involvement statement

The trial was designed in close consultation with stakeholders at the Solomon Islands MHMS to ensure it was culturally appropriate for the local setting. Staff from the Solomon Islands MHMS contributed to study design and identification of study sites. The study team will comprise a majority of Solomon Islander staff from Western Province. Staff are able to communicate in the local regional languages.

A team of health promotion officers from the Solomon Islands MHMS will conduct community awareness in each village, approximately one month prior to MDA. An illustrated information leaflet outlining the study design as well as information about scabies and the treatments will be provided during community visits. A participant information statement that contains contact details for the principal investigator and local investigator will be made available to all village residents. Community awareness and the informed consent process will be conducted in Solomon Islands Pijin and study staff who speak the local regional language will be available to provide further information or clarification as required. Results of the study will be communicated to community leaders and members by the study team.

Intervention

Oral ivermectin will be offered to all participants, unless there is a contraindication to ivermectin. A dose of 200 μg/kg of ivermectin is recommended for the treatment of individuals with scabies.(13, 31) We will aim to dose ivermectin within a range of 150-250 μg/kg, as this dose has been effective in previous trials.(21) We will use 6 mg scored tablets.

For peer review only - http://bmjopen.bmj.com/site/about/guidelines.xhtml

Doses will be rounded to the nearest 3 mg. The tablets will be accurately halved on the score line using a pill cutter as required. As weight scales are generally unsuitable for implementation of MDA, we will use dosing strategies appropriate for larger scale programmatic roll-out.

We will use height-based dosing for children aged less than 15 years, with doses ranging from 3 mg to 12 mg, as is standard for MDA for onchocerciasis and lymphatic filariasis.(32, 33) Adults will receive a standard dose of 12 mg, with doses adjusted based on visual assessment of body shape (9 mg for adults assessed to be a malnourished, 15 mg for adults assessed to be obese). Drug distribution staff will make these assessments based on a series of body shape silhouettes.(34) Dosing of medications for MDA based on physical appearance has been shown to be accurate and safe.(33) Staff will undergo training and validation for these dosing techniques. Ivermectin will be administered by trained study staff who will directly observe swallowing of the tablets.

Topical permethrin 5% cream will be given to participants meeting exclusion criteria for ivermectin. Permethrin will be dosed according to clinical guidelines.(35) Participants or carers will be counselled to apply the cream to the whole body from the neck down (in infants cream should also be applied to the scalp) and to leave it for 8 to 14 hours, or 4 hours in infants less than 2 months of age.

Outcome measures

All participants will undergo assessment for symptoms and signs of scabies, impetigo and other skin disease.(36) Assessment will include history questions regarding the presence of

For peer review only - http://bmjopen.bmj.com/site/about/guidelines.xhtml

itch, contact history and a simplified skin examination. Skin examination will be limited to areas that are usually exposed (arms from above elbow to fingers, legs from above knee to toes, head and neck). Other areas (including breasts, groin or genitals) will not be examined. Data suggest that in this setting a limited examination detects more than 90% cases of scabies.(36) The skin of children less than 2 years age will be examined more generally, as scabies may be more widespread in this age group.(3)

Scabies will be diagnosed according to consensus criteria established by the International Alliance for the Control of Scabies (2020 IACS criteria).(37, 38) Categorisation will be based on the identification of typical scabies lesions, typical body distribution of lesions and presence of itch and/or positive contact history. Diagnosis will therefore use levels B (Clinical Scabies) and C (Suspected Scabies) (See Table 2). Microscopy was not used as it is not feasible or practical for programmatic roll-out in these remote settings. Confirmation of diagnosis with dermatoscopy was not considered feasible as the specialist skills required exceeded the training of the local health workers.

Impetigo will be recorded if papular, pustular or ulcerative lesions surrounded by erythema, or with crusts, pus or bullae are seen.(7) This approach is consistent to diagnostic processes in previous scabies community intervention trials. Examinations will be conducted by nurses from Western Province. Nurses will receive one week of theoretical and practical training in the clinical assessment for scabies and impetigo, including application of the 2020 IACS criteria (see supplementary file 2).(39) Nurses will receive additional training prior to the follow-up surveys at 12 and 24 months.

For peer review only

Other severe skin infections such as ulcers, abscesses or suspected cases of crusted scabies will also be recorded where noted. If these, or other significant medical conditions are noted during the survey, participants will be referred off-study to the local health clinic for assessment and management.

In addition to skin examination data we will also collect information on presentations to health facilities in the study villages. Government health facilities in Solomon Islands routinely record the details of all attendances and admissions in paper-based registers. Cases of scabies, local and serious bacterial infections and other skin diseases are recorded. Facilities report aggregated data using a standardised form each month. Data is transferred electronically through the District Health Information System (DHIS2) to the MHMS Health Information Statistics Unit. We will use the information from DHIS2 to assess the number of presentations to health facilities with scabies and impetigo. Data collected in the 12 months prior to MDA will be compared to data collected in the 24 months following MDA.

For peer review only

Table 2: Case definitions for scabies using the 2020 IACS Criteria (38)

**Criteria Category**		**Used in Survey**
**Confirmed scabies**		
	At least one of:	
A1	Mites, eggs or faeces on light microscopy of skin samples	No
A2	Mites, eggs or faeces visualised on an individual using a high-powered imaging device	No
A3	Mite visualised on an individual using dermoscopy	No
**Clinical scabies**		
	At least one of:	
B1	Scabies burrows	No
B2	Typical lesions affecting male genitalia	No
B3	Typical lesions in a typical distribution and two history features*	Yes
Suspected Scabies		
	One of:	
C1	Typical lesions in a typical distribution and one history feature*	Yes
C2	Atypical lesions or atypical distribution and two history features*	Yes

**History features include (i) Itch, (ii) Positive contact history*

Diagnosis can be made at one of the three levels (A, B or C). A diagnosis of clinical or suspected scabies should only be made if other differential diagnoses are considered less likely than scabies.

Safety monitoring and reporting

Ivermectin is well tolerated and has a significant dose safety margin with no safety concerns at much higher doses than clinically required (up to 120 mg in adults, approximately 2000 μg/kg).(40, 41) Over one billion doses have been distributed for control of onchocerciasis and lymphatic filariasis with few effects reported beyond minor, reversible events.(42, 43) There have been cases of encephalopathy following ivermectin administration but these have been in the context of loiasis, a disease which has not been detected in Solomon Islands.(44) Ivermectin is on the WHO Model Essential Medicines List and the Solomon Islands Essential Medicines List for the treatment of scabies.(31, 45) Although topical benzyl benzoate is the

For peer review only - http://bmjopen.bmj.com/site/about/guidelines.xhtml

standard treatment of scabies in the Solomon Islands, topical 5% permethrin will be used in the study due to its increased efficacy and lower rate of local side effects.(46) Permethrin is well tolerated with very few side effects, including in infants.(47, 48) Nonetheless, we will record all reported adverse events related to treatment using passive monitoring.

Participants will be advised to report any adverse events to clinic nurses or directly to the study team if the adverse event occurs immediately post MDA. The clinic nurses will relay information to the study coordinator who will document the adverse event and send a report to the Principal Investigator who will in turn collate adverse events for reporting to the Data Safety Monitoring Board (DSMB). Any serious adverse events or suspected serious adverse reactions will be reported to the study coordinator by the study team, or clinical staff at the clinics and hospitals in the study area. Hospitals will be briefed on the study and provided with comprehensive reporting forms and the MDA schedule. Hospitals will report any admissions or deaths from study villages for one month following administration of the first dose of ivermectin. We will retrospectively review mortality records to ensure all deaths from study villages have been captured. We will review routinely collected summary data on all stillbirths from hospitals in the area for 12 months following MDA.

An independent DSMB will provide oversight to the safety and progress of the trial. The DSMB will meet via teleconference prior to the study, in the first three months after MDA and at the conclusion of the study. Any serious adverse events and suspected serious adverse reactions will be reported to the DSMB within seven days.

Sample size

For peer review only

Sample size calculations were based on scabies prevalence in Western Province of Solomon Islands and the effect size measured in previous studies of ivermectin-based MDA for scabies.(6, 17, 21) A standard Monte Carlo simulation method with 1000 repetitions was used to estimate the required sample size and number of villages to achieve statistical power of 80%.(49) We assumed that scabies prevalence across villages would range from 10% to 30% (mean 20%, standard deviation, SD, 5%) at baseline (6). The effect size measured in previous studies with two doses of ivermectin MDA was used to assume the prevalence of scabies 12 months after MDA will be between 3% and 9% (mean 6%, SD 2%) in the one-dose group and between 1% and 5% (mean 3%, SD 1%) in the two-dose group (17, 21). We assumed an average village size of 250 people with a range of 200 to 300.(50) We considered a non-inferiority margin of 5% (prevalence of scabies at 12 months in the one-dose group minus prevalence at 12 months in the two-dose group) to be relevant from a public health perspective. Based on these assumptions, 20 villages, randomised equally, would be sufficient to achieve the required power.

Randomisation

An independent statistician will randomise villages to the one-dose or two-dose group in a 1:1 ratio once the 20 study villages have agreed to participate. There will be no stratification within the randomisation process. There will be 10 villages in each group (Figure 3).

Analysis plan

For peer review only

For peer review only - http://bmjopen.bmj.com/site/about/guidelines.xhtml

We will account for clustering when calculating all study outcomes by calculating outcomes at the cluster level and analysing data at the cluster level and not the individual level.(51) The range of cluster-level outcomes will be reported by group.

Primary outcome

The prevalence of scabies in each village will be calculated at baseline (0 months), and 12 months. The prevalence will be calculated by dividing the number of participants with scabies by the denominator (the total number of participants examined for scabies) in each cluster. The denominator will vary at each timepoint as we will include all participants who consented for skin examination, regardless of their involvement at other timepoints.

The difference in scabies prevalence between baseline and 12 months will be calculated for each village. The means of these differences will be calculated in the two treatment groups and compared by calculating the difference between the means. If the upper limit of the two-sided 95% confidence interval of the mean difference between the two study groups is less than or equal to 5% (the clinically relevant non-inferiority margin) the one-dose regimen will be considered non-inferior.

Secondary outcomes

The analysis for the prevalence of scabies at 24 months and impetigo at 12 and 24 months will be done in the same way as for the primary endpoint.

The change in the number of presentations to health facilities for scabies and impetigo will be analysed in three ways. First, the total number of presentations in the 12 months before MDA will be compared to the number of presentations in months 1 to 12 and 13 to 24 after MDA.

For peer review only - http://bmjopen.bmj.com/site/about/guidelines.xhtml

Second, we will calculate the proportion of clinic presentations for scabies and impetigo by dividing the number of presenations for scabies and impetigo by the total number of clinic presentations for any condition. We will calculate this proportion for the 12 months before MDA, 1 to 12, and 13 to 24 months after MDA. Calculating the proportion will account for any changes in population size or operational status of health facilities. Third, we will compare the number of clinic presentations for scabies and impetigo in the clinics that service the study villages and compare this with clinic presentation for scabies and impetigo in other clinics in the province, this will be adjusted for population size.

The number of adverse events in each study group will be calculated as a proportion of the total number of participants in each study group that received MDA at baseline. We will also report the number of deaths in the month following MDA in each study group as a proportion of the number of participants in each group.

Data collection and management

Data will be collected using a combination of paper-based and electronic forms. Paper forms will be stored in locked filing cabinets. Information will be deidentified and participant names will only be recorded on consent forms. Only authorised study staff will be able to access forms. Data will be destroyed after 15 years in compliance with local guidelines. Skin examination data will be collected and managed using REDCap electronic data capture tools hosted at Murdoch Children's Research Institute.(52, 53) REDCap is a secure, web-based software platform designed to support data capture for research studies.

For peer review only

Trial Status

Baseline data collection and MDA took place between May and July 2019. A total of 5,260 participants were enrolled. Follow-up village data collection is scheduled to take place between May and July 2020 and between May and July 2021.

Ethics and dissemination

The RISE trial is investigator-initiated and funded by the National Health and Medical Research Council of Australia (GNT1127297). The funder was not involved in protocol development or the study process including site selection, management, data collection or analysis of the results. The trial is a collaboration between the Murdoch Children's Research Institute, the Solomon Islands MHMS, the Kirby Institute at the University of New South Wales, the London School of Hygiene and Tropical Medicine and the Australian National University.

The trial was designed in accordance with CONSORT guidelines and our reporting of the protocol conforms to the Standard Protocol Items: Recommendations for Interventional Trials (SPIRIT) 2013 checklist.(54-56) This trial has been approved by the Solomon Islands Health Research and Ethics Review Board (HRE005/18) and Royal Children's Hospital Human Research Ethics Committee (38099A) in Melbourne, Australia and will be conducted in accordance with Good Clinical Practice.

De-identified data may be made available for further analysis with appropriate approvals. Results of the study will be presented locally and made available to health policy decision

For peer review only

makers and clinical staff. Villages that participated in the trial will be presented the results in a culturally appropriate way that is easy to understand and interpret. Participants will have the opportunity for results to be explained to them in their own language through a series of village meetings as well as printed information leaflets.

For peer review only

DISCUSSION

Scabies is a common disease in many tropical and low-income settings and has been prioritised for control by WHO, but there are still gaps in knowledge to determine the optimum approach to control in settings where scabies is highly endemic.(9) The results of this trial will have an impact on national, regional and global strategies for scabies control.

Island communities in the Pacific have among the highest global prevalence of scabies and understanding how to implement MDA in these settings has the potential for translation into huge public health impact for these communities.(5) However, the results may not be generalisable to populations with a much lower prevalence of the disease, to settings with higher population density, or to urban settings. The non-inferiority margin of 5% was determined using available evidence but may not represent the appropriate level of public health significance in all circumstances. A greater or lesser margin may be considered non-inferior in other settings, depending on factors including baseline disease prevalence, number of rounds planned, costs of implementing each regimen and available resources. This trial is designed to assess a single round of MDA, there is scope for further research to assess the efficacy of repeated annual rounds of MDA. The cluster-randomised design will allow analysis of the impact of MDA at the community level. We will be able to assess the impact of the intervention on the whole community, even for those who will not receive MDA.

If the RISE trial finds that one-dose ivermectin MDA is inferior, then the need for two doses of ivermectin-based MDA would need to be taken into account in decision-making around control strategies for scabies. It would also provide impetus for further research to identify new treatments for scabies that may be able to be implemented with one dose. Approaches

For peer review only - http://bmjopen.bmj.com/site/about/guidelines.xhtml

may include novel treatments that are ovicidal, or other medications with a longer half-life, such as moxidectin(57). If one-dose ivermectin-based MDA is found to be non-inferior to two-dose then this strategy will be highly attractive for implementation as a public health program. The lower cost, simplified logistics and ability to integrate with other programs would make scabies control programs more feasible in low-income settings.

For peer review only

Funding

This research is funded by the National Health and Medical Research Council of Australia (NHMRC) (APP1127297). DE, LR, JK and ACS are supported by fellowships from the NHMRC. MM is supported by the UK National Insitute for Health Research.

Acknowledgements

The authors are grateful to the Solomon Islands MHMS and the communities who will participate. We appreciate the support and contributions of Pauline McNeil, Nemia Bainivalu, Gregory Jilini, Michael Larui, Ivan Ghemu, Freda Pitakaka, William Horoto, Jeffrey Korini, Soraya Pina, Yvonne Tuni, Selina Maena, Frederick Neqo, Susana Vaz Nery and Naomi Clarke. The RISE study team includes: Sana Bisili, Aisling Byrne, Sharmillah Jack, Arthur Keremama, Alam Khatak, Erica Lazu, Relinta Manaka, Davis Pesala, Deanne Seppy, Winter Sino, Patson Solomon, Stephen Tiazi, and Salote Wickham.

This study is dedicated to Dr Tenneth Dalipanda, former Permanent Secretary of the Solomon Islands MHMS. Dr Tenneth was committed to improving the health of Solomon Islanders. He was an advocate for public health research and without his support we would not have been able to conduct this research and many studies before it.

For peer review only

Author contributions

Study concept and design were conducted by the investigators: ACS, DE, JMK, LR, MJW, MM, RA, TS, OS and TN. Critical revision of concept and design and intellectual input in the study protocol was done by all authors: SJL, SLP, DE, OS, TN, DB, CG, TS, ACG, MHO, RA, MM, MJW, LR, JK and ACS. Drafting of the protocol was done by SLP and ACS, with review by all authors. Drafting of the manuscript was done by SJL. Critical revision of the manuscript was performed by all authors. Study supervision is conducted by the investigators.

Competing interests

None declared

Word count:

4413

For peer review only

REFERENCES

1. Vos T, Abajobir AA, Abate KH, Abbafati C, Abbas KM, Abd-Allah F, et al. Global, regional, and national incidence, prevalence, and years lived with disability for 328 diseases and injuries for 195 countries, 1990–2016: a systematic analysis for the Global Burden of Disease Study 2016. The Lancet. 2017;390(10100):1211-59.
2. Karimkhani C, Colombara DV, Drucker AM, Norton SA, Hay R, Engelman D, et al. The global burden of scabies: a cross-sectional analysis from the Global Burden of Disease Study 2015. The Lancet Infectious Diseases. 2017;17(12):1247-54.
3. Hardy M, Engelman D, Steer A. Scabies: A clinical update. Australian Family Physician. 2017;46(5):264-8.
4. Mellanby K. Transmission of Scabies. BMJ. 1941;2(4211):405-6.
5. Romani L, Steer AC, Whitfeld MJ, Kaldor JM. Prevalence of scabies and impetigo worldwide: a systematic review. Lancet Infect Dis. 2015;15(8):960-7.
6. Mason DS, Marks M, Sokana O, Solomon AW, Mabey DC, Romani L, et al. The Prevalence of Scabies and Impetigo in the Solomon Islands: A Population-Based Survey. PLoS Neglected Tropical Diseases [electronic resource]. 2016;10(6):e0004803.
7. Romani L, Koroivueta J, Steer AC, Kama M, Kaldor JM, Wand H, et al. Scabies and impetigo prevalence and risk factors in Fiji: a national survey. PLoS Negl Trop Dis. 2015;9(3):e0003452.
8. Aung PTZ, Cuningham W, Hwang K, Andrews RM, Carapetis JR, Kearns T, et al. Scabies and risk of skin sores in remote Australian Aboriginal communities: A self-controlled case series study. PLoS Negl Trop Dis. 2018;12(7):e0006668.
9. Engelman D, Cantey PT, Marks M, Solomon AW, Chang AY, Chosidow O, et al. The public health control of scabies: priorities for research and action. Lancet. 2019;394(10192):81-92.
10. Parks T, Smeesters PR, Steer AC. Streptococcal skin infection and rheumatic heart disease. Curr Opin Infect Dis. 2012;25(2):145-53.
11. Thornley S, Marshall R, Jarrett P, Sundborn G, Reynolds E, Schofield G. Scabies is strongly associated with acute rheumatic fever in a cohort study of Auckland children. J Paediatr Child Health. 2018;54(6):625-32.
12. Tasani M, Tong SY, Andrews RM, Holt DC, Currie BJ, Carapetis JR, et al. The Importance of Scabies Coinfection in the Treatment Considerations for Impetigo. Pediatr Infect Dis J. 2016;35(4):374-8.
13. eTG complete. [Internet]. Melbourne, Victoria: Therapeutic Guidelines Ltd. Available from: https://tgldcdp.tg.org.au.acs.hcn.com.au/viewTopic?topicfile=insects-mites&guidelineName=Dermatology#toc_d1e110.
14. WHO Model Formulary for Children. Geneva, Switzerland: World Health Organisation; 2010.
15. David, Lucinda. A Review of Scabies: An Infestation More than Skin Deep. Dermatology. 2019;235(2):79-90.
16. Engelman D, Steer A. Control Strategies for Scabies. Tropical Medicine and Infectious Disease. 2018;3(3):98.
17. Romani L, Marks M, Sokana O, Nasi T, Kamoriki B, Cordell B, et al. Efficacy of mass drug administration with ivermectin for control of scabies and impetigo, with coadministration of azithromycin: a single-arm community intervention trial. The Lancet Infectious Diseases. 2019;19(5):510-8.
18. Marks M, Taotao-Wini B, Satorara L, Engelman D, Nasi T, Mabey DC, et al. Long term control of scabies fifteen years after an intensive treatment programme. PLoS Negl Trop Dis. 2015;9(12):e0004246.
19. Taplin D, Porcelain SL, Meinking TL, Athey RL, Chen JA, Castillero PM, et al. Community control of scabies: a model based on use of permethrin cream. Lancet. 1991;337(8748):1016-8.

For peer review only

20. Bockarie MJ, Alexander ND, Kazura JW, Bockarie F, Griffin L, Alpers MP. Treatment with ivermectin reduces the high prevalence of scabies in a village in Papua New Guinea. Acta Tropica. 2000;75(1):127-30.
21. Romani L, Whitfeld MJ, Koroivueta J, Kama M, Wand H, Tikoduadua L, et al. Mass Drug Administration for Scabies Control in a Population with Endemic Disease. New England Journal of Medicine. 2015;373(24):2305-13.
22. Hotez P. Mass Drug Administration and Integrated Control for the World's High-Prevalence Neglected Tropical Diseases. Clinical Pharmacology & Therapeutics. 2009;85(6):659-64.
23. Romani L, Marks M, Sokana O, Nasi T, Kamoriki B, Wand H, et al. Feasibility and safety of mass drug coadministration with azithromycin and ivermectin for the control of neglected tropical diseases: a single-arm intervention trial. The Lancet Global Health. 2018;6(10):e1132-e8.
24. Bernigaud C, Samarawickrama GR, Jones MK, Gasser RB, Fischer K. The Challenge of Developing a Single-Dose Treatment for Scabies. Trends in Parasitology. 2019;35(11):931-43.
25. Report of the Tenth Meeting of the WHO Strategic and Technical Advisory Group for Neglected Tropical Diseases. World Health Organisation; 2017.
26. Rosumeck S, Nast A, Dressler C. Ivermectin and permethrin for treating scabies. Cochrane Database of Systematic Reviews. 2018;4:CD012994.
27. Mohammed KA, Deb RM, Stanton MC, Molyneux DH. Soil transmitted helminths and scabies in Zanzibar, Tanzania following mass drug administration for lymphatic filariasis--a rapid assessment methodology to assess impact. Parasites & Vectors [Electronic Resource]. 2012;5:299.
28. Martin D, Wiegand R, Goodhew B, Lammie P, Mkocha H, Kasubi M. Impact of Ivermectin Mass Drug Administration for Lymphatic Filariasis on Scabies in Eight Villages in Kongwa District, Tanzania. The American Journal of Tropical Medicine and Hygiene. 2018;99(4):937-9.
29. Hodge N, Slatyer B, Skiller L. Solomon Islands Health System Review. Whittaker M, editor. Geneva, Switzerland: World Health Organisation; 2015.
30. Human Development Indices and Indicators: 2018 Statistical Update. Washington DC, USA: United Nations Development Programme; 2018.
31. World Health Organisation Model List of Essential Medicines, 21st List. Geneva: World Health Organisation; 2019.
32. Lawrence J, Sodahlon YK, Ogoussan KT, Hopkins AD. Growth, Challenges, and Solutions over 25 Years of Mectizan and the Impact on Onchocerciasis Control. PLOS Neglected Tropical Diseases. 2015;9(5):e0003507.
33. Alexander ND, Cousens SN, Yahaya H, Abiose A, Jones BR. Ivermectin dose assessment without weighing scales. Bulletin of the World Health Organization. 1993;71(3-4):361-6.
34. Stunkard AJ, Sørensen T, Schulsinger F. Use of the Danish Adoption Register for the study of obesity and thinness. Research publications - Association for Research in Nervous and Mental Disease. 1983;60:115-20.
35. Australian Medicines Handbook [Internet]. Available from: https://amhonline.amh.net.au.acs.hcn.com.au/chapters/dermatological-drugs/scabicides-pediculicides/permethrin.
36. Marks M, Engelman D, Romani L, Mason D, Sokana O, Kama M, et al. Exploration of a simplified clinical examination for scabies to support public health decision-making. PLoS Neglected Tropical Diseases [electronic resource]. 2018;12(12):e0006996.
37. Engelman D, Fuller LC, Steer AC, for the International Alliance for the Control of Scabies Delphi p. Consensus criteria for the diagnosis of scabies: A Delphi study of international experts. PLOS Neglected Tropical Diseases. 2018;12(5):e0006549.
38. Engelman D, Yoshizumi J, Hay RJ, Osti M, Micali G, Norton S, et al. The 2020 IACS Consensus Criteria for the Diagnosis of Scabies. British Journal of Dermatology. 2020.
39. Osti MH, Sokana O, Gorae C, Whitfeld MJ, Steer AC, Engelman D. The diagnosis of scabies by non-expert examiners: A study of diagnostic accuracy. PLOS Neglected Tropical Diseases. 2019;13(8):e0007635.

For peer review only

40. Australian Public Assessment Report for Ivermectin. Woden, ACT, Australia: Commonwealth of Australia; 2013.
41. Guzzo CA, Furtek CI, Porras AG, Chen C, Tipping R, Clineschmidt CM, et al. Safety, Tolerability, and Pharmacokinetics of Escalating High Doses of Ivermectin in Healthy Adult Subjects. The Journal of Clinical Pharmacology. 2002;42(10):1122-33.
42. Ottesen EA, Hooper PJ, Bradley M, Biswas G. The Global Programme to Eliminate Lymphatic Filariasis: Health Impact after 8 Years. PLoS Neglected Tropical Diseases. 2008;2(10):e317.
43. Tielsch JM, Beeche A. Impact of ivermectin on illness and disability associated with onchocerciasis. Tropical Medicine & International Health. 2004;9(4):A45-A56.
44. Gardon J, Gardon-Wendel N, Demanga N, Kamgno J, Chippaux J-P, Boussinesq M. Serious reactions after mass treatment of onchocerciasis with ivermectin in an area endemic for Loa loa infection. The Lancet. 1997;350(9070):18-22.
45. Solomon Islands Essential Medicines List. Honiara, Solomon Islands: Ministry of Health and Medical Services; 2017.
46. Strong M, Johnstone P. Interventions for treating scabies. The Cochrane database of systematic reviews. 2007;2007(3):CD000320-CD.
47. Currie BJ, McCarthy JS. Permethrin and ivermectin for scabies. New England Journal of Medicine. 2010;362(8):717-25.
48. Hoffmann JC, Mossner R, Schon MP, Lippert U. Topical scabies therapy with permethrin is effective and well tolerated in infants younger than two months. J Dtsch Dermatol Ges. 2019;17(6):597-600.
49. Landau S, Stahl D. Sample size and power calculations for medical studies by simulation when closed form expressions are not available. Statistical Methods in Medical Research. 2013;22(3):324-45.
50. Provincial Profile of the 2009 Population & Housing Census: Western, Solomon Islands. 2009.
51. Hayes RJ, Moulton LH. Cluster randomised trials, second edition: CRC Press; 2017. 424 p.
52. Harris PA, Taylor R, Minor BL, Elliott V, Fernandez M, O'Neal L, et al. The REDCap consortium: Building an international community of software platform partners. Journal of Biomedical Informatics. 2019;95:103208.
53. Harris PA, Taylor R, Thielke R, Payne J, Gonzalez N, Conde JG. Research electronic data capture (REDCap)—A metadata-driven methodology and workflow process for providing translational research informatics support. Journal of Biomedical Informatics. 2009;42(2):377-81.
54. CONSORT. CONSORT Transparent Reporting of Trials [updated 2020.]. Available from: http://www.consort-statement.org/.
55. SPIRIT. SPIRIT Statement [updated 2020. Available from: https://www.spirit-statement.org/.
56. Chan A-W, Tetzlaff JM, Altman DG, Laupacis A, Gøtzsche PC, Krleža-Jerić K, et al. SPIRIT 2013 statement: defining standard protocol items for clinical trials. Ann Intern Med. 2013;158(3):200-7.
57. Mounsey KE, Bernigaud C, Chosidow O, McCarthy JS. Prospects for Moxidectin as a New Oral Treatment for Human Scabies. PLoS Neglected Tropical Diseases [electronic resource]. 2016;10(3):e0004389.

For peer review only

Figure legends/captions:

Figure 1. Study location in Solomon Islands

Figure 2. Study location in Western Province, Solomon Islands

Figure 3. Study flow diagram

Supplementary material

Skin examination training for nurses

For peer review only

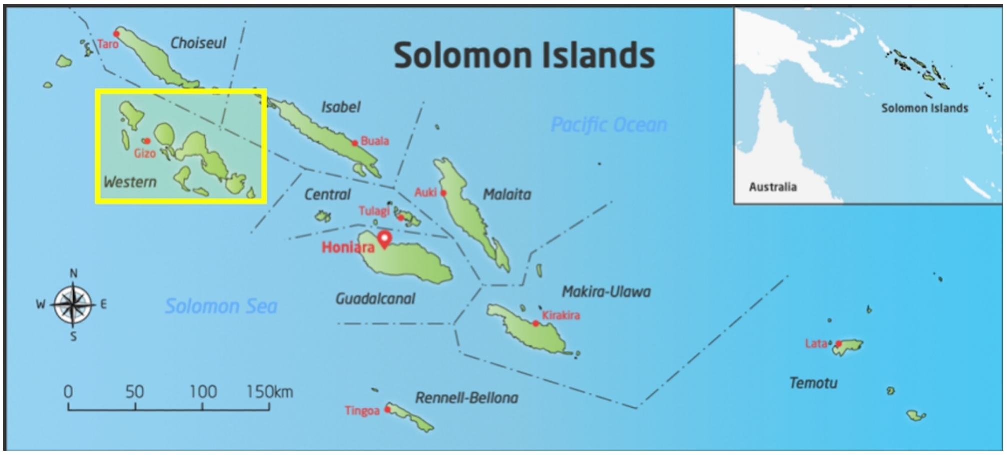

Figure 1. Study location in Solomon Islands

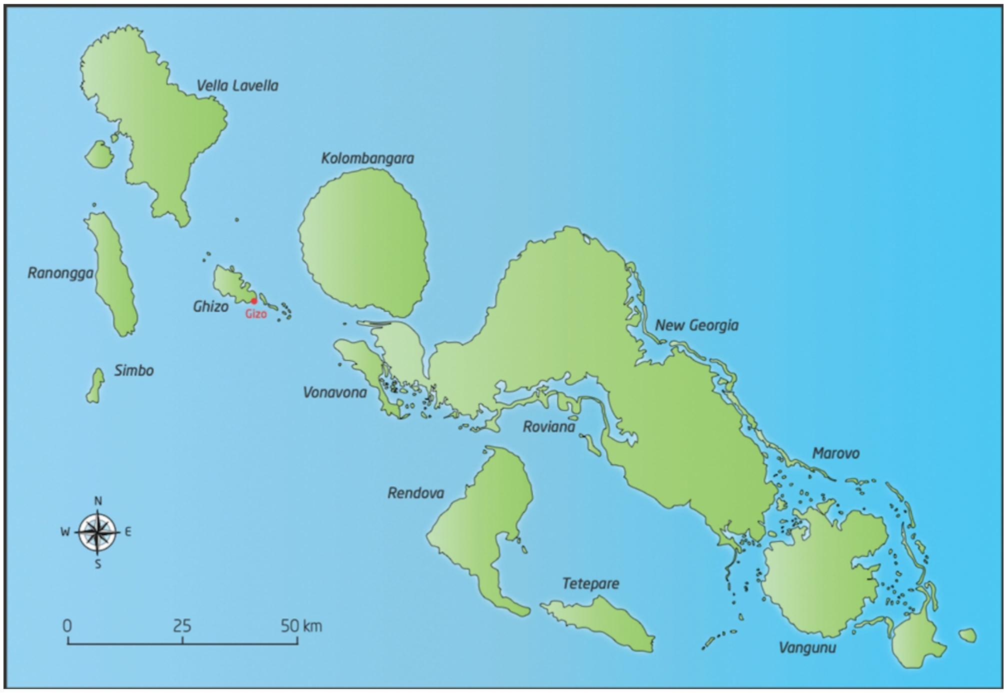

Figure 2. Study location in Western Province, Solomon Islands

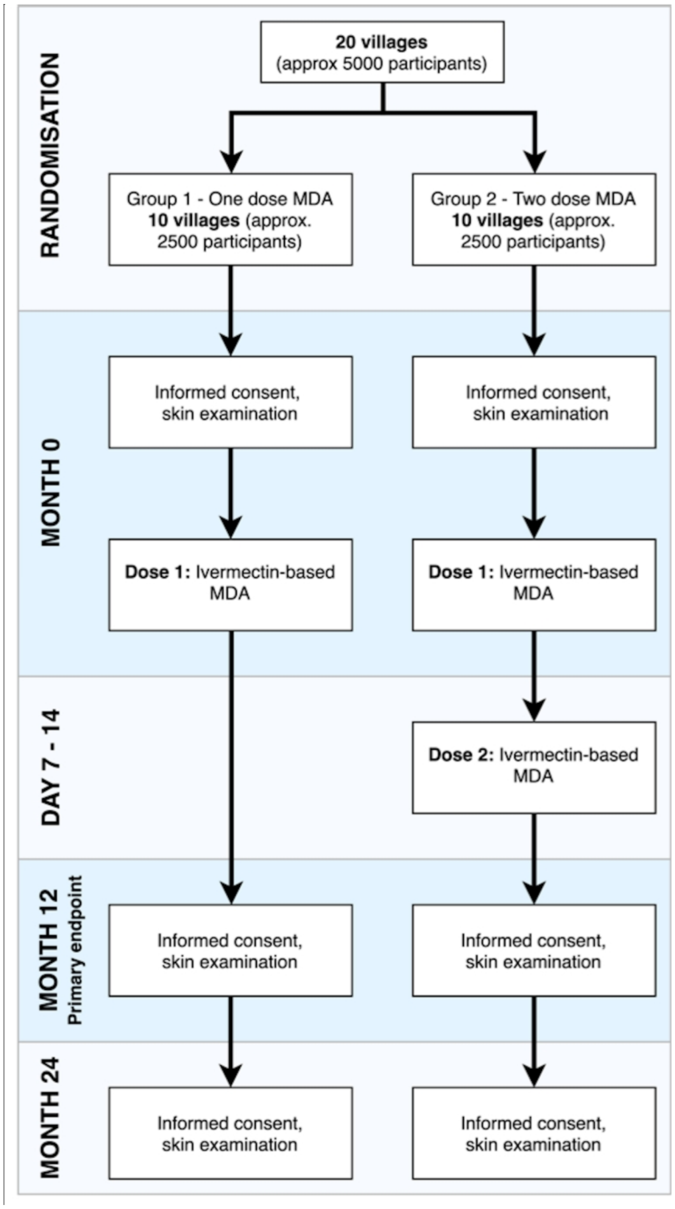

Figure 3. Study flow diagram

Participant Information Statement and Consent Form

Initial Visit

HREC Project Number: 38099A

Research Project Title: RISE: Regimens of Ivermectin for Scabies Elimination

Local Principal Investigator: Mr Oliver Sokana

Version Number: 4

Version Date: 25/02/19

Thank you for taking the time to read this **Participant Information Statement and Consent Form**. We would like to invite you to participate in a research project that is explained below.

This document is 5 pages long. Please make sure you have all the pages.

What is an Information Statement and Consent Form?

An Information and Consent Form tells you about the research project. It clearly explains exactly what the research project will involve. This information is to help you decide whether or not you would like to take part in the research. Please read it carefully.

Before you decide if you want to take part or not, you can ask us any questions you have about the project. You may want to talk about the project with your family, friends or health care worker.

Taking part in the research is up to you

It is your choice whether or not you take part in the research. You do not have to agree if you do not want to. If you decide you do not want to take part, it will not affect the treatment and care you get.

Signing the form

If you want to take part in the research, please sign the consent form at the end of this document. By signing the form you are telling us that you:

- Understand what you have read
- Have had a chance to ask questions and received satisfactory answers
- Consent to taking part in the project. We will give you a copy of this form to keep.

For peer review only - http://bmjopen.bmj.com/site/about/guidelines.xhtml

1. What is the research project about?

The main aim of this project is to get rid of the skin infection known as scabies. People often don't know that they have this infection. To get the best results we try to treat everyone to make sure we don't miss anyone with infection. Usually you need to take the same medicine twice to get rid of this infection, but you may only need to take it once. We are trying to figure out if we can get rid of scabies if everyone in your community takes this medicine once.

The medicine that is used to treat scabies can also treat some kinds of intestinal worms. We are also trying to figure out whether treatment of intestinal worms is better with two doses of the medicine, compared to one dose.

2. Who is funding this research project?

The project is organised by the Solomon Islands Ministry of Health, the Murdoch Children's Research Institute, the London School of Hygiene & Tropical Medicine, The Kirby Institute and the Australian National University.

3. Why am I being asked to take part?

Twenty villages in the Western Province of the Solomon Islands have been selected at random to have their skin checked, give a sample of their stool to look for intestinal worms, and be provided with medication for scabies. Everyone living in the village will be asked to participate.

4. What do I need to do in this research project?

If you agree to take part, we will record information about your age and gender and will take your height and weight. We will examine your skin for signs of scabies and other skin problems.

Photographs may be taken of any skin lesions or rashes. These photos will not include your face or head and will not be recognisable as belonging to you. The research team will check with you before taking any photographs. If you do not wish to have a photograph taken that is fine.

We will ask you some questions about how scabies affects you and your family, and some questions about your risk of intestinal worm infections.

If you are willing to provide a stool sample, we will send your stool sample (without your name on it) to a laboratory at Murdoch Children's Research Institute in Australia to test for intestinal worm infections.

We will ask you if you are willing to take the treatment for scabies. If you agree, we will provide this treatment. For most people in the study this will be a tablet called ivermectin. For some people who can't have ivermectin, including children less than 90cm in height and pregnant women, treatment will be with a cream called permethrin. This cream only treats scabies, not intestinal worm infections.

For peer review only - http://bmjopen.bmj.com/site/about/guidelines.xhtml

If you live in a community that has been allocated two doses of treatment for scabies, we will visit you again to provide this second dose one or two weeks after the first dose.

We will visit you again to examine your skin for scabies and collect stool samples 12 months after our first visit, and then again 12 months after that. This is so we can check how effective the treatment is.

5. Can I withdraw from the project?

You can stop taking part in the project at any time. You just need to tell us so. You do not need to tell us the reason why. If you leave the project we will use any information already collected unless you tell us not to.

6. What are the possible benefits for me and other people in the future?

You and your community will receive treatment for scabies. If our study shows that this is an effective strategy, we may be able to provide this treatment to help other people in the Solomon Islands and many other countries.

7. What are the possible risks, side-effects, discomforts and/or inconveniences?

Treatment for scabies is very effective and side effects are uncommon and quickly go away.

Ivermectin occasionally causes dizziness or tummy upset. Children less than 90cm in height and pregnant women should not take ivermectin and will be offered a cream instead.

Permethrin cream occasionally causes itch and stinging.

Having your skin examined for scabies is not uncomfortable or painful. The whole process, including asking questions and examination should take less than 10 minutes.

You have previously been informed about stool collection procedures, at the time of receiving the stool collection kit.

8. What will be done to make sure my information is confidential?

Any information we collect for this project that can identify you will be treated confidentially, except as required by law. Nothing that could reveal your identity will be disclosed outside the project.

9. Will I be informed of the results when the research project is finished?

Results of the project will help us understand the best way to treat scabies in communities in the Solomon Islands and elsewhere. Results will be published in the medical literature, and a report summarizing the results will be sent to your community health worker who will pass on the

For peer review only - http://bmjopen.bmj.com/site/about/guidelines.xhtml

information to you. You and your family will not be personally identified in any report or publication.

10. Who should I contact for more information?

If you would like more information about the project, please contact:

Name: Mr Oliver Sokana

Contact telephone: 769 1615

Email: sokanao@moh.gov.sb

OR

Name: Prof. Andrew Steer

Contact telephone: +61 (3) 9345 5522

Email: Andrew.Steer@rch.org.au

If you:

- Have any concerns or complaints about the project
- Are worried about your rights as a research participant
- Would like to speak to someone independent of the project.

You can contact the Solomon Islands National Health Research Ethics Committee by telephone on (+677) 37295, or you can contact the Director of Research Ethics & Governance at The Royal Children's Hospital Melbourne by telephone on +61 (3) 9345 5044.

For peer review only - http://bmjopen.bmj.com/site/about/guidelines.xhtml

CONSENT FORM

HREC Project Number: 38099A

Research Project Title: RISE: Regimens of Ivermectin for Scabies Elimination

Local Principal Investigator: Mr Oliver Sokana

Version Number: 4

Version Date: 25/02/2019

- I have read this information statement and I understand its contents.
- I understand I have to do to be involved in this project.
- I understand the risks I could face because of my involvement in this project.
- I voluntarily consent to take part in this research project.
- I have had an opportunity to ask questions about the project and I am satisfied with the answers I have received.
- I understand that this project has been approved by The Solomon Islands National Health Research Ethics Committee and the Royal Children's Hospital Melbourne Human Research Ethics Committee. I understand that the project and any updates will be carried out in line with the National Statement on Ethical Conduct in Human Research (2007).
- I understand I will receive a copy of this Information Statement and Consent Form.

I consent/give consent for ______________________ to take part in this study

Participant Name

☐ I consent/give consent for stool sample to be analysed for intestinal worm infections *(tick box if applicable)*

Participant Signature or Fingerprint	Date	
Name of Witness to Participant's Signature	Witness Signature	Date

Declaration by researcher: I have explained the project to the participant who has signed above. I believe that they understand the purpose, extent and possible risks of their involvement in this project.

Research Team Member Name	Research Team Member Signature	Date

Note: All parties signing the Consent Form must date their own signature.

For peer review only - http://bmjopen.bmj.com/site/about/guidelines.xhtml

SUPPLEMENTARY MATERIAL

Skin examination training for nurses

Training was delivered by two Australians doctors with experience in scabies and other tropical skin conditions. Training materials were developed based on material previously delivered in the Solomon Islands and Fiji (Table S1). The examination and history component of the training was focused on identifying the relevant features required for the diagnosis of scabies and impetigo. Other differential skin diagnoses that were relevant to the setting were also included.

Training consisted of two stages; classroom training and practical training at a primary school. The classroom training content included background information on the importance of scabies as a public health problem in the Solomon Islands, further context for the study, details on global and local prevalence of scabies, complications of the diseases and basic treatment concepts. Training on the diagnosis of scabies was based on the 2020 International Alliance for the Control of Scabies Consensus Criteria for the Diagnosis of Scabies.(1)

Clinical examination was limited to exposed areas of skin – particularly the arms from the mid-upper arm to the finger tips, and the legs from the mid-upper though to the toes. A brief history component containing questions about itch and contact history was incorporated. Terminology and definitions used in training were consistent with the World Health Organization 2018 training guide, "Recognizing neglected tropical diseases through changes on the skin".(2)

For peer review only

For peer review only - http://bmjopen.bmj.com/site/about/guidelines.xhtml

Table S1 – Overview of training

**Part 1: Classroom training**
1.1 Scabies
What is scabies?
How do people get scabies?
How common is scabies?
What problems do scabies cause?
How can scabies be treated?
How can we get rid of scabies in the community?
1.2 Approach to diagnosis
About the skin
Dermatological terms
IACS criteria
History taking
Examination
Differential diagnoses
1.3 Facilitated practice with clinical images
**Part 2: Supervised field training**
2.1 Practice examination
**Part 3: Assessment**
3.1 Slide assessment
3.2 Field assessment

For peer review only

REFERENCES

1. Engelman D, Yoshizumi J, Hay RJ, Osti M, Micali G, Norton S, et al. The 2020 IACS Consensus Criteria for the Diagnosis of Scabies. British Journal of Dermatology. 2020.

2. World Health O. Recognizing neglected tropical diseases through changes on the skin: a training guide for front-line health workers. Geneva: World Health Organization; 2018 2018.

For peer review only

Reporting checklist for protocol of a clinical trial.

Based on the SPIRIT guidelines.

Instructions to authors

Complete this checklist by entering the page numbers from your manuscript where readers will find each of the items listed below.

Your article may not currently address all the items on the checklist. Please modify your text to include the missing information. If you are certain that an item does not apply, please write "n/a" and provide a short explanation.

Upload your completed checklist as an extra file when you submit to a journal.

In your methods section, say that you used the SPIRITreporting guidelines, and cite them as:

Chan A-W, Tetzlaff JM, Altman DG, Laupacis A, Gøtzsche PC, Krleža-Jerić K, Hróbjartsson A, Mann H, Dickersin K, Berlin J, Doré C, Parulekar W, Summerskill W, Groves T, Schulz K, Sox H, Rockhold FW, Rennie D, Moher D. SPIRIT 2013 Statement: Defining standard protocol items for clinical trials. Ann Intern Med. 2013;158(3):200-207

For peer review only

		Reporting Item	Page Number
**Administrative information**			
Title	#1	Descriptive title identifying the study design, population, interventions, and, if applicable, trial acronym	1
Trial registration	#2a	Trial identifier and registry name. If not yet registered, name of intended registry	4
Trial registration: data set	#2b	All items from the World Health Organization Trial Registration Data Set	1, 4, 24
Protocol version	#3	Date and version identifier	N/A V4 25/2/19
Funding	#4	Sources and types of financial, material, and other support	24

Roles and responsibilities: contributorship	#5a	Names, affiliations, and roles of protocol contributors	1
Roles and responsibilities: sponsor contact information	#5b	Name and contact information for the trial sponsor	1, 2
Roles and responsibilities: sponsor and funder	#5c	Role of study sponsor and funders, if any, in study design; collection, management, analysis, and interpretation of data; writing of the report; and the decision to submit the report for publication, including whether they will have ultimate authority over any of these activities	24
Roles and responsibilities: committees	#5d	Composition, roles, and responsibilities of the coordinating centre, steering committee, endpoint adjudication committee, data management team, and other individuals or groups overseeing the trial, if applicable (see Item 21a for data monitoring committee)	1, 24-25
**Introduction**			
Background and rationale	#6a	Description of research question and justification for undertaking the trial, including summary of relevant studies (published and unpublished) examining benefits and harms for each intervention	6-8
Background and rationale: choice of comparators	#6b	Explanation for choice of comparators	9
Objectives	#7	Specific objectives or hypotheses	10
Trial design	#8	Description of trial design including type of trial (eg, parallel group, crossover, factorial, single group), allocation ratio, and framework (eg, superiority, equivalence, non-inferiority, exploratory)	9
**Methods: Participants,**			

For peer review only

**interventions, and outcomes**			
Study setting	#9	Description of study settings (eg, community clinic, academic hospital) and list of countries where data will be collected. Reference to where list of study sites can be obtained	11
Eligibility criteria	#10	Inclusion and exclusion criteria for participants. If applicable, eligibility criteria for study centres and individuals who will perform the interventions (eg, surgeons, psychotherapists)	11-12
Interventions: description	#11a	Interventions for each group with sufficient detail to allow replication, including how and when they will be administered	13-14
Interventions: modifications	#11b	Criteria for discontinuing or modifying allocated interventions for a given trial participant (eg, drug dose change in response to harms, participant request, or improving / worsening disease)	16
Interventions: adherance	#11c	Strategies to improve adherence to intervention protocols, and any procedures for monitoring adherence (eg, drug tablet return; laboratory tests)	13-14
Interventions: concomitant care	#11d	Relevant concomitant care and interventions that are permitted or prohibited during the trial	12
Outcomes	#12	Primary, secondary, and other outcomes, including the specific measurement variable (eg, systolic blood pressure), analysis metric (eg, change from baseline, final value, time to event), method of aggregation (eg, median, proportion), and time point for each outcome. Explanation of the clinical relevance of chosen efficacy and harm outcomes is strongly recommended	14-16
Participant timeline	#13	Time schedule of enrolment, interventions (including any run-ins and washouts), assessments, and visits for participants. A schematic diagram is highly recommended (see Figure)	13-15, Fig. 3
Sample size	#14	Estimated number of participants needed to achieve study objectives and how it was determined, including	17-18

For peer review only

		clinical and statistical assumptions supporting any sample size calculations	
Recruitment	#15	Strategies for achieving adequate participant enrolment to reach target sample size	12-13
**Methods: Assignment of interventions (for controlled trials)**			
Allocation: sequence generation	#16a	Method of generating the allocation sequence (eg, computer-generated random numbers), and list of any factors for stratification. To reduce predictability of a random sequence, details of any planned restriction (eg, blocking) should be provided in a separate document that is unavailable to those who enrol participants or assign interventions	18
Allocation concealment mechanism	#16b	Mechanism of implementing the allocation sequence (eg, central telephone; sequentially numbered, opaque, sealed envelopes), describing any steps to conceal the sequence until interventions are assigned	N/A unblinded study
Allocation: implementation	#16c	Who will generate the allocation sequence, who will enrol participants, and who will assign participants to interventions	18
Blinding (masking)	#17a	Who will be blinded after assignment to interventions (eg, trial participants, care providers, outcome assessors, data analysts), and how	9
Blinding (masking): emergency unblinding	#17b	If blinded, circumstances under which unblinding is permissible, and procedure for revealing a participant's allocated intervention during the trial	N/A unblinded study
**Methods: Data collection, management, and analysis**			
Data collection plan	#18a	Plans for assessment and collection of outcome, baseline, and other trial data, including any related processes to promote data quality (eg, duplicate	14-16

For peer review only

		measurements, training of assessors) and a description of study instruments (eg, questionnaires, laboratory tests) along with their reliability and validity, if known. Reference to where data collection forms can be found, if not in the protocol	
Data collection plan: retention	#18b	Plans to promote participant retention and complete follow-up, including list of any outcome data to be collected for participants who discontinue or deviate from intervention protocols	12-13
Data management	#19	Plans for data entry, coding, security, and storage, including any related processes to promote data quality (eg, double data entry; range checks for data values). Reference to where details of data management procedures can be found, if not in the protocol	20
Statistics: outcomes	#20a	Statistical methods for analysing primary and secondary outcomes. Reference to where other details of the statistical analysis plan can be found, if not in the protocol	18-19
Statistics: additional analyses	#20b	Methods for any additional analyses (eg, subgroup and adjusted analyses)	18-19
Statistics: analysis population and missing data	#20c	Definition of analysis population relating to protocol non-adherence (eg, as randomised analysis), and any statistical methods to handle missing data (eg, multiple imputation)	18-19
**Methods: Monitoring**			
Data monitoring: formal committee	#21a	Composition of data monitoring committee (DMC); summary of its role and reporting structure; statement of whether it is independent from the sponsor and competing interests; and reference to where further details about its charter can be found, if not in the protocol. Alternatively, an explanation of why a DMC is not needed	16-17
Data monitoring: interim analysis	#21b	Description of any interim analyses and stopping guidelines, including who will have access to these	N/A Single intervention. DSMB

For peer review only

		interim results and make the final decision to terminate the trial	and Principal Investigator can stop the study.
Harms	#22	Plans for collecting, assessing, reporting, and managing solicited and spontaneously reported adverse events and other unintended effects of trial interventions or trial conduct	16-17
Auditing	#23	Frequency and procedures for auditing trial conduct, if any, and whether the process will be independent from investigators and the sponsor	17
**Ethics and dissemination**			
Research ethics approval	#24	Plans for seeking research ethics committee / institutional review board (REC / IRB) approval	20-21
Protocol amendments	#25	Plans for communicating important protocol modifications (eg, changes to eligibility criteria, outcomes, analyses) to relevant parties (eg, investigators, REC / IRBs, trial participants, trial registries, journals, regulators)	N/A
Consent or assent	#26a	Who will obtain informed consent or assent from potential trial participants or authorised surrogates, and how (see Item 32)	11-13
Consent or assent: ancillary studies	#26b	Additional consent provisions for collection and use of participant data and biological specimens in ancillary studies, if applicable	N/A No biological specimens
Confidentiality	#27	How personal information about potential and enrolled participants will be collected, shared, and maintained in order to protect confidentiality before, during, and after the trial	20
Declaration of interests	#28	Financial and other competing interests for principal investigators for the overall trial and each study site	24
Data access	#29	Statement of who will have access to the final trial dataset, and disclosure of contractual agreements that limit such access for investigators	21

For peer review only

Ancillary and post trial care	#30	Provisions, if any, for ancillary and post-trial care, and for compensation to those who suffer harm from trial participation	N/A If required post-trial care to be delivered through local health system
Dissemination policy: trial results	#31a	Plans for investigators and sponsor to communicate trial results to participants, healthcare professionals, the public, and other relevant groups (eg, via publication, reporting in results databases, or other data sharing arrangements), including any publication restrictions	18
Dissemination policy: authorship	#31b	Authorship eligibility guidelines and any intended use of professional writers	25
Dissemination policy: reproducible research	#31c	Plans, if any, for granting public access to the full protocol, participant-level dataset, and statistical code	21
**Appendices**			
Informed consent materials	#32	Model consent form and other related documentation given to participants and authorised surrogates	N/A
Biological specimens	#33	Plans for collection, laboratory evaluation, and storage of biological specimens for genetic or molecular analysis in the current trial and for future use in ancillary studies, if applicable	N/A No biological specimens to be collected

Notes:

- 3: N/A V4 25/2/19
- 13: 13-15, Fig. 3
- 16b: N/A unblinded study
- 17b: N/A unblinded study
- 21b: N/A Single intervention. DSMB and Principal Investigator can stop the study.
- 26b: N/A No biological specimens
- 30: N/A If required post-trial care to be delivered through local health system
- 33: N/A No biological specimens to be collected The SPIRIT checklist is distributed under the terms of the Creative Commons Attribution License CC-BY-ND 3.0. This checklist was completed on 23. January

For peer review only

2020 using https://www.goodreports.org/, a tool made by the EQUATOR Network in collaboration with Penelope.ai

For peer review only
